# Supplementary material for: Fast and automated biomarker detection in breath samples with machine learning
Source: PLoS One. 2022 Apr 12;17(4):e0265399. doi: 10.1371/journal.pone.0265399 (PMC9004778; doi:10.1371/journal.pone.0265399)
Supplement: S1 File — (PDF) [file pone.0265399.s001.pdf]

Supplementary Information

Fast and automated biomarker detection in breath samples with machine learning

A.Skarysz et al.

Contents

1 Target VOCs **3**

2 Abundance matrix **4**

3 Network architectures **5**

4 VGG-8-1D model – Results **22**

5 DenseNet-40-1D model – Results **66**

6 ResNet-34-1D model – Results **72**

7 Intersection of the models – Results **78**

8 Learning curve (VGG-8-1D model) **84**

9 GC-MS instrumentation **85**

1 Target VOCs

S1 Table: Target volotile organic compound properties.

Label - the class label of VOC. *VOC* - target volatile organic compound. *VOCCluster Code* - VOC code derived from [1]. *IUPAC Name* - International Union of Pure and Applied Chemistry name of each VOC. CAS - a unique numerical identifier assigned by the Chemical Abstracts Service to each VOC. *5 Top m/z Peaks* - m/z values of 5 top ions from the VOC mass spectrum, derived from VOC dataset and according to NIST library. *Mean peakRT 1* - mean retention time value of the VOC before GC column change. *Min peakRT 1* - minimum retention time value of the VOC before GC column change. *Max peakRT 1* - maximum retention time value of the VOC before GC column change. *Mean peakRT 2* - mean retention time value of the VOC after GC column change. *Min peakRT 2* - minimum retention time value of the VOC after GC column change. *Max peakRT 2* - maximum retention time value of the VOC after GC column change. *Mean EIC-Area* - mean value of VOC peak area on extracted ion chromatogram (EIC) of a top VOC ion; marker of VOC concentration. *Quantity in VOC dataset* - number of the VOC data-points in the (non-augmented) training set (BS – breath sample, ES – environmental sample). *Quantity in Testset* - number of the target VOC instances reported by the ground truth in the GC-MS samples from test set.

Label	VOC	VOCCluster Code [1]	IUPAC Name	CAS	5 Top m/z Peaks	Mean peakRT 1	Min peakRT 1	Max peakRT 1	Mean peakRT 2	Min peakRT 2	Max peakRT 2	Mean EIC-Area	Quantity in VOC dataset	Quantity in Testset
1	Ethanol	BRI-686-45-46-43-42-44	ethanol	64-17-5	dataset 45 - 46 - 43 - 42 - 44<br>NIST 45 - 46 - 43 - 42 - 41	2.926	2.873	2.942	3.178	3.136	3.236	203414	BS / ES 38 / 15<br>total 53	BS / ES 15 / 5<br>total 20
2	Dimethyl sulfide	BRI-692-62-47-45-46-61	methylsulfanylmethane	75-18-3	dataset 62 - 47 - 45 - 46 - 61<br>NIST 62 - 47 - 45 - 46 - 61	3.059	3.001	3.085	3.390	3.120	3.469	50868	BS / ES 52 / 3<br>total 55	BS / ES 29 / 0<br>total 29
3	2-Methylfuran	BRI-689-82-53-81-50-51	2-methylfuran	534-22-5	dataset 82 - 53 - 81 - 50 - 51<br>NIST 82 - 53 - 81 - 54 - 51	3.409	3.332	3.443	3.931	3.812	4.061	86179	BS / ES 59 / 2<br>total 61	BS / ES 30 / 0<br>total 30
4	Trichloromethane-d	BRI-712-84-86-47-49-88	trichloro(deuterio)methane	865-49-6	dataset 84 - 86 - 47 - 49 - 88<br>NIST 84 - 86 - 47 - 49 - 88	3.488	3.413	3.523	3.959	3.387	4.125	104989	BS / ES 65 / 17<br>total 82	BS / ES 30 / 8<br>total 38
5	Benzene	BRI-707-78-77-51-50-52	benzene	71-43-2	dataset 78 - 77 - 51 - 50 - 52<br>NIST 78 - 77 - 51 - 50 - 52	3.774	3.219	4.273	4.431	4.263	4.648	168917	BS / ES 58 / 16<br>total 74	BS / ES 28 / 8<br>total 36
6	Propionic acid	BRI-724-45-74-73-57-55	propanoic acid	79-09-4	dataset 45 - 74 - 73 - 57 - 55<br>NIST 74 - 45 - 73 - 57 - 55	3.973	3.875	4.325	4.752	4.464	5.580	8586	BS / ES 9 / 13<br>total 22	BS / ES 7 / 4<br>total 11
7	Dimethyl disulfide	BRI-767-94-45-79-46-47	(methyldisulfanyl)methane	624-92-0	dataset 94 - 45 - 79 - 46 - 47<br>NIST 94 - 79 - 45 - 46 - 47	4.693	4.574	4.746	5.656	5.393	5.968	282724	BS / ES 46 / 1<br>total 47	BS / ES 23 / 0<br>total 23
8	Toluene-D8	BRI-780-98-100-42-70-54	1,2,3,4,5-pentadeuterio-6-(trideuteriomethyl)benzene	2037-26-5	dataset 98 - 100 - 42 - 70 - 54<br>NIST 98 - 100 - 42 - 70 - 99	4.936	4.816	5.000	5.969	5.696	6.338	63756	BS / ES 59 / 17<br>total 76	BS / ES 29 / 8<br>total 37
9	Toluene	BRI-782-91-92-65-63-51	toluene	108-88-3	dataset 91 - 92 - 65 - 63 - 51<br>NIST 91 - 92 - 65 - 63 - 51	5.001	4.879	5.067	6.058	5.769	6.423	134743	BS / ES 64 / 17<br>total 81	BS / ES 30 / 8<br>total 38
10	3-Methylthiophene	BRI-792-97-98-45-53-69	3-methylthiophene	616-44-4	dataset 97 - 98 - 45 - 53 - 69<br>NIST 97 - 98 - 45 - 53 - 99	5.214	5.089	5.284	6.318	6.020	6.690	120621	BS / ES 57 / 2<br>total 59	BS / ES 30 / 0<br>total 30
11	Octane	BRI-803-43-41-85-57-71	octane	111-65-9	dataset 43 - 41 - 85 - 57 - 71<br>NIST 43 - 41 - 57 - 85 - 71	5.493	5.307	5.865	6.691	6.192	7.215	13121	BS / ES 36 / 4<br>total 40	BS / ES 18 / 1<br>total 19
12	Hexanal	BRI-808-44-41-56-43-57	hexanal	66-25-1	dataset 44 - 41 - 56 - 43 - 57<br>NIST 44 - 56 - 41 - 43 - 57	5.564	5.472	5.616	6.672	6.357	7.092	11611	BS / ES 42 / 14<br>total 56	BS / ES 19 /6<br>total 25
13	2,4-Dimethylheptane	BRI-805-43-85-41-57-71	2,4-dimethylheptane	2213-23-2	dataset 43 - 85 - 41 - 57 - 71<br>NIST 43 - 85 - 57 - 71 - 41	5.817	5.305	5.917	7.145	6.831	7.599	76791	BS / ES 59 / 11<br>total 70	BS / ES 28 / 5<br>total 33
14	2,4-Dimethylheptane	BRI-835-43-70-55-41-56	2,4-dimethylhept-1-ene	19549-87-2	dataset 43 - 70 - 55 - 41 - 56<br>NIST 43 - 70 - 55 - 57 - 41	6.307	6.085	7.160	7.708	7.301	9.079	33738	BS / ES 59 / 7<br>total 66	BS / ES 30 / 3<br>total 33
15	3-Heptanone	BRI-881-57-41-85-72-43	heptan-3-one	106-35-4	dataset 57 - 41 - 85 - 72 - 43<br>NIST 57 - 85 - 41 - 72 - 114	7.403	7.264	7.511	8.839	8.436	9.380	21034	BS / ES 46 / 13<br>total 59	BS / ES 18 / 5<br>total 23
16	Heptanal	BRI-887-44-41-43-70-55	heptanal	111-71-7	dataset 44 - 41 - 43 - 70 - 55<br>NIST 70 - 41 - 44 - 43 - 55	7.816	7.685	7.886	9.324	8.704	11.035	5482	BS / ES 40 / 15<br>total 55	BS / ES 17 / 6<br>total 23
17	Benzaldehyde	BRI-958-77-105-106-51-50	benzaldehyde	100-52-7	dataset 77 - 105 - 106 - 51 - 50<br>NIST 77 - 106 - 105 - 51 - 50	9.545	9.369	9.646	11.060	10.579	11.689	52375	BS / ES 65 / 17<br>total 82	BS / ES 30 / 8<br>total 38
18	Benzonitrile	BRI-984-103-76-50-51-75	benzonitrile	100-47-0	dataset 103 - 76 - 50 - 51 - 75<br>NIST 103 - 76 - 50 - 51 - 75	10.197	10.029	10.301	11.803	11.285	12.430	18154	BS / ES 60 / 17<br>total 77	BS / ES 29 / 7<br>total 36
19	Octanal	BRI-998-41-43-44-57-55	octanal	124-13-0	dataset 41 - 43 - 44 - 57 - 55<br>NIST 43 - 44 - 41 - 56 - 84	10.628	10.438	11.592	12.235	11.755	12.966	6407	BS / ES 45 / 14<br>total 59	BS / ES 20 / 6<br>total 26
20	Limonene	BRI-1002-68-67-93-79-41	1-methyl-4-prop-1-en-2-ylcyclohexene	138-86-3	dataset 68 - 67 - 93 - 79 - 41<br>NIST 68 - 93 - 67 - 79 - 136	11.298	11.079	11.421	13.115	12.613	13.756	156328	BS / ES 65 / 14<br>total 79	BS / ES 30 / 7<br>total 37
21	2-Ethylhexanol	BRI-999-57-41-43-55-56	2-ethylhexan-1-ol	104-76-7	dataset 57 - 41 - 43 - 55 - 56<br>NIST 57 - 41 - 43 - 55 - 56	11.543	10.883	12.898	13.026	12.529	14.059	104600	BS / ES 45 / 14<br>total 59	BS / ES 19 / 8<br>total 27
22	Nonanal	BRI-1102-41-57-43-55-56	nonanal	124-19-6	dataset 41 - 57 - 43 - 55 - 56<br>NIST 57 - 41 - 43 - 56 - 44	13.559	13.335	13.657	15.286	14.781	15.962	20544	BS / ES 57 / 15<br>total 72	BS / ES 25 / 7<br>total 32
23	Dodecane	BRI-1171-57-43-41-71-85	dodecane	112-40-3	dataset 57 - 43 - 41 - 71 - 85<br>NIST 57 - 43 - 71 - 41 - 85	16.162	15.928	16.303	18.069	17.562	18.735	65787	BS / ES 64 / 14<br>total 78	BS / ES 30 / 6<br>total 36
24	Decanal	BRI-1217-41-43-57-55-44	decanal	112-31-2	dataset 41 - 43 - 57 - 55 - 44<br>NIST 43 - 41 - 57 - 55 - 44	16.507	16.316	16.603	18.289	17.748	18.974	9868	BS / ES 38 / 13<br>total 51	BS / ES 18 / 6<br>total 24
25	2-Phenoxyethanol	BRI-1194-94-77-138-66-51	2-phenoxyethanol	122-99-6	dataset 94 - 77 - 138 - 66 - 51<br>NIST 94 - 77 - 138 - 66 - 51	16.897	16.835	17.016	18.733	18.223	19.508	21743	BS / ES 21 / 12<br>total 33	BS / ES 2 / 5<br>total 7
26	Phthalic acid	BRI-1325-104-76-50-74-148	phthalic acid	88-99-3	dataset 104 - 76 - 50 - 74 - 148<br>NIST 104 - 76 - 50 - 148 - 74	19.695	19.510	19.803	21.464	20.923	22.324	17780	BS / ES 41 / 16<br>total 57	BS / ES 15 / 7<br>total 22
27	Tetradecane	BRI-1415-57-43-71-41-85	tetradecane	629-59-4	dataset 57 - 43 - 71 - 41 - 85<br>NIST 57 - 43 - 71 - 85 - 41	21.610	21.375	21.760	23.544	23.005	24.224	45778	BS / ES 61 / 14<br>total 75	BS / ES 27 / 5<br>total 32
28	1,4-Diacetylbenzene	BRI-1459-147-43-91-119-162	1-(4-acetylphenyl)ethanone	1009-61-6	dataset 147 - 43 - 91 - 119 - 162<br>NIST 147 - 43 - 91 - 119 - 162	22.753	22.518	22.875	24.588	24.020	25.402	37343	BS / ES 47 / 8<br>total 55	BS / ES 13 / 4<br>total 17
29	3,3,6,6-Tetraphenyl-1,2,4,5-tetroxane	BRI-1644-105-77-182-51-50	3,3,6,6-tetraphenyl-1,2,4,5-tetraoxane	16204-36-7	dataset 105 - 77 - 182 - 51 - 50<br>NIST 105 - 77 - 182 - 51 - 107	27.550	27.338	27.678	29.416	28.829	30.272	4301	BS / ES 49 / 12<br>total 61	BS / ES 25 / 6<br>total 31
30	2,5-Diphenyl-1,4-benzoquinone	BRI-2344-102-260-76-232-0	2,5-diphenylcyclohexa-2,5-diene-1,4-dione	844-51-9	dataset 102 - 260 - 76 - 232 - 0<br>NIST 102 - 260 - 259 - 232 - 76	41.891	41.629	42.043	43.825	43.187	44.741	84612	BS / ES 58 / 16<br>total 74	BS / ES 27 / 5<br>total 32
													**Total Trainset** **1868**	**Total Testset** **845**
													+	
													Negative class 1868	

[1] - Alkhalifah Y. et al., 2009. *VOCCluster: Untargeted Metabolomics Feature Clustering Approach for Clinical Breath Gas Chromatography - Mass Spectrometry Data*

2 Abundance matrix

S2 Figure: Example of the abundance matrix (fragment). Each column of the abundance matrix represents one ion channel m/z, whereas each row contains a mass spectrum delivered at a specific RT.

RT (minutes)	40	41	42	43	44	45	46	47
0.19202	0	629	360	698	960	556	20	176
0.19457	0	632	371	685	926	535	12	208
0.19712	0	640	342	660	954	514	0	197
0.19967	0	663	296	618	1037	528	23	182
0.20222	0	630	256	613	1065	538	22	193
0.20487	0	563	242	657	985	529	23	205
0.20753	0	516	264	683	884	520	43	184
0.21020	0	503	295	662	856	514	64	161
0.21287	0	504	327	629	914	521	67	164
0.21552	0	471	345	609	1026	537	58	195
0.21818	0	468	333	565	1104	554	0	188
0.22085	0	549	302	517	1074	518	0	139
0.22350	0	625	300	510	987	467	0	103
0.22617	0	613	292	561	932	487	81	105
0.22883	0	551	255	614	927	563	50	123
0.23150	0	537	239	604	931	630	11	127
0.23415	0	541	275	557	904	614	0	116
0.23682	0	584	311	542	868	552	0	126
0.23947	0	633	308	548	896	547	7	178
0.24213	0	618	274	573	970	576	8	224
0.24478	0	541	250	649	1007	546	14	202
0.24745	0	494	248	697	1010	481	16	133
0.25012	0	525	256	649	1047	442	16	84
0.25278	0	548	280	557	1111	447	27	71
0.25543	0	477	277	504	1107	440	43	78
0.25810	0	413	229	485	1017	463	49	94
0.26075	0	471	197	520	995	478	48	106
0.26342	0	559	201	581	1108	462	43	113
0.26608	0	548	212	575	1178	444	30	126
0.26873	0	463	212	552	1106	438	0	122
0.27140	0	403	215	595	1012	431	28	105
0.27407	0	406	222	609	938	449	43	109
0.27672	0	458	226	576	835	516	41	128
0.27938	0	503	227	596	770	562	0	136
0.28203	0	521	234	624	816	535	0	133
0.28470	0	560	242	603	923	458	0	138
0.28737	0	596	270	600	1042	428	6	137
0.29002	0	603	311	624	1092	426	9	109
0.29268	0	620	342	589	1033	384	13	91
0.29535	0	628	348	507	953	350	22	95
0.29800	0	603	316	445	883	349	41	100
0.30067	0	530	265	442	891	347	105	99
0.30332	0	442	231	477	922	328	0	98
0.30598	0	412	235	522	883	329	0	92
0.30863	0	444	273	557	846	374	19	88
0.31130	0	491	314	584	848	408	12	99
0.31397	0	519	308	587	863	377	0	109
0.31662	0	547	252	555	924	321	0	102
0.31928	0	604	195	508	1006	321	16	83

3 Network architectures

S3.1 Table: VGG-4-1D network architecture. Network was implemented using Keras with Tensorflow backend and trained for 10 epochs with 32 batch size.

	Layer	(type)	Output Shape	# Param	Filter size	Stride	Padding	Activation
	input_1	(InputLayer)	(None, 80, 411, 1)	0	—	—	—	—
1	conv2d_1	(Conv2D)	(None, 78, 411, 32)	128	(3, 1)	(1, 1)	valid	relu
2	conv2d_2	(Conv2D)	(None, 76, 411, 32)	3104	(3, 1)	(1, 1)	valid	relu
	max_pooling2d_1	(MaxPooling2D)	(None, 38, 411, 32)	0	(2, 1)	(2, 1)	valid	—
	dropout_1	(Dropout)	(None, 38, 411, 32)	0	—	—	—	—
	flatten_1	(Flatten)	(None, 499776)	0	—	—	—	—
3	dense_1	(Dense)	(None, 256)	127942912	—	—	—	relu
	dropout_4	(Dropout)	(None, 256)	0	—	—	—	—
4	dense_2	(Dense)	(None, 31)	7967	—	—	—	softmax

**Total parameters:**	**127,954,111**
**Trainable parameters:**	**127,954,111**
**Non-trainable parameters:**	**0**

S3.2 Table: VGG-4-2D network architecture. Network was implemented using Keras with Tensorflow backend and trained for 10 epochs with 32 batch size.

	Layer	(type)	Output Shape	# Param	Filter size	Stride	Padding	Activation
	input_1	(InputLayer)	(None, 80, 411, 1)	0	—	—	—	—
1	conv2d_1	(Conv2D)	(None, 78, 409, 32)	320	(3, 3)	(1, 1)	valid	relu
2	conv2d_2	(Conv2D)	(None, 76, 407, 32)	9248	(3, 3)	(1, 1)	valid	relu
	max_pooling2d_1	(MaxPooling2D)	(None, 38, 203, 32)	0	(2, 2)	(2, 2)	valid	—
	dropout_1	(Dropout)	(None, 38, 203, 32)	0	—	—	—	—
	flatten_1	(Flatten)	(None, 246848)	0	—	—	—	—
3	dense_1	(Dense)	(None, 256)	63193344	—	—	—	relu
	dropout_4	(Dropout)	(None, 256)	0	—	—	—	—
4	dense_2	(Dense)	(None, 31)	7967	—	—	—	softmax

**Total parameters:**	**63,210,879**
**Trainable parameters:**	**63,210,879**
**Non-trainable parameters:**	**0**

S3.3 Table: VGG-6-1D network architecture. Network was implemented using Keras with Tensorflow backend and trained for 10 epochs with 32 batch size.

Layer	(type)	Output Shape	# Param	Filter size	Stride	Padding	Activation
input_1	(InputLayer)	(None, 80, 411, 1)	0	—	—	—	—
1 conv2d_1	(Conv2D)	(None, 78, 411, 32)	128	(3, 1)	(1, 1)	valid	relu
2 conv2d_2	(Conv2D)	(None, 76, 411, 32)	3104	(3, 1)	(1, 1)	valid	relu
max_pooling2d_1	(MaxPooling2D)	(None, 38, 411, 32)	0	(2, 1)	(2, 1)	valid	—
dropout_1	(Dropout)	(None, 38, 411, 32)	0	—	—	—	—
3 conv2d_3	(Conv2D)	(None, 36, 411, 64)	6208	(3, 1)	(1, 1)	valid	relu
4 conv2d_4	(Conv2D)	(None, 34, 411, 64)	12352	(3, 1)	(1, 1)	valid	relu
max_pooling2d_2	(MaxPooling2D)	(None, 17, 411, 64)	0	(2, 1)	(2, 1)	valid	—
dropout_2	(Dropout)	(None, 17, 411, 64)	0	—	—	—	—
flatten_1	(Flatten)	(None, 447168)	0	—	—	—	—
5 dense_1	(Dense)	(None, 256)	114475264	—	—	—	relu
dropout_4	(Dropout)	(None, 256)	0	—	—	—	—
6 dense_2	(Dense)	(None, 31)	7967	—	—	—	softmax

**Total parameters:**	**114,505,023**
**Trainable parameters:**	**114,505,023**
**Non-trainable parameters:**	**0**

S3.4 Table: VGG-6-2D network architecture. Network was implemented using Keras with Tensorflow backend and trained for 10 epochs with 32 batch size.

	Layer	(type)	Output Shape	# Param	Filter size	Stride	Padding	Activation
	input_1	(InputLayer)	(None, 80, 411, 1)	0	—	—	—	—
1	conv2d_1	(Conv2D)	(None, 78, 409, 32)	320	(3, 3)	(1, 1)	valid	relu
2	conv2d_2	(Conv2D)	(None, 76, 407, 32)	9248	(3, 3)	(1, 1)	valid	relu
	max_pooling2d_1	(MaxPooling2D)	(None, 38, 203, 32)	0	(2, 2)	(2, 2)	valid	—
	dropout_1	(Dropout)	(None, 38, 203, 32)	0	—	—	—	—
3	conv2d_3	(Conv2D)	(None, 36, 201, 64)	18496	(3, 3)	(1, 1)	valid	relu
4	conv2d_4	(Conv2D)	(None, 34, 199, 64)	36928	(3, 3)	(1, 1)	valid	relu
	max_pooling2d_2	(MaxPooling2D)	(None, 17, 99, 64)	0	(2, 2)	(2, 2)	valid	—
	dropout_2	(Dropout)	(None, 17, 99, 64)	0	—	—	—	—
	flatten_1	(Flatten)	(None, 107712)	0	—	—	—	—
5	dense_1	(Dense)	(None, 256)	27574528	—	—	—	relu
	dropout_4	(Dropout)	(None, 256)	0	—	—	—	—
6	dense_2	(Dense)	(None, 31)	7967	—	—	—	softmax

**Total parameters:**	**27,647,487**
**Trainable parameters:**	**27,647,487**
**Non-trainable parameters:**	**0**

S3.5 Table: VGG-8-1D network architecture. Network was implemented using Keras with Tensorflow backend and trained for 10 epochs with 32 batch size.

	Layer	(type)	Output Shape	# Param	Filter size	Stride	Padding	Activation
	input_1	(InputLayer)	(None, 80, 411, 1)	0	—	—	—	—
1	conv2d_1	(Conv2D)	(None, 78, 411, 32)	128	(3, 1)	(1, 1)	valid	relu
2	conv2d_2	(Conv2D)	(None, 76, 411, 32)	3104	(3, 1)	(1, 1)	valid	relu
	max_pooling2d_1	(MaxPooling2D)	(None, 38, 411, 32)	0	(2, 1)	(2, 1)	valid	—
	dropout_1	(Dropout)	(None, 38, 411, 32)	0	—	—	—	—
3	conv2d_3	(Conv2D)	(None, 36, 411, 64)	6208	(3, 1)	(1, 1)	valid	relu
4	conv2d_4	(Conv2D)	(None, 34, 411, 64)	12352	(3, 1)	(1, 1)	valid	relu
	max_pooling2d_2	(MaxPooling2D)	(None, 17, 411, 64)	0	(2, 1)	(2, 1)	valid	—
	dropout_2	(Dropout)	(None, 17, 411, 64)	0	—	—	—	—
5	conv2d_5	(Conv2D)	(None, 15, 411, 128)	24704	(3, 1)	(1, 1)	valid	relu
6	conv2d_6	(Conv2D)	(None, 13, 411, 128)	49280	(3, 1)	(1, 1)	valid	relu
	max_pooling2d_3	(MaxPooling2D)	(None, 6, 411, 128)	0	(2, 1)	(2, 1)	valid	—
	dropout_3	(Dropout)	(None, 6, 411, 128)	0	—	—	—	—
	flatten_1	(Flatten)	(None, 315648)	0	—	—	—	—
7	dense_1	(Dense)	(None, 256)	80806144	—	—	—	relu
	dropout_4	(Dropout)	(None, 256)	0	—	—	—	—
8	dense_2	(Dense)	(None, 31)	7967	—	—	—	softmax

**Total parameters:**	**80,909,887**
**Trainable parameters:**	**80,909,887**
**Non-trainable parameters:**	**0**

S3.6 Table: VGG-8-2D network architecture. Network was implemented using Keras with Tensorflow backend and trained for 10 epochs with 32 batch size.

Layer		(type)	Output Shape	# Param	Filter size	Stride	Padding	Activation
	input_1	(InputLayer)	(None, 80, 411, 1)	0	—	—	—	—
1	conv2d_1	(Conv2D)	(None, 78, 409, 32)	320	(3, 3)	(1, 1)	valid	relu
2	conv2d_2	(Conv2D)	(None, 76, 407, 32)	9248	(3, 3)	(1, 1)	valid	relu
	max_pooling2d_1	(MaxPooling2	(None, 38, 203, 32)	0	(2, 2)	(2, 2)	valid	—
	dropout_1	(Dropout)	(None, 38, 203, 32)	0	—	—	—	—
3	conv2d_3	(Conv2D)	(None, 36, 201, 64)	18496	(3, 3)	(1, 1)	valid	relu
4	conv2d_4	(Conv2D)	(None, 34, 199, 64)	36928	(3, 3)	(1, 1)	valid	relu
	max_pooling2d_2	(MaxPooling2	(None, 17, 99, 64)	0	(2, 2)	(2, 2)	valid	—
	dropout_2	(Dropout)	(None, 17, 99, 64)	0	—	—	—	—
5	conv2d_5	(Conv2D)	(None, 15, 97, 128)	73856	(3, 3)	(1, 1)	valid	relu
6	conv2d_6	(Conv2D)	(None, 13, 95, 128)	147584	(3, 3)	(1, 1)	valid	relu
	max_pooling2d_3	(MaxPooling2	(None, 6, 47, 128)	0	(2, 2)	(2, 2)	valid	—
	dropout_3	(Dropout)	(None, 6, 47, 128)	0	—	—	—	—
	flatten_1	(Flatten)	(None, 36096)	0	—	—	—	—
7	dense_1	(Dense)	(None, 256)	9240832	—	—	—	relu
	dropout_4	(Dropout)	(None, 256)	0	—	—	—	—
8	dense_2	(Dense)	(None, 31)	7967	—	—	—	softmax

**Total parameters:**	**9,535,231**
**Trainable parameters:**	**9,535,231**
**Non-trainable parameters:**	**0**

S3.7 Table: VGG-16-2D network architecture. Network was implemented using Keras with Tensorflow backend and trained for 10 epochs with 32 batch size.

	Layer	(type)	Output Shape	# Param	Filter size	Stride	Padding	Activation
	input_1	(InputLayer)	(None, 80, 411, 1)	0	—	—	—	—
1	conv2d_1	(Conv2D)	(None, 80, 411, 64)	640	(3, 3)	(1, 1)	same	—
	batch_normalization_1	(BatchNormalization)	(None, 80, 411, 64)	256	—	—	—	—
	activation_1	(Activation)	(None, 80, 411, 64)	0	—	—	—	relu
2	conv2d_2	(Conv2D)	(None, 80, 411, 64)	36928	(3, 3)	(1, 1)	same	—
	batch_normalization_2	(BatchNormalization)	(None, 80, 411, 64)	256	—	—	—	—
	activation_2	(Activation)	(None, 80, 411, 64)	0	—	—	—	relu
	max_pooling2d_1	(MaxPooling2D)	(None, 40, 205, 64)	0	(2, 2)	(2, 2)	valid	—
3	conv2d_3	(Conv2D)	(None, 40, 205, 128)	73856	(3, 3)	(1, 1)	same	—
	batch_normalization_3	(BatchNormalization)	(None, 40, 205, 128)	512	—	—	—	—
	activation_3	(Activation)	(None, 40, 205, 128)	0	—	—	—	relu
4	conv2d_4	(Conv2D)	(None, 40, 205, 128)	147584	(3, 3)	(1, 1)	same	—
	batch_normalization_4	(BatchNormalization)	(None, 40, 205, 128)	512	—	—	—	—
	activation_4	(Activation)	(None, 40, 205, 128)	0	—	—	—	relu
	max_pooling2d_2	(MaxPooling2D)	(None, 20, 102, 128)	0	(2, 2)	(2, 2)	valid	—
5	conv2d_5	(Conv2D)	(None, 20, 102, 256)	295168	(3, 3)	(1, 1)	same	—
	batch_normalization_5	(BatchNormalization)	(None, 20, 102, 256)	1024	—	—	—	—
	activation_5	(Activation)	(None, 20, 102, 256)	0	—	—	—	relu
6	conv2d_6	(Conv2D)	(None, 20, 102, 256)	590080	(3, 3)	(1, 1)	same	—
	batch_normalization_6	(BatchNormalization)	(None, 20, 102, 256)	1024	—	—	—	—
	activation_6	(Activation)	(None, 20, 102, 256)	0	—	—	—	relu
7	conv2d_7	(Conv2D)	(None, 20, 102, 256)	590080	(3, 3)	(1, 1)	same	—
	batch_normalization_7	(BatchNormalization)	(None, 20, 102, 256)	1024	—	—	—	—
	activation_7	(Activation)	(None, 20, 102, 256)	0	—	—	—	relu
	max_pooling2d_3	(MaxPooling2D)	(None, 10, 51, 256)	0	(2, 2)	(2, 2)	valid	—
8	conv2d_8	(Conv2D)	(None, 10, 51, 512)	1180160	(3, 3)	(1, 1)	same	—
	batch_normalization_8	(BatchNormalization)	(None, 10, 51, 512)	2048	—	—	—	—
	activation_8	(Activation)	(None, 10, 51, 512)	0	—	—	—	relu
9	conv2d_9	(Conv2D)	(None, 10, 51, 512)	2359808	(3, 3)	(1, 1)	same	—
	batch_normalization_9	(BatchNormalization)	(None, 10, 51, 512)	2048	—	—	—	—
	activation_9	(Activation)	(None, 10, 51, 512)	0	—	—	—	relu
10	conv2d_10	(Conv2D)	(None, 10, 51, 512)	2359808	(3, 3)	(1, 1)	same	—
	batch_normalization_10	(BatchNormalization)	(None, 10, 51, 512)	2048	—	—	—	—
	activation_10	(Activation)	(None, 10, 51, 512)	0	—	—	—	relu
	max_pooling2d_4	(MaxPooling2D)	(None, 5, 25, 512)	0	(2, 2)	(2, 2)	valid	—
11	conv2d_11	(Conv2D)	(None, 5, 25, 512)	2359808	(3, 3)	(1, 1)	same	—
	batch_normalization_11	(BatchNormalization)	(None, 5, 25, 512)	2048	—	—	—	—
	activation_11	(Activation)	(None, 5, 25, 512)	0	—	—	—	relu
12	conv2d_12	(Conv2D)	(None, 5, 25, 512)	2359808	(3, 3)	(1, 1)	same	—
	batch_normalization_12	(BatchNormalization)	(None, 5, 25, 512)	2048	—	—	—	—
	activation_12	(Activation)	(None, 5, 25, 512)	0	—	—	—	relu
13	conv2d_13	(Conv2D)	(None, 5, 25, 512)	2359808	(3, 3)	(1, 1)	same	—
	batch_normalization_13	(BatchNormalization)	(None, 5, 25, 512)	2048	—	—	—	—
	activation_13	(Activation)	(None, 5, 25, 512)	0	—	—	—	relu
	max_pooling2d_5	(MaxPooling2D)	(None, 2, 12, 512)	0	(2, 2)	(2, 2)	valid	—
	flatten_1	(Flatten)	(None, 12288)	0	—	—	—	—
14	dense_1	(Dense)	(None, 4096)	50335744	—	—	—	relu
	dropout_1	(Dropout)	(None, 4096)	0	—	—	—	—
15	dense_2	(Dense)	(None, 4096)	16781312	—	—	—	relu
	dropout_2	(Dropout)	(None, 4096)	0	—	—	—	—
16	dense_3	(Dense)	(None, 31)	127007	—	—	—	softmax

**Total parameters:**	**81,974,495**
**Trainable parameters:**	**81,966,047**
**Non-trainable parameters:**	**8448**

S3.8 Table: DenseNet-40-1D network architecture. Network was implemented using Keras with Tensorflow backend and trained for 10 epochs with 32 batch size. The network architectre was adopted from http://github.com/titu1994/DenseNet.

Layer	(type)	Output Shape	# Param	Filter size	Stride	Padding	Activation	Connected to
input_1	(InputLayer)	(None, 80, 411, 1)	0	—	—	—	—	—
1 conv2d_1	(Conv2D)	(None, 40, 411, 24)	168	(7, 1)	(2, 1)	same	—	input_1
batch_normalization_1	(BatchNormalization)	(None, 40, 411, 24)	96	—	—	—	—	conv2d_1
activation_1	(Activation)	(None, 40, 411, 24)	0	—	—	—	—	batch_normalization_1
max_pooling2d_1	(MaxPooling2D)	(None, 20, 411, 24)	0	(3, 1)	(2, 1)	same	—	activation_1
batch_normalization_2	(BatchNormalization)	(None, 20, 411, 24)	96	—	—	—	—	max_pooling2d_1
activation_2	(Activation)	(None, 20, 411, 24)	0	—	—	—	relu	batch_normalization_2
2 conv2d_2	(Conv2D)	(None, 20, 411, 12)	864	(3, 1)	(3, 1)	same	—	activation_2
concatenate_1	(Concatenate)	(None, 20, 411, 36)	0	—	—	—	—	max_pooling2d_1<br>conv2d_2
batch_normalization_3	(BatchNormalization)	(None, 20, 411, 36)	144	—	—	—	—	concatenate_1
activation_3	(Activation)	(None, 20, 411, 36)	0	—	—	—	relu	batch_normalization_3
3 conv2d_3	(Conv2D)	(None, 20, 411, 12)	1296	(3, 1)	(3, 1)	same	—	activation_3
concatenate_2	(Concatenate)	(None, 20, 411, 48)	0	—	—	—	—	concatenate_1<br>conv2d_3
batch_normalization_4	(BatchNormalization)	(None, 20, 411, 48)	192	—	—	—	—	concatenate_2
activation_4	(Activation)	(None, 20, 411, 48)	0	—	—	—	relu	batch_normalization_4
4 conv2d_4	(Conv2D)	(None, 20, 411, 12)	1728	(3, 1)	(3, 1)	same	—	activation_4
concatenate_3	(Concatenate)	(None, 20, 411, 60)	0	—	—	—	—	concatenate_2<br>conv2d_4
batch_normalization_5	(BatchNormalization)	(None, 20, 411, 60)	240	—	—	—	—	concatenate_3
activation_5	(Activation)	(None, 20, 411, 60)	0	—	—	—	relu	batch_normalization_5
5 conv2d_5	(Conv2D)	(None, 20, 411, 12)	2160	(3, 1)	(3, 1)	same	—	activation_5
concatenate_4	(Concatenate)	(None, 20, 411, 72)	0	—	—	—	—	concatenate_3<br>conv2d_5
batch_normalization_6	(BatchNormalization)	(None, 20, 411, 72)	288	—	—	—	—	concatenate_4
activation_6	(Activation)	(None, 20, 411, 72)	0	—	—	—	relu	batch_normalization_6
6 conv2d_6	(Conv2D)	(None, 20, 411, 12)	2592	(3, 1)	(3, 1)	same	—	activation_6
concatenate_5	(Concatenate)	(None, 20, 411, 84)	0	—	—	—	—	concatenate_4<br>conv2d_6
batch_normalization_7	(BatchNormalization)	(None, 20, 411, 84)	336	—	—	—	—	concatenate_5
activation_7	(Activation)	(None, 20, 411, 84)	0	—	—	—	relu	batch_normalization_7
7 conv2d_7	(Conv2D)	(None, 20, 411, 12)	3024	(3, 1)	(3, 1)	same	—	activation_7
concatenate_6	(Concatenate)	(None, 20, 411, 96)	0	—	—	—	—	concatenate_5<br>conv2d_7
batch_normalization_8	(BatchNormalization)	(None, 20, 411, 96)	384	—	—	—	—	concatenate_6
activation_8	(Activation)	(None, 20, 411, 96)	0	—	—	—	relu	batch_normalization_8
8 conv2d_8	(Conv2D)	(None, 20, 411, 12)	3456	(3, 1)	(3, 1)	same	—	activation_8
concatenate_7	(Concatenate)	(None, 20, 411, 108)	0	—	—	—	—	concatenate_6<br>conv2d_8
batch_normalization_9	(BatchNormalization)	(None, 20, 411, 108)	432	—	—	—	—	concatenate_7
activation_9	(Activation)	(None, 20, 411, 108)	0	—	—	—	relu	batch_normalization_9
9 conv2d_9	(Conv2D)	(None, 20, 411, 12)	3888	(3, 1)	(3, 1)	same	—	activation_9
concatenate_8	(Concatenate)	(None, 20, 411, 120)	0	—	—	—	—	concatenate_7<br>conv2d_9
batch_normalization_10	(BatchNormalization)	(None, 20, 411, 120)	480	—	—	—	—	concatenate_8
activation_10	(Activation)	(None, 20, 411, 120)	0	—	—	—	relu	batch_normalization_10
10 conv2d_10	(Conv2D)	(None, 20, 411, 12)	4320	(3, 1)	(3, 1)	same	—	activation_10
concatenate_9	(Concatenate)	(None, 20, 411, 132)	0	—	—	—	—	concatenate_8<br>conv2d_10
batch_normalization_11	(BatchNormalization)	(None, 20, 411, 132)	528	—	—	—	—	concatenate_9
activation_11	(Activation)	(None, 20, 411, 132)	0	—	—	—	relu	batch_normalization_11
11 conv2d_11	(Conv2D)	(None, 20, 411, 12)	4752	(3, 1)	(3, 1)	same	—	activation_11
concatenate_10	(Concatenate)	(None, 20, 411, 144)	0	—	—	—	—	concatenate_9<br>conv2d_11
batch_normalization_12	(BatchNormalization)	(None, 20, 411, 144)	576	—	—	—	—	concatenate_10
activation_12	(Activation)	(None, 20, 411, 144)	0	—	—	—	relu	batch_normalization_12
12 conv2d_12	(Conv2D)	(None, 20, 411, 12)	5184	(3, 1)	(3, 1)	same	—	activation_12
concatenate_11	(Concatenate)	(None, 20, 411, 156)	0	—	—	—	—	concatenate_10<br>conv2d_12
batch_normalization_13	(BatchNormalization)	(None, 20, 411, 156)	624	—	—	—	—	concatenate_11
activation_13	(Activation)	(None, 20, 411, 156)	0	—	—	—	relu	batch_normalization_13
13 conv2d_13	(Conv2D)	(None, 20, 411, 12)	5616	(3, 1)	(3, 1)	same	—	activation_13
concatenate_12	(Concatenate)	(None, 20, 411, 168)	0	—	—	—	—	concatenate_11<br>conv2d_13
batch_normalization_14	(BatchNormalization)	(None, 20, 411, 168)	672	—	—	—	—	concatenate_12
activation_14	(Activation)	(None, 20, 411, 168)	0	—	—	—	relu	batch_normalization_14
14 conv2d_14	(Conv2D)	(None, 20, 411, 168)	28224	(1, 1)	(1, 1)	same	—	activation_14
average_pooling2d_1	(AveragePooling2D)	(None, 10, 411, 168)	0	(2, 1)	(2, 1)	valid	—	conv2d_14
batch_normalization_15	(BatchNormalization)	(None, 10, 411, 168)	672	—	—	—	—	average_pooling2d_1
activation_15	(Activation)	(None, 10, 411, 168)	0	—	—	—	relu	batch_normalization_15

15	conv2d_15	(Conv2D)	(None, 10, 411, 12)	6048	(3, 1)	(3, 1)	same	—	activation_15
	concatenate_13	(Concatenate)	(None, 10, 411, 180)	0	—	—	—	—	average_pooling2d_1<br>conv2d_15
	batch_normalization_16	(BatchNormalization)	(None, 10, 411, 180)	720	—	—	—	—	concatenate_13
	activation_16	(Activation)	(None, 10, 411, 180)	0	—	—	—	relu	batch_normalization_16
16	conv2d_16	(Conv2D)	(None, 10, 411, 12)	6480	(3, 1)	(3, 1)	same	—	activation_16
	concatenate_14	(Concatenate)	(None, 10, 411, 192)	0	—	—	—	—	concatenate_13<br>conv2d_16
	batch_normalization_17	(BatchNormalization)	(None, 10, 411, 192)	768	—	—	—	—	concatenate_14
	activation_17	(Activation)	(None, 10, 411, 192)	0	—	—	—	relu	batch_normalization_17
17	conv2d_17	(Conv2D)	(None, 10, 411, 12)	6912	(3, 1)	(3, 1)	same	—	activation_17
	concatenate_15	(Concatenate)	(None, 10, 411, 204)	0	—	—	—	—	concatenate_14<br>conv2d_17
	batch_normalization_18	(BatchNormalization)	(None, 10, 411, 204)	816	—	—	—	—	concatenate_15
	activation_18	(Activation)	(None, 10, 411, 204)	0	—	—	—	relu	batch_normalization_18
18	conv2d_18	(Conv2D)	(None, 10, 411, 12)	7344	(3, 1)	(3, 1)	same	—	activation_18
	concatenate_16	(Concatenate)	(None, 10, 411, 216)	0	—	—	—	—	concatenate_15<br>conv2d_18
	batch_normalization_19	(BatchNormalization)	(None, 10, 411, 216)	864	—	—	—	—	concatenate_16
	activation_19	(Activation)	(None, 10, 411, 216)	0	—	—	—	relu	batch_normalization_19
19	conv2d_19	(Conv2D)	(None, 10, 411, 12)	7776	(3, 1)	(3, 1)	same	—	activation_19
	concatenate_17	(Concatenate)	(None, 10, 411, 228)	0	—	—	—	—	concatenate_16<br>conv2d_19
	batch_normalization_20	(BatchNormalization)	(None, 10, 411, 228)	912	—	—	—	—	concatenate_17
	activation_20	(Activation)	(None, 10, 411, 228)	0	—	—	—	relu	batch_normalization_20
20	conv2d_20	(Conv2D)	(None, 10, 411, 12)	8208	(3, 1)	(3, 1)	same	—	activation_20
	concatenate_18	(Concatenate)	(None, 10, 411, 240)	0	—	—	—	—	concatenate_17<br>conv2d_20
	batch_normalization_21	(BatchNormalization)	(None, 10, 411, 240)	960	—	—	—	—	concatenate_18
	activation_21	(Activation)	(None, 10, 411, 240)	0	—	—	—	relu	batch_normalization_21
21	conv2d_21	(Conv2D)	(None, 10, 411, 12)	8640	(3, 1)	(3, 1)	same	—	activation_21
	concatenate_19	(Concatenate)	(None, 10, 411, 252)	0	—	—	—	—	concatenate_18<br>conv2d_21
	batch_normalization_22	(BatchNormalization)	(None, 10, 411, 252)	1008	—	—	—	—	concatenate_19
	activation_22	(Activation)	(None, 10, 411, 252)	0	—	—	—	relu	batch_normalization_22
22	conv2d_22	(Conv2D)	(None, 10, 411, 12)	9072	(3, 1)	(3, 1)	same	—	activation_22
	concatenate_20	(Concatenate)	(None, 10, 411, 264)	0	—	—	—	—	concatenate_19<br>conv2d_22
	batch_normalization_23	(BatchNormalization)	(None, 10, 411, 264)	1056	—	—	—	—	concatenate_20
	activation_23	(Activation)	(None, 10, 411, 264)	0	—	—	—	relu	batch_normalization_23
23	conv2d_23	(Conv2D)	(None, 10, 411, 12)	9504	(3, 1)	(3, 1)	same	—	activation_23
	concatenate_21	(Concatenate)	(None, 10, 411, 276)	0	—	—	—	—	concatenate_20<br>conv2d_23
	batch_normalization_24	(BatchNormalization)	(None, 10, 411, 276)	1104	—	—	—	—	concatenate_21
	activation_24	(Activation)	(None, 10, 411, 276)	0	—	—	—	relu	batch_normalization_24
24	conv2d_24	(Conv2D)	(None, 10, 411, 12)	9936	(3, 1)	(3, 1)	same	—	activation_24
	concatenate_22	(Concatenate)	(None, 10, 411, 288)	0	—	—	—	—	concatenate_21<br>conv2d_24
	batch_normalization_25	(BatchNormalization)	(None, 10, 411, 288)	1152	—	—	—	—	concatenate_22
	activation_25	(Activation)	(None, 10, 411, 288)	0	—	—	—	relu	batch_normalization_25
25	conv2d_25	(Conv2D)	(None, 10, 411, 12)	10368	(3, 1)	(3, 1)	same	—	activation_25
	concatenate_23	(Concatenate)	(None, 10, 411, 300)	0	—	—	—	—	concatenate_22<br>conv2d_25
	batch_normalization_26	(BatchNormalization)	(None, 10, 411, 300)	1200	—	—	—	—	concatenate_23
	activation_26	(Activation)	(None, 10, 411, 300)	0	—	—	—	relu	batch_normalization_26
26	conv2d_26	(Conv2D)	(None, 10, 411, 12)	10800	(3, 1)	(3, 1)	same	—	activation_26
	concatenate_24	(Concatenate)	(None, 10, 411, 312)	0	—	—	—	—	concatenate_23<br>conv2d_26
	batch_normalization_27	(BatchNormalization)	(None, 10, 411, 312)	1248	—	—	—	—	concatenate_24
	activation_27	(Activation)	(None, 10, 411, 312)	0	—	—	—	relu	batch_normalization_27
27	conv2d_27	(Conv2D)	(None, 10, 411, 312)	97344	(1, 1)	(1, 1)	same	—	activation_27
	average_pooling2d_2	(AveragePooling2D)	(None, 5, 411, 312)	0	(2, 1)	(2, 1)	valid	—	conv2d_27
	batch_normalization_28	(BatchNormalization)	(None, 5, 411, 312)	1248	—	—	—	—	average_pooling2d_2
	activation_28	(Activation)	(None, 5, 411, 312)	0	—	—	—	relu	batch_normalization_28
28	conv2d_28	(Conv2D)	(None, 5, 411, 12)	11232	(3, 1)	(3, 1)	same	—	activation_28
	concatenate_25	(Concatenate)	(None, 5, 411, 324)	0	—	—	—	—	average_pooling2d_2<br>conv2d_28
	batch_normalization_29	(BatchNormalization)	(None, 5, 411, 324)	1296	—	—	—	—	concatenate_25
	activation_29	(Activation)	(None, 5, 411, 324)	0	—	—	—	relu	batch_normalization_29
29	conv2d_29	(Conv2D)	(None, 5, 411, 12)	11664	(3, 1)	(3, 1)	same	—	activation_29
	concatenate_26	(Concatenate)	(None, 5, 411, 336)	0	—	—	—	—	concatenate_25<br>conv2d_29

	batch_normalization_30	(BatchNormalization)	(None, 5, 411, 336)	1344	—	—	—	—	concatenate_26
	activation_30	(Activation)	(None, 5, 411, 336)	0	—	—	—	relu	batch_normalization_30
30	conv2d_30	(Conv2D)	(None, 5, 411, 12)	12096	(3, 1)	(3, 1)	same	—	activation_30
	concatenate_27	(Concatenate)	(None, 5, 411, 348)	0	—	—	—	—	concatenate_26<br>conv2d_30
	batch_normalization_31	(BatchNormalization)	(None, 5, 411, 348)	1392	—	—	—	—	concatenate_27
	activation_31	(Activation)	(None, 5, 411, 348)	0	—	—	—	relu	batch_normalization_31
31	conv2d_31	(Conv2D)	(None, 5, 411, 12)	12528	(3, 1)	(3, 1)	same	—	activation_31
	concatenate_28	(Concatenate)	(None, 5, 411, 360)	0	—	—	—	—	concatenate_27<br>conv2d_31
	batch_normalization_32	(BatchNormalization)	(None, 5, 411, 360)	1440	—	—	—	—	concatenate_28
	activation_32	(Activation)	(None, 5, 411, 360)	0	—	—	—	relu	batch_normalization_32
32	conv2d_32	(Conv2D)	(None, 5, 411, 12)	12960	(3, 1)	(3, 1)	same	—	activation_32
	concatenate_29	(Concatenate)	(None, 5, 411, 372)	0	—	—	—	—	concatenate_28<br>conv2d_32
	batch_normalization_33	(BatchNormalization)	(None, 5, 411, 372)	1488	—	—	—	—	concatenate_29
	activation_33	(Activation)	(None, 5, 411, 372)	0	—	—	—	relu	batch_normalization_33
33	conv2d_33	(Conv2D)	(None, 5, 411, 12)	13392	(3, 1)	(3, 1)	same	—	activation_33
	concatenate_30	(Concatenate)	(None, 5, 411, 384)	0	—	—	—	—	concatenate_29<br>conv2d_33
	batch_normalization_34	(BatchNormalization)	(None, 5, 411, 384)	1536	—	—	—	—	concatenate_30
	activation_34	(Activation)	(None, 5, 411, 384)	0	—	—	—	relu	batch_normalization_34
34	conv2d_34	(Conv2D)	(None, 5, 411, 12)	13824	(3, 1)	(3, 1)	same	—	activation_34
	concatenate_31	(Concatenate)	(None, 5, 411, 396)	0	—	—	—	—	concatenate_30<br>conv2d_34
	batch_normalization_35	(BatchNormalization)	(None, 5, 411, 396)	1584	—	—	—	—	concatenate_31
	activation_35	(Activation)	(None, 5, 411, 396)	0	—	—	—	relu	batch_normalization_35
35	conv2d_35	(Conv2D)	(None, 5, 411, 12)	14256	(3, 1)	(3, 1)	same	—	activation_35
	concatenate_32	(Concatenate)	(None, 5, 411, 408)	0	—	—	—	—	concatenate_31<br>conv2d_35
	batch_normalization_36	(BatchNormalization)	(None, 5, 411, 408)	1632	—	—	—	—	concatenate_32
	activation_36	(Activation)	(None, 5, 411, 408)	0	—	—	—	relu	batch_normalization_36
36	conv2d_36	(Conv2D)	(None, 5, 411, 12)	14688	(3, 1)	(3, 1)	same	—	activation_36
	concatenate_33	(Concatenate)	(None, 5, 411, 420)	0	—	—	—	—	concatenate_32<br>conv2d_36
	batch_normalization_37	(BatchNormalization)	(None, 5, 411, 420)	1680	—	—	—	—	concatenate_33
	activation_37	(Activation)	(None, 5, 411, 420)	0	—	—	—	relu	batch_normalization_37
37	conv2d_37	(Conv2D)	(None, 5, 411, 12)	15120	(3, 1)	(3, 1)	same	—	activation_37
	concatenate_34	(Concatenate)	(None, 5, 411, 432)	0	—	—	—	—	concatenate_33<br>conv2d_37
	batch_normalization_38	(BatchNormalization)	(None, 5, 411, 432)	1728	—	—	—	—	concatenate_34
	activation_38	(Activation)	(None, 5, 411, 432)	0	—	—	—	relu	batch_normalization_38
38	conv2d_38	(Conv2D)	(None, 5, 411, 12)	15552	(3, 1)	(3, 1)	same	—	activation_38
	concatenate_35	(Concatenate)	(None, 5, 411, 444)	0	—	—	—	—	concatenate_34<br>conv2d_38
	batch_normalization_39	(BatchNormalization)	(None, 5, 411, 444)	1776	—	—	—	—	concatenate_35
	activation_39	(Activation)	(None, 5, 411, 444)	0	—	—	—	relu	batch_normalization_39
39	conv2d_39	(Conv2D)	(None, 5, 411, 12)	15984	(3, 1)	(3, 1)	same	—	activation_39
	concatenate_36	(Concatenate)	(None, 5, 411, 456)	0	—	—	—	—	concatenate_35<br>conv2d_39
	batch_normalization_40	(BatchNormalization)	(None, 5, 411, 456)	1824	—	—	—	—	concatenate_36
	activation_40	(Activation)	(None, 5, 411, 456)	0	—	—	—	relu	batch_normalization_40
	average_pooling2d_3	(AveragePooling2D)	(None, 1, 411, 456)	0	(5, 1)	(5, 1)	valid	—	activation_40
	flatten_1	(Flatten)	(None, 187416)	0	—	—	—	—	average_pooling2d_3
40	dense_1	(Dense)	(None, 31)	5809927	—	—	—	softmax	flatten_1

**Total parameters:**	**6,276,463**
**Trainable parameters:**	**6,257,695**
**Non-trainable parameters:**	**18768**

S3.9 Table: DenseNet-40-2D network architecture. Network was implemented using Keras with Tensorflow backend and trained for 10 epochs with 32 batch size. The network architectre was adopted from http://github.com/titu1994/DenseNet.

	Layer	(type)	Output Shape	# Param	Filter size	Stride	Padding	Activation	Connected to
	input_1	(InputLayer)	(None, 80, 411, 1)	0	—	—	—	—	—
1	conv2d_1	(Conv2D)	(None, 40, 206, 24)	1176	(7, 7)	(2, 2)	same	—	input_1
	batch_normalization_1	(BatchNormalization)	(None, 40, 206, 24)	96	—	—	—	—	conv2d_1
	activation_1	(Activation)	(None, 40, 206, 24)	0	—	—	—	—	batch_normalization_1
	max_pooling2d_1	(MaxPooling2D)	(None, 20, 103, 24)	0	(3, 3)	(2, 2)	same	—	activation_1
	batch_normalization_2	(BatchNormalization)	(None, 20, 103, 24)	96	—	—	—	—	max_pooling2d_1
	activation_2	(Activation)	(None, 20, 103, 24)	0	—	—	—	relu	batch_normalization_2
2	conv2d_2	(Conv2D)	(None, 20, 103, 12)	2592	(3, 3)	(3, 3)	same	—	activation_2
	concatenate_1	(Concatenate)	(None, 20, 103, 36)	0	—	—	—	—	max_pooling2d_1<br>conv2d_2
	batch_normalization_3	(BatchNormalization)	(None, 20, 103, 36)	144	—	—	—	—	concatenate_1
	activation_3	(Activation)	(None, 20, 103, 36)	0	—	—	—	relu	batch_normalization_3
3	conv2d_3	(Conv2D)	(None, 20, 103, 12)	3888	(3, 3)	(3, 3)	same	—	activation_3
	concatenate_2	(Concatenate)	(None, 20, 103, 48)	0	—	—	—	—	concatenate_1<br>conv2d_3
	batch_normalization_4	(BatchNormalization)	(None, 20, 103, 48)	192	—	—	—	—	concatenate_2
	activation_4	(Activation)	(None, 20, 103, 48)	0	—	—	—	relu	batch_normalization_4
4	conv2d_4	(Conv2D)	(None, 20, 103, 12)	5184	(3, 3)	(3, 3)	same	—	activation_4
	concatenate_3	(Concatenate)	(None, 20, 103, 60)	0	—	—	—	—	concatenate_2<br>conv2d_4
	batch_normalization_5	(BatchNormalization)	(None, 20, 103, 60)	240	—	—	—	—	concatenate_3
	activation_5	(Activation)	(None, 20, 103, 60)	0	—	—	—	relu	batch_normalization_5
5	conv2d_5	(Conv2D)	(None, 20, 103, 12)	6480	(3, 3)	(3, 3)	same	—	activation_5
	concatenate_4	(Concatenate)	(None, 20, 103, 72)	0	—	—	—	—	concatenate_3<br>conv2d_5
	batch_normalization_6	(BatchNormalization)	(None, 20, 103, 72)	288	—	—	—	—	concatenate_4
	activation_6	(Activation)	(None, 20, 103, 72)	0	—	—	—	relu	batch_normalization_6
6	conv2d_6	(Conv2D)	(None, 20, 103, 12)	7776	(3, 3)	(3, 3)	same	—	activation_6
	concatenate_5	(Concatenate)	(None, 20, 103, 84)	0	—	—	—	—	concatenate_4<br>conv2d_6
	batch_normalization_7	(BatchNormalization)	(None, 20, 103, 84)	336	—	—	—	—	concatenate_5
	activation_7	(Activation)	(None, 20, 103, 84)	0	—	—	—	relu	batch_normalization_7
7	conv2d_7	(Conv2D)	(None, 20, 103, 12)	9072	(3, 3)	(3, 3)	same	—	activation_7
	concatenate_6	(Concatenate)	(None, 20, 103, 96)	0	—	—	—	—	concatenate_5<br>conv2d_7
	batch_normalization_8	(BatchNormalization)	(None, 20, 103, 96)	384	—	—	—	—	concatenate_6
	activation_8	(Activation)	(None, 20, 103, 96)	0	—	—	—	relu	batch_normalization_8
8	conv2d_8	(Conv2D)	(None, 20, 103, 12)	10368	(3, 3)	(3, 3)	same	—	activation_8
	concatenate_7	(Concatenate)	(None, 20, 103, 108)	0	—	—	—	—	concatenate_6<br>conv2d_8
	batch_normalization_9	(BatchNormalization)	(None, 20, 103, 108)	432	—	—	—	—	concatenate_7
	activation_9	(Activation)	(None, 20, 103, 108)	0	—	—	—	relu	batch_normalization_9
9	conv2d_9	(Conv2D)	(None, 20, 103, 12)	11664	(3, 3)	(3, 3)	same	—	activation_9
	concatenate_8	(Concatenate)	(None, 20, 103, 120)	0	—	—	—	—	concatenate_7<br>conv2d_9
	batch_normalization_10	(BatchNormalization)	(None, 20, 103, 120)	480	—	—	—	—	concatenate_8
	activation_10	(Activation)	(None, 20, 103, 120)	0	—	—	—	relu	batch_normalization_10
10	conv2d_10	(Conv2D)	(None, 20, 103, 12)	12960	(3, 3)	(3, 3)	same	—	activation_10
	concatenate_9	(Concatenate)	(None, 20, 103, 132)	0	—	—	—	—	concatenate_8<br>conv2d_10
	batch_normalization_11	(BatchNormalization)	(None, 20, 103, 132)	528	—	—	—	—	concatenate_9
	activation_11	(Activation)	(None, 20, 103, 132)	0	—	—	—	relu	batch_normalization_11
11	conv2d_11	(Conv2D)	(None, 20, 103, 12)	14256	(3, 3)	(3, 3)	same	—	activation_11
	concatenate_10	(Concatenate)	(None, 20, 103, 144)	0	—	—	—	—	concatenate_9<br>conv2d_11
	batch_normalization_12	(BatchNormalization)	(None, 20, 103, 144)	576	—	—	—	—	concatenate_10
	activation_12	(Activation)	(None, 20, 103, 144)	0	—	—	—	relu	batch_normalization_12
12	conv2d_12	(Conv2D)	(None, 20, 103, 12)	15552	(3, 3)	(3, 3)	same	—	activation_12
	concatenate_11	(Concatenate)	(None, 20, 103, 156)	0	—	—	—	—	concatenate_10<br>conv2d_12
	batch_normalization_13	(BatchNormalization)	(None, 20, 103, 156)	624	—	—	—	—	concatenate_11
	activation_13	(Activation)	(None, 20, 103, 156)	0	—	—	—	relu	batch_normalization_13
13	conv2d_13	(Conv2D)	(None, 20, 103, 12)	16848	(3, 3)	(3, 3)	same	—	activation_13
	concatenate_12	(Concatenate)	(None, 20, 103, 168)	0	—	—	—	—	concatenate_11<br>conv2d_13
	batch_normalization_14	(BatchNormalization)	(None, 20, 103, 168)	672	—	—	—	—	concatenate_12
	activation_14	(Activation)	(None, 20, 103, 168)	0	—	—	—	relu	batch_normalization_14
14	conv2d_14	(Conv2D)	(None, 20, 103, 168)	28224	(1, 1)	(1, 1)	same	—	activation_14
	average_pooling2d_1	(AveragePooling2D)	(None, 10, 51, 168)	0	(2, 2)	(2, 2)	valid	—	conv2d_14
	batch_normalization_15	(BatchNormalization)	(None, 10, 51, 168)	672	—	—	—	—	average_pooling2d_1
	activation_15	(Activation)	(None, 10, 51, 168)	0	—	—	—	relu	batch_normalization_15

15 conv2d_15	(Conv2D)	(None, 10, 51, 12)	18144	(3, 3)	(3, 3)	same	—	activation_15
concatenate_13	(Concatenate)	(None, 10, 51, 180)	0	—	—	—	—	average_pooling2d_1<br>conv2d_15
batch_normalization_16	(BatchNormalization)	(None, 10, 51, 180)	720	—	—	—	—	concatenate_13
activation_16	(Activation)	(None, 10, 51, 180)	0	—	—	—	relu	batch_normalization_16
16 conv2d_16	(Conv2D)	(None, 10, 51, 12)	19440	(3, 3)	(3, 3)	same	—	activation_16
concatenate_14	(Concatenate)	(None, 10, 51, 192)	0	—	—	—	—	concatenate_13<br>conv2d_16
batch_normalization_17	(BatchNormalization)	(None, 10, 51, 192)	768	—	—	—	—	concatenate_14
activation_17	(Activation)	(None, 10, 51, 192)	0	—	—	—	relu	batch_normalization_17
17 conv2d_17	(Conv2D)	(None, 10, 51, 12)	20736	(3, 3)	(3, 3)	same	—	activation_17
concatenate_15	(Concatenate)	(None, 10, 51, 204)	0	—	—	—	—	concatenate_14<br>conv2d_17
batch_normalization_18	(BatchNormalization)	(None, 10, 51, 204)	816	—	—	—	—	concatenate_15
activation_18	(Activation)	(None, 10, 51, 204)	0	—	—	—	relu	batch_normalization_18
18 conv2d_18	(Conv2D)	(None, 10, 51, 12)	22032	(3, 3)	(3, 3)	same	—	activation_18
concatenate_16	(Concatenate)	(None, 10, 51, 216)	0	—	—	—	—	concatenate_15<br>conv2d_18
batch_normalization_19	(BatchNormalization)	(None, 10, 51, 216)	864	—	—	—	—	concatenate_16
activation_19	(Activation)	(None, 10, 51, 216)	0	—	—	—	relu	batch_normalization_19
19 conv2d_19	(Conv2D)	(None, 10, 51, 12)	23328	(3, 3)	(3, 3)	same	—	activation_19
concatenate_17	(Concatenate)	(None, 10, 51, 228)	0	—	—	—	—	concatenate_16<br>conv2d_19
batch_normalization_20	(BatchNormalization)	(None, 10, 51, 228)	912	—	—	—	—	concatenate_17
activation_20	(Activation)	(None, 10, 51, 228)	0	—	—	—	relu	batch_normalization_20
20 conv2d_20	(Conv2D)	(None, 10, 51, 12)	24624	(3, 3)	(3, 3)	same	—	activation_20
concatenate_18	(Concatenate)	(None, 10, 51, 240)	0	—	—	—	—	concatenate_17<br>conv2d_20
batch_normalization_21	(BatchNormalization)	(None, 10, 51, 240)	960	—	—	—	—	concatenate_18
activation_21	(Activation)	(None, 10, 51, 240)	0	—	—	—	relu	batch_normalization_21
21 conv2d_21	(Conv2D)	(None, 10, 51, 12)	25920	(3, 3)	(3, 3)	same	—	activation_21
concatenate_19	(Concatenate)	(None, 10, 51, 252)	0	—	—	—	—	concatenate_18<br>conv2d_21
batch_normalization_22	(BatchNormalization)	(None, 10, 51, 252)	1008	—	—	—	—	concatenate_19
activation_22	(Activation)	(None, 10, 51, 252)	0	—	—	—	relu	batch_normalization_22
22 conv2d_22	(Conv2D)	(None, 10, 51, 12)	27216	(3, 3)	(3, 3)	same	—	activation_22
concatenate_20	(Concatenate)	(None, 10, 51, 264)	0	—	—	—	—	concatenate_19<br>conv2d_22
batch_normalization_23	(BatchNormalization)	(None, 10, 51, 264)	1056	—	—	—	—	concatenate_20
activation_23	(Activation)	(None, 10, 51, 264)	0	—	—	—	relu	batch_normalization_23
23 conv2d_23	(Conv2D)	(None, 10, 51, 12)	28512	(3, 3)	(3, 3)	same	—	activation_23
concatenate_21	(Concatenate)	(None, 10, 51, 276)	0	—	—	—	—	concatenate_20<br>conv2d_23
batch_normalization_24	(BatchNormalization)	(None, 10, 51, 276)	1104	—	—	—	—	concatenate_21
activation_24	(Activation)	(None, 10, 51, 276)	0	—	—	—	relu	batch_normalization_24
24 conv2d_24	(Conv2D)	(None, 10, 51, 12)	29808	(3, 3)	(3, 3)	same	—	activation_24
concatenate_22	(Concatenate)	(None, 10, 51, 288)	0	—	—	—	—	concatenate_21<br>conv2d_24
batch_normalization_25	(BatchNormalization)	(None, 10, 51, 288)	1152	—	—	—	—	concatenate_22
activation_25	(Activation)	(None, 10, 51, 288)	0	—	—	—	relu	batch_normalization_25
25 conv2d_25	(Conv2D)	(None, 10, 51, 12)	31104	(3, 3)	(3, 3)	same	—	activation_25
concatenate_23	(Concatenate)	(None, 10, 51, 300)	0	—	—	—	—	concatenate_22<br>conv2d_25
batch_normalization_26	(BatchNormalization)	(None, 10, 51, 300)	1200	—	—	—	—	concatenate_23
activation_26	(Activation)	(None, 10, 51, 300)	0	—	—	—	relu	batch_normalization_26
26 conv2d_26	(Conv2D)	(None, 10, 51, 12)	32400	(3, 3)	(3, 3)	same	—	activation_26
concatenate_24	(Concatenate)	(None, 10, 51, 312)	0	—	—	—	—	concatenate_23<br>conv2d_26
batch_normalization_27	(BatchNormalization)	(None, 10, 51, 312)	1248	—	—	—	—	concatenate_24
activation_27	(Activation)	(None, 10, 51, 312)	0	—	—	—	relu	batch_normalization_27
27 conv2d_27	(Conv2D)	(None, 10, 51, 312)	97344	(1, 1)	(1, 1)	same	—	activation_27
average_pooling2d_2	(AveragePooling2D)	(None, 5, 25, 312)	0	(2, 2)	(2, 2)	valid	—	conv2d_27
batch_normalization_28	(BatchNormalization)	(None, 5, 25, 312)	1248	—	—	—	—	average_pooling2d_2
activation_28	(Activation)	(None, 5, 25, 312)	0	—	—	—	relu	batch_normalization_28
28 conv2d_28	(Conv2D)	(None, 5, 25, 12)	33696	(3, 3)	(3, 3)	same	—	activation_28
concatenate_25	(Concatenate)	(None, 5, 25, 324)	0	—	—	—	—	average_pooling2d_2<br>conv2d_28
batch_normalization_29	(BatchNormalization)	(None, 5, 25, 324)	1296	—	—	—	—	concatenate_25
activation_29	(Activation)	(None, 5, 25, 324)	0	—	—	—	relu	batch_normalization_29
29 conv2d_29	(Conv2D)	(None, 5, 25, 12)	34992	(3, 3)	(3, 3)	same	—	activation_29
concatenate_26	(Concatenate)	(None, 5, 25, 336)	0	—	—	—	—	concatenate_25<br>conv2d_29

	batch_normalization_30	(BatchNormalization)	(None, 5, 25, 336)	1344	—	—	—	—	concatenate_26
	activation_30	(Activation)	(None, 5, 25, 336)	0	—	—	—	relu	batch_normalization_30
30	conv2d_30	(Conv2D)	(None, 5, 25, 12)	36288	(3, 3)	(3, 3)	same	—	activation_30
	concatenate_27	(Concatenate)	(None, 5, 25, 348)	0	—	—	—	—	concatenate_26<br>conv2d_30
	batch_normalization_31	(BatchNormalization)	(None, 5, 25, 348)	1392	—	—	—	—	concatenate_27
	activation_31	(Activation)	(None, 5, 25, 348)	0	—	—	—	relu	batch_normalization_31
31	conv2d_31	(Conv2D)	(None, 5, 25, 12)	37584	(3, 3)	(3, 3)	same	—	activation_31
	concatenate_28	(Concatenate)	(None, 5, 25, 360)	0	—	—	—	—	concatenate_27<br>conv2d_31
	batch_normalization_32	(BatchNormalization)	(None, 5, 25, 360)	1440	—	—	—	—	concatenate_28
	activation_32	(Activation)	(None, 5, 25, 360)	0	—	—	—	relu	batch_normalization_32
32	conv2d_32	(Conv2D)	(None, 5, 25, 12)	38880	(3, 3)	(3, 3)	same	—	activation_32
	concatenate_29	(Concatenate)	(None, 5, 25, 372)	0	—	—	—	—	concatenate_28<br>conv2d_32
	batch_normalization_33	(BatchNormalization)	(None, 5, 25, 372)	1488	—	—	—	—	concatenate_29
	activation_33	(Activation)	(None, 5, 25, 372)	0	—	—	—	relu	batch_normalization_33
33	conv2d_33	(Conv2D)	(None, 5, 25, 12)	40176	(3, 3)	(3, 3)	same	—	activation_33
	concatenate_30	(Concatenate)	(None, 5, 25, 384)	0	—	—	—	—	concatenate_29<br>conv2d_33
	batch_normalization_34	(BatchNormalization)	(None, 5, 25, 384)	1536	—	—	—	—	concatenate_30
	activation_34	(Activation)	(None, 5, 25, 384)	0	—	—	—	relu	batch_normalization_34
34	conv2d_34	(Conv2D)	(None, 5, 25, 12)	41472	(3, 3)	(3, 3)	same	—	activation_34
	concatenate_31	(Concatenate)	(None, 5, 25, 396)	0	—	—	—	—	concatenate_30<br>conv2d_34
	batch_normalization_35	(BatchNormalization)	(None, 5, 25, 396)	1584	—	—	—	—	concatenate_31
	activation_35	(Activation)	(None, 5, 25, 396)	0	—	—	—	relu	batch_normalization_35
35	conv2d_35	(Conv2D)	(None, 5, 25, 12)	42768	(3, 3)	(3, 3)	same	—	activation_35
	concatenate_32	(Concatenate)	(None, 5, 25, 408)	0	—	—	—	—	concatenate_31<br>conv2d_35
	batch_normalization_36	(BatchNormalization)	(None, 5, 25, 408)	1632	—	—	—	—	concatenate_32
	activation_36	(Activation)	(None, 5, 25, 408)	0	—	—	—	relu	batch_normalization_36
36	conv2d_36	(Conv2D)	(None, 5, 25, 12)	44064	(3, 3)	(3, 3)	same	—	activation_36
	concatenate_33	(Concatenate)	(None, 5, 25, 420)	0	—	—	—	—	concatenate_32<br>conv2d_36
	batch_normalization_37	(BatchNormalization)	(None, 5, 25, 420)	1680	—	—	—	—	concatenate_33
	activation_37	(Activation)	(None, 5, 25, 420)	0	—	—	—	relu	batch_normalization_37
37	conv2d_37	(Conv2D)	(None, 5, 25, 12)	45360	(3, 3)	(3, 3)	same	—	activation_37
	concatenate_34	(Concatenate)	(None, 5, 25, 432)	0	—	—	—	—	concatenate_33<br>conv2d_37
	batch_normalization_38	(BatchNormalization)	(None, 5, 25, 432)	1728	—	—	—	—	concatenate_34
	activation_38	(Activation)	(None, 5, 25, 432)	0	—	—	—	relu	batch_normalization_38
38	conv2d_38	(Conv2D)	(None, 5, 25, 12)	46656	(3, 3)	(3, 3)	same	—	activation_38
	concatenate_35	(Concatenate)	(None, 5, 25, 444)	0	—	—	—	—	concatenate_34<br>conv2d_38
	batch_normalization_39	(BatchNormalization)	(None, 5, 25, 444)	1776	—	—	—	—	concatenate_35
	activation_39	(Activation)	(None, 5, 25, 444)	0	—	—	—	relu	batch_normalization_39
39	conv2d_39	(Conv2D)	(None, 5, 25, 12)	47952	(3, 3)	(3, 3)	same	—	activation_39
	concatenate_36	(Concatenate)	(None, 5, 25, 456)	0	—	—	—	—	concatenate_35<br>conv2d_39
	batch_normalization_40	(BatchNormalization)	(None, 5, 25, 456)	1824	—	—	—	—	concatenate_36
	activation_40	(Activation)	(None, 5, 25, 456)	0	—	—	—	relu	batch_normalization_40
	global_average_pooling2d_1	(GlobalAveragePooling2D)	(None, 456)	0	(5, 5)	(5, 5)	valid	—	activation_40
40	dense_1	(Dense)	(None, 31)	14167	—	—	—	softmax	global_average_pooling2d_1

**Total parameters:**	**1,088,239**
**Trainable parameters:**	**1,069,471**
**Non-trainable parameters:**	**18768**

S3.10 Table: ResNet-34-1D network architecture. Network was implemented using Keras with Tensorflow backend and trained for 10 epochs with 32 batch size. The network architectre was adopted from https://github.com/keras-team/keras-contrib/blob/master/keras_contrib/applications/resnet.py

	Layer	(type)	Output Shape	# Param	Filter size	Stride	Padding	Activation	Connected to
	input_1	(InputLayer)	(None, 80, 411, 1)	0	—	—	—	—	—
1	conv2d_1	(Conv2D)	(None, 40, 411, 64)	512	(7, 1)	(2, 1)	same	—	input_1
	batch_normalization_1	(BatchNormalization)	(None, 40, 411, 64)	256	—	—	—	—	conv2d_1
	activation_1	(Activation)	(None, 40, 411, 64)	0	—	—	—	relu	batch_normalization_1
	max_pooling2d_1	(MaxPooling2D)	(None, 20, 411, 64)	0	(3, 1)	(2, 1)	same	—	activation_1
2	res0a_branch2a	(Conv2D)	(None, 20, 411, 64)	12352	(3, 1)	(1, 1)	same	—	max_pooling2d_1
	batch_normalization_2	(BatchNormalization)	(None, 20, 411, 64)	256	—	—	—	—	res0a_branch2a
	activation_2	(Activation)	(None, 20, 411, 64)	0	—	—	—	relu	batch_normalization_2
3	conv2d_2	(Conv2D)	(None, 20, 411, 64)	12352	(3, 1)	(1, 1)	same	—	activation_2
	add_1	(Add)	(None, 20, 411, 64)	0	—	—	—	—	max_pooling2d_1<br>conv2d_2
	batch_normalization_3	(BatchNormalization)	(None, 20, 411, 64)	256	—	—	—	—	add_1
	activation_3	(Activation)	(None, 20, 411, 64)	0	—	—	—	relu	batch_normalization_3
4	conv2d_3	(Conv2D)	(None, 20, 411, 64)	12352	(3, 1)	(1, 1)	same	—	activation_3
	batch_normalization_4	(BatchNormalization)	(None, 20, 411, 64)	256	—	—	—	—	conv2d_3
	activation_4	(Activation)	(None, 20, 411, 64)	0	—	—	—	relu	batch_normalization_4
5	conv2d_4	(Conv2D)	(None, 20, 411, 64)	12352	(3, 1)	(1, 1)	same	—	activation_4
	add_2	(Add)	(None, 20, 411, 64)	0	—	—	—	—	add_1<br>conv2d_4
	batch_normalization_5	(BatchNormalization)	(None, 20, 411, 64)	256	—	—	—	—	add_2
	activation_5	(Activation)	(None, 20, 411, 64)	0	—	—	—	relu	batch_normalization_5
6	conv2d_5	(Conv2D)	(None, 20, 411, 64)	12352	(3, 1)	(1, 1)	same	—	activation_5
	batch_normalization_6	(BatchNormalization)	(None, 20, 411, 64)	256	—	—	—	—	conv2d_5
	activation_6	(Activation)	(None, 20, 411, 64)	0	—	—	—	relu	batch_normalization_6
7	conv2d_6	(Conv2D)	(None, 20, 411, 64)	12352	(3, 1)	(1, 1)	same	—	activation_6
	add_3	(Add)	(None, 20, 411, 64)	0	—	—	—	—	add_2<br>conv2d_6
	batch_normalization_7	(BatchNormalization)	(None, 20, 411, 64)	256	—	—	—	—	add_3
	activation_7	(Activation)	(None, 20, 411, 64)	0	—	—	—	relu	batch_normalization_7
8	conv2d_7	(Conv2D)	(None, 10, 411, 128)	24704	(3, 1)	(2, 1)	same	—	activation_7
	batch_normalization_8	(BatchNormalization)	(None, 10, 411, 128)	512	—	—	—	—	conv2d_7
	conv2d_9	(Conv2D)	(None, 10, 411, 128)	8320	(1, 1)		valid	—	add_3
	activation_8	(Activation)	(None, 10, 411, 128)	0	—	—	—	relu	batch_normalization_8
	batch_normalization_9	(BatchNormalization)	(None, 10, 411, 128)	512	—	—	—	—	conv2d_9
9	conv2d_8	(Conv2D)	(None, 10, 411, 128)	49280	(3, 1)	(1, 1)	same	—	activation_8
	add_4	(Add)	(None, 10, 411, 128)	0	—	—	—	—	batch_normalization_9<br>conv2d_8
	batch_normalization_10	(BatchNormalization)	(None, 10, 411, 128)	512	—	—	—	—	add_4
	activation_9	(Activation)	(None, 10, 411, 128)	0	—	—	—	relu	batch_normalization_10
10	conv2d_10	(Conv2D)	(None, 10, 411, 128)	49280	(3, 1)	(1, 1)	same	—	activation_9
	batch_normalization_11	(BatchNormalization)	(None, 10, 411, 128)	512	—	—	—	—	conv2d_10
	activation_10	(Activation)	(None, 10, 411, 128)	0	—	—	—	relu	batch_normalization_11
11	conv2d_11	(Conv2D)	(None, 10, 411, 128)	49280	(3, 1)	(1, 1)	same	—	activation_10
	add_5	(Add)	(None, 10, 411, 128)	0	—	—	—	—	add_4<br>conv2d_11
	batch_normalization_12	(BatchNormalization)	(None, 10, 411, 128)	512	—	—	—	—	add_5
	activation_11	(Activation)	(None, 10, 411, 128)	0	—	—	—	relu	batch_normalization_12
12	conv2d_12	(Conv2D)	(None, 10, 411, 128)	49280	(3, 1)	(1, 1)	same	—	activation_11
	batch_normalization_13	(BatchNormalization)	(None, 10, 411, 128)	512	—	—	—	—	conv2d_12
	activation_12	(Activation)	(None, 10, 411, 128)	0	—	—	—	relu	batch_normalization_13
13	conv2d_13	(Conv2D)	(None, 10, 411, 128)	49280	(3, 1)	(1, 1)	same	—	activation_12
	add_6	(Add)	(None, 10, 411, 128)	0	—	—	—	—	add_5<br>conv2d_13
	batch_normalization_14	(BatchNormalization)	(None, 10, 411, 128)	512	—	—	—	—	add_6
	activation_13	(Activation)	(None, 10, 411, 128)	0	—	—	—	relu	batch_normalization_14
14	conv2d_14	(Conv2D)	(None, 10, 411, 128)	49280	(3, 1)	(1, 1)	same	—	activation_13
	batch_normalization_15	(BatchNormalization)	(None, 10, 411, 128)	512	—	—	—	—	conv2d_14
	activation_14	(Activation)	(None, 10, 411, 128)	0	—	—	—	relu	batch_normalization_15
15	conv2d_15	(Conv2D)	(None, 10, 411, 128)	49280	(3, 1)	(1, 1)	same	—	activation_14
	add_7	(Add)	(None, 10, 411, 128)	0	—	—	—	—	add_6<br>conv2d_15
	batch_normalization_16	(BatchNormalization)	(None, 10, 411, 128)	512	—	—	—	—	add_7
	activation_15	(Activation)	(None, 10, 411, 128)	0	—	—	—	relu	batch_normalization_16
16	conv2d_16	(Conv2D)	(None, 5, 411, 256)	98560	(3, 1)	(2, 1)	same	—	activation_15
	batch_normalization_17	(BatchNormalization)	(None, 5, 411, 256)	1024	—	—	—	—	conv2d_16
	conv2d_18	(Conv2D)	(None, 5, 411, 256)	33024	(1, 1)		valid	—	add_7
	activation_16	(Activation)	(None, 5, 411, 256)	0	—	—	—	relu	batch_normalization_17
	batch_normalization_18	(BatchNormalization)	(None, 5, 411, 256)	1024	—	—	—	—	conv2d_18
17	conv2d_17	(Conv2D)	(None, 5, 411, 256)	196864	(3, 1)	(1, 1)	same	—	activation_16
	add_8	(Add)	(None, 5, 411, 256)	0	—	—	—	—	batch_normalization_18<br>conv2d_17

	batch_normalization_19	(BatchNormalization)	(None, 5, 411, 256)	1024	—	—	—	—	add_8
	activation_17	(Activation)	(None, 5, 411, 256)	0	—	—	—	relu	batch_normalization_19
18	conv2d_19	(Conv2D)	(None, 5, 411, 256)	196864	(3, 1)	(1, 1)	same	—	activation_17
	batch_normalization_20	(BatchNormalization)	(None, 5, 411, 256)	1024	—	—	—	—	conv2d_19
	activation_18	(Activation)	(None, 5, 411, 256)	0	—	—	—	relu	batch_normalization_20
19	conv2d_20	(Conv2D)	(None, 5, 411, 256)	196864	(3, 1)	(1, 1)	same	—	activation_18
	add_9	(Add)	(None, 5, 411, 256)	0	—	—	—	—	add_8<br>conv2d_20
	batch_normalization_21	(BatchNormalization)	(None, 5, 411, 256)	1024	—	—	—	—	add_9
	activation_19	(Activation)	(None, 5, 411, 256)	0	—	—	—	relu	batch_normalization_21
20	conv2d_21	(Conv2D)	(None, 5, 411, 256)	196864	(3, 1)	(1, 1)	same	—	activation_19
	batch_normalization_22	(BatchNormalization)	(None, 5, 411, 256)	1024	—	—	—	—	conv2d_21
	activation_20	(Activation)	(None, 5, 411, 256)	0	—	—	—	relu	batch_normalization_22
21	conv2d_22	(Conv2D)	(None, 5, 411, 256)	196864	(3, 1)	(1, 1)	same	—	activation_20
	add_10	(Add)	(None, 5, 411, 256)	0	—	—	—	—	add_9<br>conv2d_22
	batch_normalization_23	(BatchNormalization)	(None, 5, 411, 256)	1024	—	—	—	—	add_10
	activation_21	(Activation)	(None, 5, 411, 256)	0	—	—	—	relu	batch_normalization_23
22	conv2d_23	(Conv2D)	(None, 5, 411, 256)	196864	(3, 1)	(1, 1)	same	—	activation_21
	batch_normalization_24	(BatchNormalization)	(None, 5, 411, 256)	1024	—	—	—	—	conv2d_23
	activation_22	(Activation)	(None, 5, 411, 256)	0	—	—	—	relu	batch_normalization_24
23	conv2d_24	(Conv2D)	(None, 5, 411, 256)	196864	(3, 1)	(1, 1)	same	—	activation_22
	add_11	(Add)	(None, 5, 411, 256)	0	—	—	—	—	add_10<br>conv2d_24
	batch_normalization_25	(BatchNormalization)	(None, 5, 411, 256)	1024	—	—	—	—	add_11
	activation_23	(Activation)	(None, 5, 411, 256)	0	—	—	—	relu	batch_normalization_25
24	conv2d_25	(Conv2D)	(None, 5, 411, 256)	196864	(3, 1)	(1, 1)	same	—	activation_23
	batch_normalization_26	(BatchNormalization)	(None, 5, 411, 256)	1024	—	—	—	—	conv2d_25
	activation_24	(Activation)	(None, 5, 411, 256)	0	—	—	—	relu	batch_normalization_26
25	conv2d_26	(Conv2D)	(None, 5, 411, 256)	196864	(3, 1)	(1, 1)	same	—	activation_24
	add_12	(Add)	(None, 5, 411, 256)	0	—	—	—	—	add_11<br>conv2d_26
	batch_normalization_27	(BatchNormalization)	(None, 5, 411, 256)	1024	—	—	—	—	add_12
	activation_25	(Activation)	(None, 5, 411, 256)	0	—	—	—	relu	batch_normalization_27
26	conv2d_27	(Conv2D)	(None, 5, 411, 256)	196864	(3, 1)	(1, 1)	same	—	activation_25
	batch_normalization_28	(BatchNormalization)	(None, 5, 411, 256)	1024	—	—	—	—	conv2d_27
	activation_26	(Activation)	(None, 5, 411, 256)	0	—	—	—	relu	batch_normalization_28
27	conv2d_28	(Conv2D)	(None, 5, 411, 256)	196864	(3, 1)	(1, 1)	same	—	activation_26
	add_13	(Add)	(None, 5, 411, 256)	0	—	—	—	—	add_12<br>conv2d_28
	batch_normalization_29	(BatchNormalization)	(None, 5, 411, 256)	1024	—	—	—	—	add_13
	activation_27	(Activation)	(None, 5, 411, 256)	0	—	—	—	relu	batch_normalization_29
28	conv2d_29	(Conv2D)	(None, 3, 411, 512)	393728	(3, 1)	(2, 1)	same	—	activation_27
	batch_normalization_30	(BatchNormalization)	(None, 3, 411, 512)	2048	—	—	—	—	conv2d_29
	conv2d_31	(Conv2D)	(None, 3, 411, 512)	131584	(1, 1)		valid	—	add_13
	activation_28	(Activation)	(None, 3, 411, 512)	0	—	—	—	relu	batch_normalization_30
	batch_normalization_31	(BatchNormalization)	(None, 3, 411, 512)	2048	—	—	—	—	conv2d_31
29	conv2d_30	(Conv2D)	(None, 3, 411, 512)	786944	(3, 1)	(1, 1)	same	—	activation_28
	add_14	(Add)	(None, 3, 411, 512)	0	—	—	—	—	batch_normalization_31<br>conv2d_30
	batch_normalization_32	(BatchNormalization)	(None, 3, 411, 512)	2048	—	—	—	—	add_14
	activation_29	(Activation)	(None, 3, 411, 512)	0	—	—	—	relu	batch_normalization_32
30	conv2d_32	(Conv2D)	(None, 3, 411, 512)	786944	(3, 1)	(1, 1)	same	—	activation_29
	batch_normalization_33	(BatchNormalization)	(None, 3, 411, 512)	2048	—	—	—	—	conv2d_32
	activation_30	(Activation)	(None, 3, 411, 512)	0	—	—	—	relu	batch_normalization_33
31	conv2d_33	(Conv2D)	(None, 3, 411, 512)	786944	(3, 1)	(1, 1)	same	—	activation_30
	add_15	(Add)	(None, 3, 411, 512)	0	—	—	—	—	add_14<br>conv2d_33
	batch_normalization_34	(BatchNormalization)	(None, 3, 411, 512)	2048	—	—	—	—	add_15
	activation_31	(Activation)	(None, 3, 411, 512)	0	—	—	—	relu	batch_normalization_34
32	conv2d_34	(Conv2D)	(None, 3, 411, 512)	786944	(3, 1)	(1, 1)	same	—	activation_31
	batch_normalization_35	(BatchNormalization)	(None, 3, 411, 512)	2048	—	—	—	—	conv2d_34
	activation_32	(Activation)	(None, 3, 411, 512)	0	—	—	—	relu	batch_normalization_35
33	conv2d_35	(Conv2D)	(None, 3, 411, 512)	786944	(3, 1)	(1, 1)	same	—	activation_32
	add_16	(Add)	(None, 3, 411, 512)	0	—	—	—	—	add_15<br>conv2d_35
	batch_normalization_36	(BatchNormalization)	(None, 3, 411, 512)	2048	—	—	—	—	add_16
	activation_33	(Activation)	(None, 3, 411, 512)	0	—	—	—	relu	batch_normalization_36
	average_pooling2d_1	(AveragePooling2D)	(None, 1, 411, 512)	0	—	—	—	—	activation_33
	flatten_1	(Flatten)	(None, 210432)	0	—	—	—	—	average_pooling2d_1
34	dense_1	(Dense)	(None, 31)	6523423	—	—	—	softmax	flatten_1

**Total parameters:**	**13,767,199**
**Trainable parameters:**	**13,750,175**
**Non-trainable parameters:**	**17024**

S3.11 Table: ResNet-34-2D network architecture. Network was implemented using Keras with Tensorflow backend and trained for 10 epochs with 32 batch size. The network architectre was adopted from https://github.com/keras-team/keras-contrib/blob/master/keras_contrib/applications/resnet.py

	Layer	(type)	Output Shape	# Param	Filter size	Stride	Padding	Activation	Connected to
	input_1	(InputLayer)	(None, 80, 411, 1)	0	—	—	—	—	—
1	conv2d_1	(Conv2D)	(None, 40, 206, 64)	3200	(7, 7)	(2, 2)	same	—	input_1
	batch_normalization_1	(BatchNormalization)	(None, 40, 206, 64)	256	—	—	—	—	conv2d_1
	activation_1	(Activation)	(None, 40, 206, 64)	0	—	—	—	relu	batch_normalization_1
	max_pooling2d_1	(MaxPooling2D)	(None, 20, 103, 64)	0	(3, 3)	(2, 2)	same	—	activation_1
2	res0a_branch2a	(Conv2D)	(None, 20, 103, 64)	36928	(3, 3)	(1, 1)	same	—	max_pooling2d_1
	batch_normalization_2	(BatchNormalization)	(None, 20, 103, 64)	256	—	—	—	—	res0a_branch2a
	activation_2	(Activation)	(None, 20, 103, 64)	0	—	—	—	relu	batch_normalization_2
3	conv2d_2	(Conv2D)	(None, 20, 103, 64)	36928	(3, 3)	(1, 1)	same	—	activation_2
	add_1	(Add)	(None, 20, 103, 64)	0	—	—	—	—	max_pooling2d_1<br>conv2d_2
	batch_normalization_3	(BatchNormalization)	(None, 20, 103, 64)	256	—	—	—	—	add_1
	activation_3	(Activation)	(None, 20, 103, 64)	0	—	—	—	relu	batch_normalization_3
4	conv2d_3	(Conv2D)	(None, 20, 103, 64)	36928	(3, 3)	(1, 1)	same	—	activation_3
	batch_normalization_4	(BatchNormalization)	(None, 20, 103, 64)	256	—	—	—	—	conv2d_3
	activation_4	(Activation)	(None, 20, 103, 64)	0	—	—	—	relu	batch_normalization_4
5	conv2d_4	(Conv2D)	(None, 20, 103, 64)	36928	(3, 3)	(1, 1)	same	—	activation_4
	add_2	(Add)	(None, 20, 103, 64)	0	—	—	—	—	add_1<br>conv2d_4
	batch_normalization_5	(BatchNormalization)	(None, 20, 103, 64)	256	—	—	—	—	add_2
	activation_5	(Activation)	(None, 20, 103, 64)	0	—	—	—	relu	batch_normalization_5
6	conv2d_5	(Conv2D)	(None, 20, 103, 64)	36928	(3, 3)	(1, 1)	same	—	activation_5
	batch_normalization_6	(BatchNormalization)	(None, 20, 103, 64)	256	—	—	—	—	conv2d_5
	activation_6	(Activation)	(None, 20, 103, 64)	0	—	—	—	relu	batch_normalization_6
7	conv2d_6	(Conv2D)	(None, 20, 103, 64)	36928	(3, 3)	(1, 1)	same	—	activation_6
	add_3	(Add)	(None, 20, 103, 64)	0	—	—	—	—	add_2<br>conv2d_6
	batch_normalization_7	(BatchNormalization)	(None, 20, 103, 64)	256	—	—	—	—	add_3
	activation_7	(Activation)	(None, 20, 103, 64)	0	—	—	—	relu	batch_normalization_7
8	conv2d_7	(Conv2D)	(None, 10, 52, 128)	73856	(3, 3)	(1, 1)	same	—	activation_7
	batch_normalization_8	(BatchNormalization)	(None, 10, 52, 128)	512	—	—	—	—	conv2d_7
	conv2d_9	(Conv2D)	(None, 10, 52, 128)	8320	(1, 1)		valid	—	add_3
	activation_8	(Activation)	(None, 10, 52, 128)	0	—	—	—	relu	batch_normalization_8
	batch_normalization_9	(BatchNormalization)	(None, 10, 52, 128)	512	—	—	—	—	conv2d_9
9	conv2d_8	(Conv2D)	(None, 10, 52, 128)	147584	(3, 3)	(1, 1)	same	—	activation_8
	add_4	(Add)	(None, 10, 52, 128)	0	—	—	—	—	batch_normalization_9<br>conv2d_8
	batch_normalization_10	(BatchNormalization)	(None, 10, 52, 128)	512	—	—	—	—	add_4
	activation_9	(Activation)	(None, 10, 52, 128)	0	—	—	—	relu	batch_normalization_10
10	conv2d_10	(Conv2D)	(None, 10, 52, 128)	147584	(3, 3)	(1, 1)	same	—	activation_9
	batch_normalization_11	(BatchNormalization)	(None, 10, 52, 128)	512	—	—	—	—	conv2d_10
	activation_10	(Activation)	(None, 10, 52, 128)	0	—	—	—	relu	batch_normalization_11
11	conv2d_11	(Conv2D)	(None, 10, 52, 128)	147584	(3, 3)	(1, 1)	same	—	activation_10
	add_5	(Add)	(None, 10, 52, 128)	0	—	—	—	—	add_4<br>conv2d_11
	batch_normalization_12	(BatchNormalization)	(None, 10, 52, 128)	512	—	—	—	—	add_5
	activation_11	(Activation)	(None, 10, 52, 128)	0	—	—	—	relu	batch_normalization_12
12	conv2d_12	(Conv2D)	(None, 10, 52, 128)	147584	(3, 3)	(1, 1)	same	—	activation_11
	batch_normalization_13	(BatchNormalization)	(None, 10, 52, 128)	512	—	—	—	—	conv2d_12
	activation_12	(Activation)	(None, 10, 52, 128)	0	—	—	—	relu	batch_normalization_13
13	conv2d_13	(Conv2D)	(None, 10, 52, 128)	147584	(3, 3)	(1, 1)	same	—	activation_12
	add_6	(Add)	(None, 10, 52, 128)	0	—	—	—	—	add_5<br>conv2d_13
	batch_normalization_14	(BatchNormalization)	(None, 10, 52, 128)	512	—	—	—	—	add_6
	activation_13	(Activation)	(None, 10, 52, 128)	0	—	—	—	relu	batch_normalization_14
14	conv2d_14	(Conv2D)	(None, 10, 52, 128)	147584	(3, 3)	(1, 1)	same	—	activation_13
	batch_normalization_15	(BatchNormalization)	(None, 10, 52, 128)	512	—	—	—	—	conv2d_14
	activation_14	(Activation)	(None, 10, 52, 128)	0	—	—	—	relu	batch_normalization_15
15	conv2d_15	(Conv2D)	(None, 10, 52, 128)	147584	(3, 3)	(1, 1)	same	—	activation_14
	add_7	(Add)	(None, 10, 52, 128)	0	—	—	—	—	add_6<br>conv2d_15
	batch_normalization_16	(BatchNormalization)	(None, 10, 52, 128)	512	—	—	—	—	add_7
	activation_15	(Activation)	(None, 10, 52, 128)	0	—	—	—	relu	batch_normalization_16
16	conv2d_16	(Conv2D)	(None, 5, 26, 256)	295168	(3, 3)	(1, 1)	same	—	activation_15
	batch_normalization_17	(BatchNormalization)	(None, 5, 26, 256)	1024	—	—	—	—	conv2d_16
	conv2d_18	(Conv2D)	(None, 5, 26, 256)	33024	(1, 1)		valid	—	add_7
	activation_16	(Activation)	(None, 5, 26, 256)	0	—	—	—	relu	batch_normalization_17
	batch_normalization_18	(BatchNormalization)	(None, 5, 26, 256)	1024	—	—	—	—	conv2d_18
17	conv2d_17	(Conv2D)	(None, 5, 26, 256)	590080	(3, 3)	(1, 1)	same	—	activation_16
	add_8	(Add)	(None, 5, 26, 256)	0	—	—	—	—	batch_normalization_18<br>conv2d_17

	batch_normalization_19	(BatchNormalization)	(None, 5, 26, 256)	1024	—	—	—	—	add_8
	activation_17	(Activation)	(None, 5, 26, 256)	0	—	—	—	relu	batch_normalization_19
18	conv2d_19	(Conv2D)	(None, 5, 26, 256)	590080	(3, 3)	(1, 1)	same	—	activation_17
	batch_normalization_20	(BatchNormalization)	(None, 5, 26, 256)	1024	—	—	—	—	conv2d_19
	activation_18	(Activation)	(None, 5, 26, 256)	0	—	—	—	relu	batch_normalization_20
19	conv2d_20	(Conv2D)	(None, 5, 26, 256)	590080	(3, 3)	(1, 1)	same	—	activation_18
	add_9	(Add)	(None, 5, 26, 256)	0	—	—	—	—	add_8<br>conv2d_20
	batch_normalization_21	(BatchNormalization)	(None, 5, 26, 256)	1024	—	—	—	—	add_9
	activation_19	(Activation)	(None, 5, 26, 256)	0	—	—	—	relu	batch_normalization_21
20	conv2d_21	(Conv2D)	(None, 5, 26, 256)	590080	(3, 3)	(1, 1)	same	—	activation_19
	batch_normalization_22	(BatchNormalization)	(None, 5, 26, 256)	1024	—	—	—	—	conv2d_21
	activation_20	(Activation)	(None, 5, 26, 256)	0	—	—	—	relu	batch_normalization_22
21	conv2d_22	(Conv2D)	(None, 5, 26, 256)	590080	(3, 3)	(1, 1)	same	—	activation_20
	add_10	(Add)	(None, 5, 26, 256)	0	—	—	—	—	add_9<br>conv2d_22
	batch_normalization_23	(BatchNormalization)	(None, 5, 26, 256)	1024	—	—	—	—	add_10
	activation_21	(Activation)	(None, 5, 26, 256)	0	—	—	—	relu	batch_normalization_23
22	conv2d_23	(Conv2D)	(None, 5, 26, 256)	590080	(3, 3)	(1, 1)	same	—	activation_21
	batch_normalization_24	(BatchNormalization)	(None, 5, 26, 256)	1024	—	—	—	—	conv2d_23
	activation_22	(Activation)	(None, 5, 26, 256)	0	—	—	—	relu	batch_normalization_24
23	conv2d_24	(Conv2D)	(None, 5, 26, 256)	590080	(3, 3)	(1, 1)	same	—	activation_22
	add_11	(Add)	(None, 5, 26, 256)	0	—	—	—	—	add_10<br>conv2d_24
	batch_normalization_25	(BatchNormalization)	(None, 5, 26, 256)	1024	—	—	—	—	add_11
	activation_23	(Activation)	(None, 5, 26, 256)	0	—	—	—	relu	batch_normalization_25
24	conv2d_25	(Conv2D)	(None, 5, 26, 256)	590080	(3, 3)	(1, 1)	same	—	activation_23
	batch_normalization_26	(BatchNormalization)	(None, 5, 26, 256)	1024	—	—	—	—	conv2d_25
	activation_24	(Activation)	(None, 5, 26, 256)	0	—	—	—	relu	batch_normalization_26
25	conv2d_26	(Conv2D)	(None, 5, 26, 256)	590080	(3, 3)	(1, 1)	same	—	activation_24
	add_12	(Add)	(None, 5, 26, 256)	0	—	—	—	—	add_11<br>conv2d_26
	batch_normalization_27	(BatchNormalization)	(None, 5, 26, 256)	1024	—	—	—	—	add_12
	activation_25	(Activation)	(None, 5, 26, 256)	0	—	—	—	relu	batch_normalization_27
26	conv2d_27	(Conv2D)	(None, 5, 26, 256)	590080	(3, 3)	(1, 1)	same	—	activation_25
	batch_normalization_28	(BatchNormalization)	(None, 5, 26, 256)	1024	—	—	—	—	conv2d_27
	activation_26	(Activation)	(None, 5, 26, 256)	0	—	—	—	relu	batch_normalization_28
27	conv2d_28	(Conv2D)	(None, 5, 26, 256)	590080	(3, 3)	(1, 1)	same	—	activation_26
	add_13	(Add)	(None, 5, 26, 256)	0	—	—	—	—	add_12<br>conv2d_28
	batch_normalization_29	(BatchNormalization)	(None, 5, 26, 256)	1024	—	—	—	—	add_13
	activation_27	(Activation)	(None, 5, 26, 256)	0	—	—	—	relu	batch_normalization_29
28	conv2d_29	(Conv2D)	(None, 3, 13, 512)	1180160	(3, 3)	(1, 1)	same	—	activation_27
	batch_normalization_30	(BatchNormalization)	(None, 3, 13, 512)	2048	—	—	—	—	conv2d_29
	conv2d_31	(Conv2D)	(None, 3, 13, 512)	131584	(1, 1)		valid	—	add_13
	activation_28	(Activation)	(None, 3, 13, 512)	0	—	—	—	relu	batch_normalization_30
	batch_normalization_31	(BatchNormalization)	(None, 3, 13, 512)	2048	—	—	—	—	conv2d_31
29	conv2d_30	(Conv2D)	(None, 3, 13, 512)	2359808	(3, 3)	(1, 1)	same	—	activation_28
	add_14	(Add)	(None, 3, 13, 512)	0	—	—	—	—	batch_normalization_31<br>conv2d_30
	batch_normalization_32	(BatchNormalization)	(None, 3, 13, 512)	2048	—	—	—	—	add_14
	activation_29	(Activation)	(None, 3, 13, 512)	0	—	—	—	relu	batch_normalization_32
30	conv2d_32	(Conv2D)	(None, 3, 13, 512)	2359808	(3, 3)	(1, 1)	same	—	activation_29
	batch_normalization_33	(BatchNormalization)	(None, 3, 13, 512)	2048	—	—	—	—	conv2d_32
	activation_30	(Activation)	(None, 3, 13, 512)	0	—	—	—	relu	batch_normalization_33
31	conv2d_33	(Conv2D)	(None, 3, 13, 512)	2359808	(3, 3)	(1, 1)	same	—	activation_30
	add_15	(Add)	(None, 3, 13, 512)	0	—	—	—	—	add_14<br>conv2d_33
	batch_normalization_34	(BatchNormalization)	(None, 3, 13, 512)	2048	—	—	—	—	add_15
	activation_31	(Activation)	(None, 3, 13, 512)	0	—	—	—	relu	batch_normalization_34
32	conv2d_34	(Conv2D)	(None, 3, 13, 512)	2359808	(3, 3)	(1, 1)	same	—	activation_31
	batch_normalization_35	(BatchNormalization)	(None, 3, 13, 512)	2048	—	—	—	—	conv2d_34
	activation_32	(Activation)	(None, 3, 13, 512)	0	—	—	—	relu	batch_normalization_35
33	conv2d_35	(Conv2D)	(None, 3, 13, 512)	2359808	(3, 3)	(1, 1)	same	—	activation_32
	add_16	(Add)	(None, 3, 13, 512)	0		—	—	—	add_15<br>conv2d_35
	batch_normalization_36	(BatchNormalization)	(None, 3, 13, 512)	2048		—	—	—	add_16
	activation_33	(Activation)	(None, 3, 13, 512)	0		—	—	relu	batch_normalization_36
	global_average_pooling2d_1	(GlogalAveragePooling2D)	(None, 512)	0		—	—	—	activation_33
34	dense_1	(Dense)	(None, 31)	15903		—	—	softmax	global_average_pooling2d_1

**Total parameters:**	**21,319,839**
**Trainable parameters:**	**21,302,815**
**Non-trainable parameters:**	**17024**

4 VGG-8-1D model – Results

S4.1 Table: Results of the testing clinical sample analysis with the CNN-based system with VGG-8-1D model. Results evaluated per each target VOC.

Label - the class label of VOC. *TP* - number of true positives detected per VOC. *TTP* - number of tentative true positives detected per VOC. *FP* - number of false positives detected per VOC. *TTN* - number of tentative true negatives detected per VOC. *FN* - number of false negatives detected per VOC. *TN* - number of true negatives detected per VOC (evaluation excluding *RT* position). *FP** - number of false positives detected per VOC (evaluation excluding *RT* position). *TTP** - number of tentative true positives detected per VOC (evaluation excluding *RT* position). *Sensitivity (expert)* - Sensitivity per VOC in relation to expert-derived ground truth benchmark; tentative true positives (TTP) are considered FP, tentative true negatives (TTN) are considered FN; sensitivity = TP/(TP+FN+TTN). *Sensitivity (system)* - Sensitivity per VOC in relation to system-derived correction benchmark; tentative true positives (TTP) are considered TP, tentative true negatives (TTN) are considered TN; sensitivity=(TP+TTP)/(TP+TTP+FN). *Specificity (expert)* - Specificity per VOC in relation to expert-derived ground truth benchmark; tentative true positives (TTP*) are considered FP*; specificity=TN/TN+FP*+TTP*. *Specificity (system)* - Specificity per VOC in relation to system-derived correction benchmark; tentative true positives (TTP*) are considered TP; specificity=TN/TN+FP*. *AP (expert)* - Average precision per VOC in relation to expert-derived ground truth benchmark. *AP (system)* - Average precision per VOC in relation to system-derived correction benchmark.

Label	TP	TTP	FP	TTN	FN	TN	FP*	TTP*	Sensitivity (expert)	Sensitivity (system)	Specificity (expert)	Specificity (system)	AP (expert)	AP (system)
1	20	18	0	0	0	0	0	18	1	1	0	-	0.8282	1
2	29	7	0	0	0	2	0	7	1	1	0.2222	1	0.9706	1
3	30	8	0	0	0	0	0	8	1	1	0	-	0.9989	1
4	38	0	0	0	0	0	0	0	1	1	-	-	1	1
5	35	3	0	1	0	0	0	2	0.9722	1	0	-	0.9393	1
6	10	27	0	1	0	1	0	26	0.9091	1	0.037	1	0.6951	1
7	23	7	0	0	0	8	0	7	1	1	0.5333	1	0.9917	1
8	37	1	0	0	0	0	0	1	1	1	0	-	0.9915	1
9	38	0	0	0	0	0	0	0	1	1	-	-	1	1
10	29	5	0	1	0	4	0	4	0.9667	1	0.5	1	0.9622	1
11	13	20	0	6	0	5	0	14	0.6842	1	0.2632	1	0.4153	1
12	24	10	0	1	0	4	0	9	0.96	1	0.3077	1	0.8974	1
13	32	4	0	1	0	2	0	3	0.9697	1	0.4	1	0.926	1
14	32	2	0	0	1	3	0	2	0.9697	0.9714	0.6	1	0.9642	0.9714
15	22	15	0	1	0	1	0	14	0.9565	1	0.0667	1	0.8076	1
16	19	11	2	1	3	4	1	10	0.8261	0.9091	0.2667	0.8	0.8086	0.8965
17	38	0	0	0	0	0	0	0	1	1	-	-	1	1
18	32	2	0	0	4	0	0	2	0.8889	0.8947	0	-	0.8676	0.8947
19	26	11	0	0	0	1	0	11	1	1	0.0833	1	0.8144	1
20	36	0	0	0	1	1	0	0	0.973	0.973	1	1	0.973	0.973
21	21	15	0	4	2	0	0	11	0.7778	0.9474	0	-	0.745	0.9474
22	32	6	0	0	0	0	0	6	1	1	0	-	0.9879	1
23	36	0	0	0	0	2	0	0	1	1	1	1	1	1
24	24	13	0	0	0	1	0	13	1	1	0.0714	1	0.9208	1
25	7	5	0	0	0	26	0	5	1	1	0.8387	1	0.8052	1
26	21	16	0	1	0	1	0	15	0.9545	1	0.0625	1	0.9313	1
27	32	4	0	0	0	2	0	4	1	1	0.3333	1	0.9789	1
28	17	6	0	0	0	15	0	6	1	1	0.7143	1	0.8823	1
29	31	7	0	0	0	0	0	7	1	1	0	-	0.957	1
30	32	3	0	0	0	3	0	3	1	1	0.5	1	0.9962	1
**TOTAL**	**816**	**226**	**2**	**18**	**11**	**86**	**1**	**208**	**0.9657**	**0.9896**	**0.2915**	**0.9885**	**mAP**	
													**0.9019**	**0.9894**

S4.1-S4.8 Figures: Precision against recall graphs for VOCs 1-8.

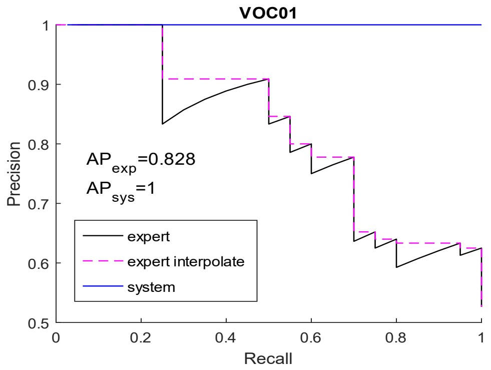

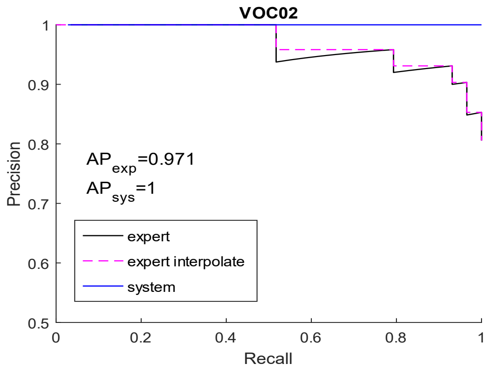

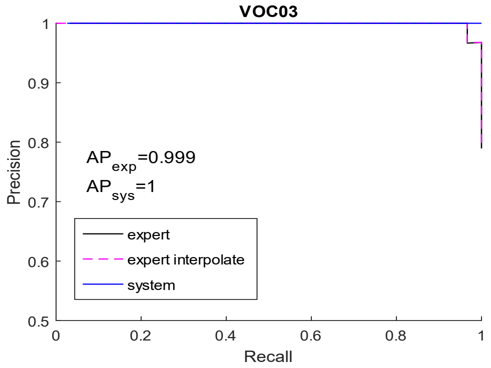

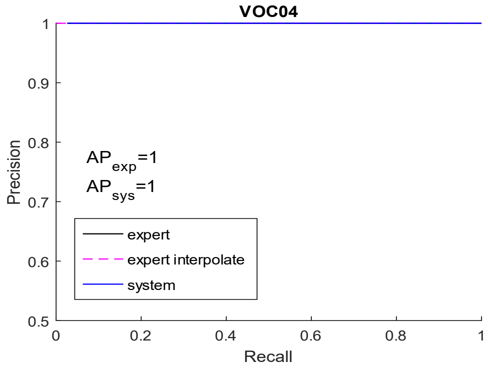

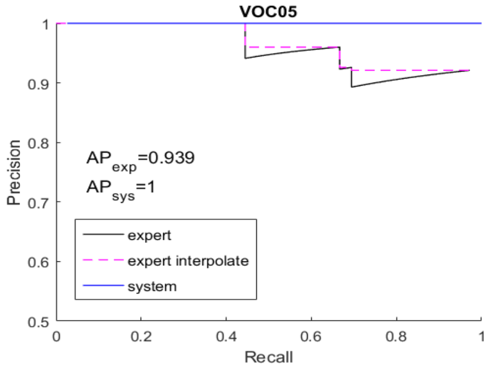

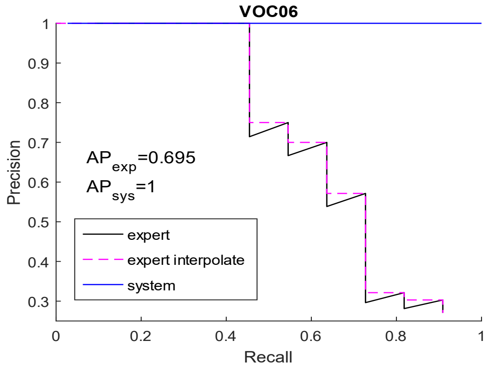

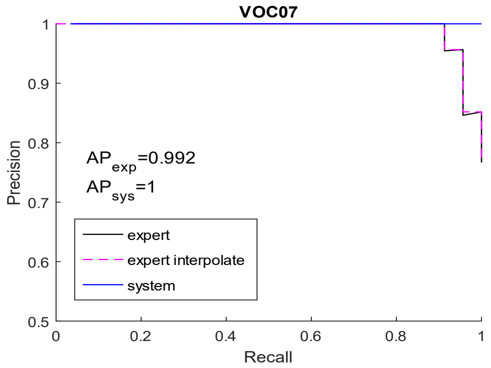

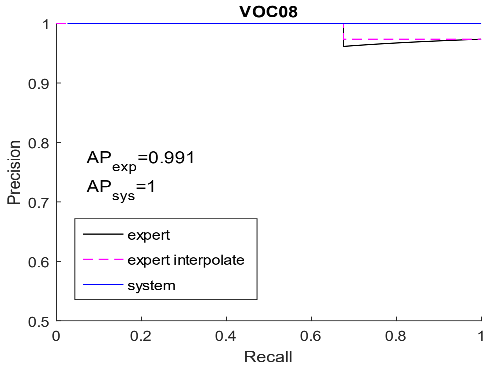

S4.9-S4.16 Figures: Precision against recall graphs for VOCs 9-16.

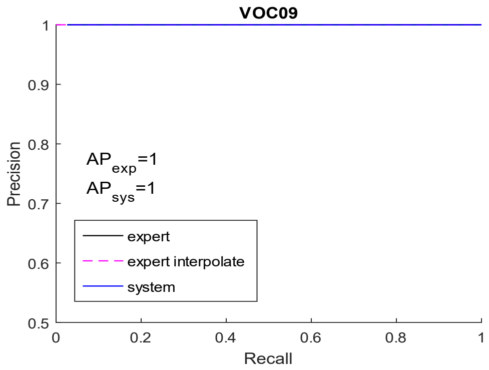

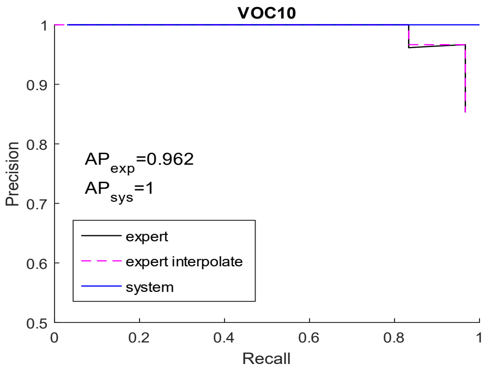

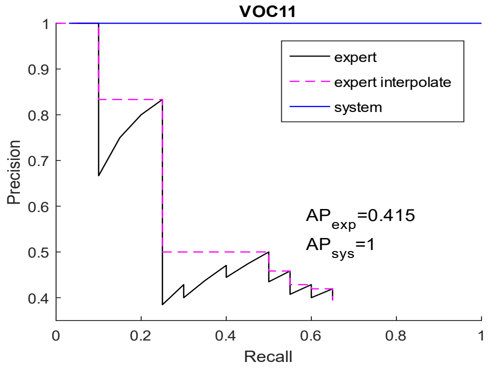

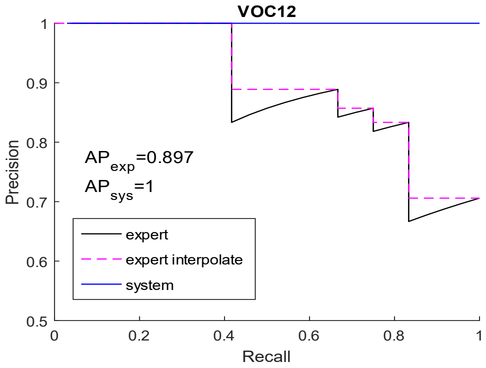

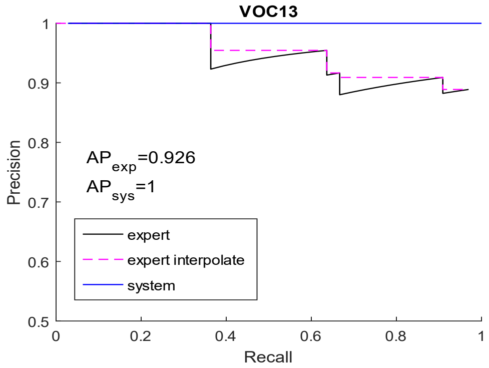

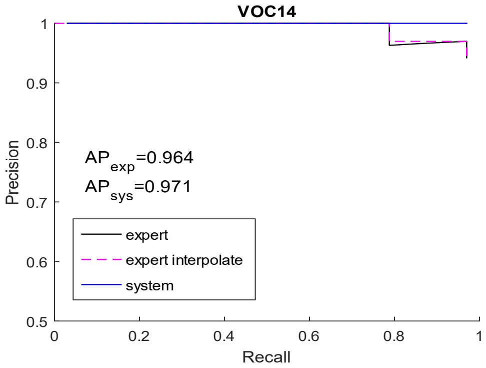

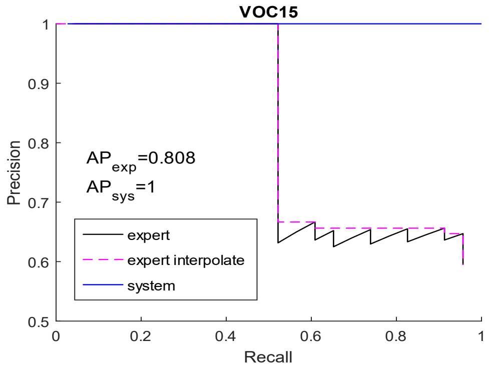

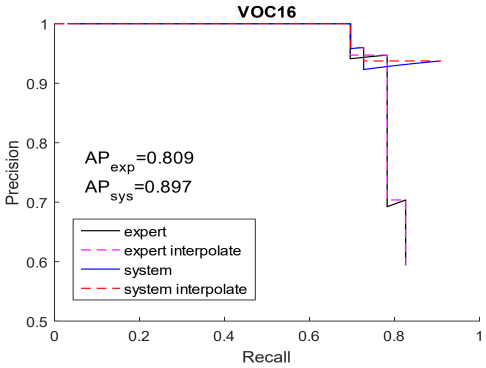

S4.17-S4.24 Figures: Precision against recall graphs for VOCs 17-24.

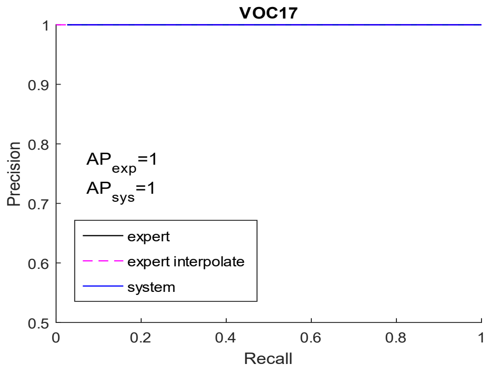

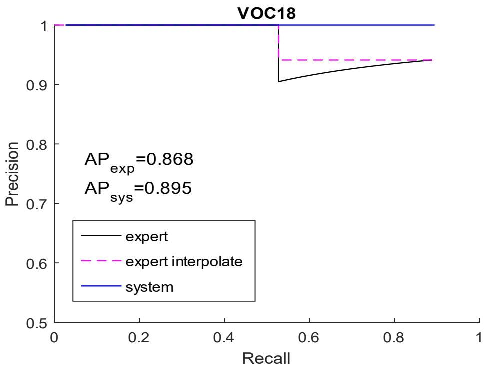

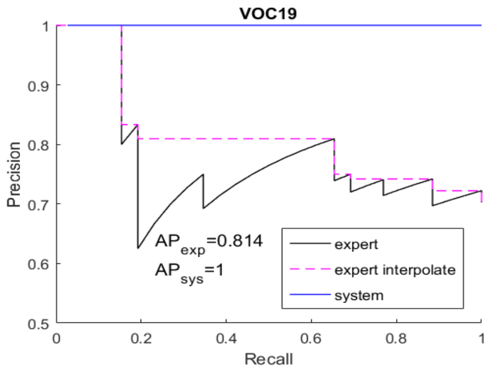

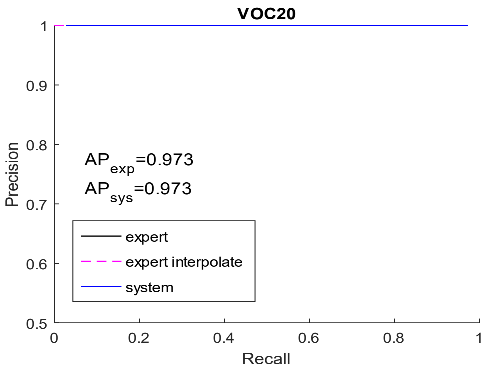

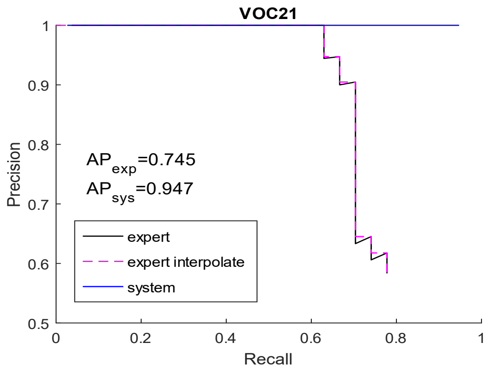

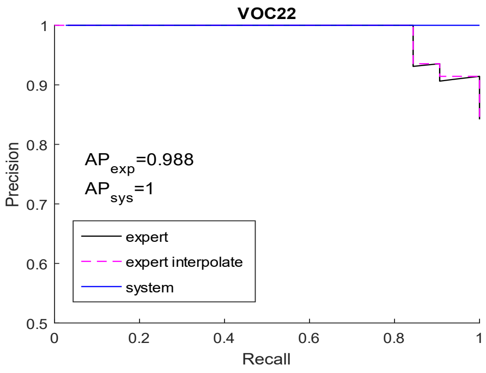

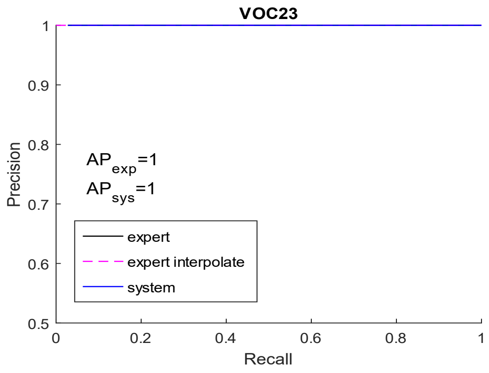

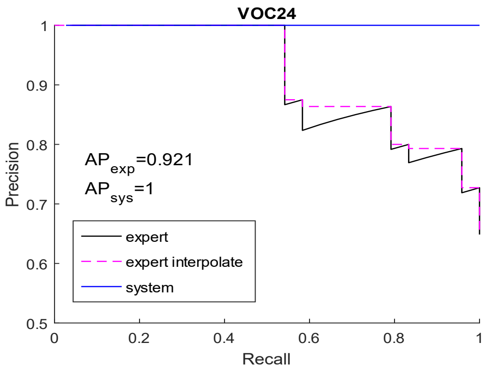

S4.25-S4.30 Figures: Precision against recall graphs for VOCs 25-30.

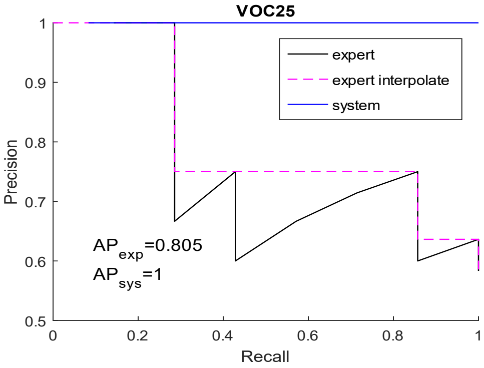

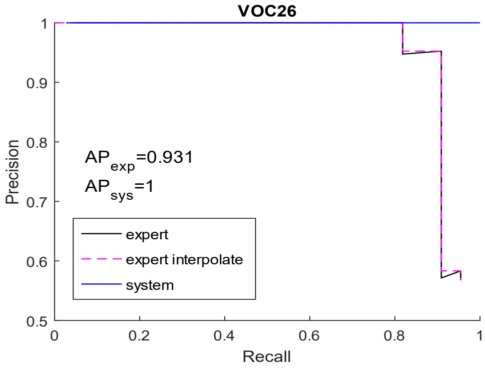

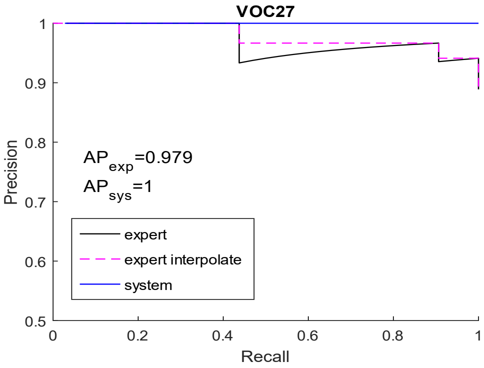

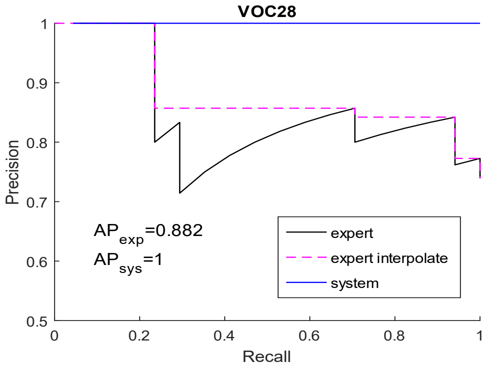

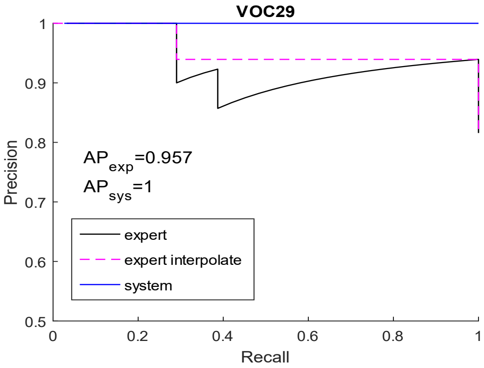

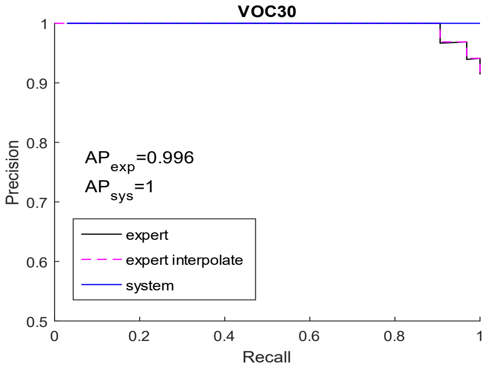

S4.2 Table: Results of the testing clinical sample analysis with the CNN-based system with VGG-8-1D model. Results evaluated per each testing sample.

Sample ID - number of a sample. *TP* - number of true positives detected per sample. *TTP* - number of tentative true positives detected per sample. *FP* - number of false positives detected per sample. *TTN* - number of tentative true negatives detected per sample. *FN* - number of false negatives detected per sample. *TN* - number of true negatives detected per sample (evaluation excluding RT position). *FP** - number of false positives detected per sample (evaluation excluding RT position). *TTP** - number of tentative true positives detected per sample (evaluation excluding RT position). *Sensitivity (expert)* - Sensitivity per sample in relation to expert-derived ground truth benchmark; tentative true positives (TTP) are considered FP, tentative true negatives (TTN) are considered FN; sensitivity = TP/(TP+FN+TTN). *Sensitivity (system)* - Sensitivity per sample in relation to system-derived correction benchmark; tentative true positives (TTP) are considered TP, tentative true negatives (TTN) are considered TN; sensitivity=(TP+TTP)/(TP+TTP+FN). *Specificity (expert)* - Specificity per sample in relation to expert-derived ground truth benchmark; tentative true positives (TTP*) are considered FP*; specificity=TN/TN+FP*+TTP*. *Specificity (system)* - Specificity per sample in relation to system-derived correction benchmark; tentative true positives (TTP*) are considered TP; specificity=TN/TN+FP*.

Sample ID	TP	TTP	FP	TTN	FN	TN	FP*	TTP*	Sensitivity (expert)	Sensitivity (system)	Specificity (expert)	Specificity (system)
1	26	2	0	0	1	1	0	2	0.963	0.9655	0.3333	1
2	24	4	0	0	1	1	0	4	0.96	0.9655	0.2	1
3	26	3	0	2	0	1	0	1	0.9286	1	0.5	1
4	27	2	0	0	0	1	0	2	1	1	0.3333	1
5	16	6	0	0	0	8	0	6	1	1	0.5714	1
6	24	4	0	1	0	2	0	3	0.96	1	0.4	1
7	26	2	0	0	1	1	0	2	0.963	0.9655	0.3333	1
8	24	3	0	0	1	2	0	3	0.96	0.9643	0.4	1
9	26	2	0	0	0	2	0	2	1	1	0.5	1
10	10	9	0	0	1	10	0	9	0.9091	0.95	0.5263	1
11	24	5	0	0	0	1	0	5	1	1	0.1667	1
12	24	5	0	1	0	1	0	4	0.96	1	0.2	1
13	22	6	1	0	0	1	1	6	1	1	0.125	0.5
14	25	4	0	0	0	1	0	4	1	1	0.2	1
15	24	3	1	0	1	2	0	3	0.96	0.9643	0.4	1
16	20	7	0	3	2	1	0	4	0.8	0.931	0.2	1
17	21	6	0	2	1	2	0	4	0.875	0.9643	0.3333	1
18	24	4	0	1	1	1	0	3	0.9231	0.9655	0.25	1
19	22	5	0	1	1	2	0	4	0.9167	0.9643	0.3333	1
20	24	3	0	0	0	3	0	3	1	1	0.5	1
21	18	9	0	0	0	3	0	9	1	1	0.25	1
22	25	5	0	0	0	0	0	5	1	1	0	-
23	21	7	0	1	0	2	0	6	0.9545	1	0.25	1
24	17	10	0	0	0	3	0	10	1	1	0.2308	1
25	22	5	0	0	0	3	0	5	1	1	0.375	1
26	16	10	0	0	0	4	0	10	1	1	0.2857	1
27	19	10	0	1	0	1	0	9	0.95	1	0.1	1
28	16	12	0	0	0	2	0	12	1	1	0.1429	1
29	16	11	0	1	0	3	0	10	0.9412	1	0.2308	1
30	11	15	0	0	0	4	0	15	1	1	0.2105	1
31	19	9	0	0	0	2	0	9	1	1	0.1818	1
32	21	8	0	0	0	1	0	8	1	1	0.1111	1
33	25	4	0	1	0	1	0	3	0.9615	1	0.25	1
34	24	6	0	1	0	0	0	5	0.96	1	0	-
35	24	3	0	0	0	3	0	3	1	1	0.5	1
36	20	6	0	1	0	4	0	5	0.9524	1	0.4444	1
37	22	6	0	1	0	2	0	5	0.9565	1	0.2857	1
38	21	5	0	0	0	4	0	5	1	1	0.4444	1
**TOTAL**	**816**	**226**	**2**	**18**	**11**	**86**	**1**	**208**	**0.9657**	**0.9896**	**0.2915**	**0.9885**

S4.2.1 Table: Detailed evaluation of the results of the CNN-based system with VGG-8-1D analysis (Stage 2) of the testing clinical sample Test-01-BS01.

Test_01_BS01 (Sample ID 1)

VOC Label	Start detection sRT	End detection eRT	Confidence $\mathcal{T}$
1	2.892	2.985	1.0000
2	3.033	3.134	1.0000
3	3.392	3.480	1.0000
4	3.482	3.589	1.0000
5	3.756	3.862	1.0000
6	3.891	4.027	0.9994
7	4.686	4.808	1.0000
8	4.935	5.025	1.0000
9	5.028	5.121	1.0000
10	5.216	5.341	1.0000
11	5.455	5.553	1.0000
12	5.556	5.670	0.9998
13	5.861	5.944	0.9998
14	6.275	6.371	1.0000
15	7.411	7.501	1.0000
16	7.838	7.914	0.9999
17	9.588	9.712	1.0000
18	10.243	10.359	1.0000
19	10.638	10.715	0.9974
20	11.364	11.447	1.0000
22	13.594	13.684	1.0000
23	16.248	16.325	1.0000
24	16.545	16.635	1.0000
26	19.712	19.837	1.0000
27	21.700	21.777	1.0000
28	22.808	22.896	1.0000
29	27.590	27.704	1.0000
30	41.972	42.084	1.0000

Colour	Class
(green)	**TP**
(yellow)	**TTP**
(red)	**FP**

	TN	TTN	FN
**VOC Label**	25	-	21

S4.2.2 Table: Detailed evaluation of the results of the CNN-based system with VGG-8-1D analysis (Stage 2) of the testing clinical sample Test-01-BS02.

Test_01_BS02 (Sample ID 2)

VOC Label	Start detection $s\mathcal{RT}$	End detection $e\mathcal{RT}$	Confidence $\mathcal{T}$
1	2.894	2.984	1.0000
2	3.040	3.130	1.0000
3	3.396	3.481	1.0000
4	3.483	3.587	1.0000
5	3.759	3.863	1.0000
6	3.895	4.025	0.9994
7	4.691	4.808	1.0000
8	4.935	5.026	1.0000
9	5.028	5.121	1.0000
10	5.219	5.341	1.0000
11	5.458	5.556	1.0000
12	5.559	5.670	0.9998
13	5.862	5.949	1.0000
14	6.278	6.371	1.0000
15	7.414	7.501	1.0000
16	7.841	7.918	1.0000
17	9.589	9.713	1.0000
18	10.246	10.358	1.0000
19	10.633	10.713	0.9995
20	11.368	11.450	1.0000
22	13.600	13.687	1.0000
23	16.251	16.328	1.0000
24	16.545	16.638	1.0000
26	19.716	19.838	1.0000
27	21.701	21.781	1.0000
28	22.815	22.899	1.0000
29	27.599	27.710	1.0000
30	41.970	42.082	1.0000

	**TP**
	**TTP**
	**FP**

	TN	TTN	FN
**VOC Label**	25	-	21

S4.2.3 Table: Detailed evaluation of the results of the CNN-based system with VGG-8-1D analysis (Stage 2) of the testing clinical sample Test-01-BS03.

Test_01_BS03 (Sample ID 3)

VOC Label	Start detection $s\mathcal{RT}$	End detection $e\mathcal{RT}$	Confidence $\mathcal{T}$
1	2.891	2.984	1.0000
2	3.034	3.130	1.0000
3	3.390	3.475	1.0000
4	3.478	3.584	1.0000
5	3.754	3.858	1.0000
6	3.884	4.038	0.9994
7	4.686	4.800	1.0000
8	4.930	5.021	1.0000
9	5.023	5.116	1.0000
10	5.217	5.336	1.0000
11	5.451	5.551	1.0000
12	5.554	5.665	0.9998
13	5.859	5.944	1.0000
14	6.273	6.366	1.0000
15	7.403	7.499	1.0000
16	7.830	7.917	1.0000
17	9.582	9.710	1.0000
18	10.237	10.354	1.0000
19	10.622	10.709	1.0000
20	11.359	11.444	1.0000
21	11.756	11.812	0.9544
22	13.588	13.681	1.0000
23	16.242	16.321	1.0000
24	16.536	16.629	1.0000
26	19.711	19.830	1.0000
27	21.690	21.773	1.0000
28	22.804	22.891	1.0000
29	27.590	27.704	1.0000
30	41.957	42.066	1.0000

Colour	Category
(green)	TP
(yellow)	TTP
(red)	FP

	TN	TTN	FN
**VOC Label**	25	6, 21	-

S4.2.4 Table: Detailed evaluation of the results of the CNN-based system with VGG-8-1D analysis (Stage 2) of the testing clinical sample Test-01-BS04.

Test_01_BS04 (Sample ID 4)

VOC Label	Start detection sRT	End detection eRT	Confidence $\mathcal{T}$
1	2.894	2.989	1.0000
2	3.035	3.138	1.0000
3	3.393	3.481	1.0000
4	3.484	3.590	1.0000
5	3.757	3.858	1.0000
6	3.882	4.023	0.9995
7	4.691	4.805	1.0000
8	4.933	5.026	1.0000
9	5.028	5.121	1.0000
10	5.219	5.341	1.0000
11	5.456	5.556	1.0000
12	5.559	5.670	0.9999
13	5.864	5.946	0.9994
14	6.281	6.365	1.0000
15	7.411	7.501	1.0000
16	7.835	7.923	1.0000
17	9.589	9.716	1.0000
18	10.246	10.358	1.0000
19	10.628	10.713	1.0000
20	11.365	11.450	1.0000
21	11.763	11.818	0.9576
22	13.595	13.688	1.0000
23	16.249	16.328	1.0000
24	16.543	16.636	1.0000
26	19.722	19.836	1.0000
27	21.700	21.779	1.0000
28	22.810	22.901	1.0000
29	27.592	27.711	1.0000
30	41.969	42.078	1.0000

Colour	Class
(green)	**TP**
(yellow)	**TTP**
(red)	**FP**

	TN	TTN	FN
**VOC Label**	25	-	-

S4.2.5 Table: Detailed evaluation of the results of the CNN-based system with VGG-8-1D analysis (Stage 2) of the testing clinical sample Test-01-ES.

Test_01_ES (Sample ID 5)

VOC Label	Start detection $s\mathcal{RT}$	End detection $e\mathcal{RT}$	Confidence $\mathcal{T}$
1	2.915	2.992	0.9973
2	3.056	3.109	0.9474
3	3.420	3.473	0.9933
4	3.475	3.597	1.0000
5	3.746	3.862	1.0000
6	3.865	3.966	0.9991
8	4.917	5.015	1.0000
9	5.018	5.103	1.0000
11	5.466	5.537	0.9980
12	5.553	5.657	1.0000
15	7.421	7.474	0.9243
16	7.840	7.928	1.0000
17	9.583	9.703	1.0000
18	10.230	10.341	1.0000
19	10.600	10.709	1.0000
21	11.331	11.419	0.9986
22	13.587	13.680	1.0000
24	16.537	16.627	1.0000
26	19.705	19.829	1.0000
28	22.803	22.885	1.0000
29	27.586	27.702	1.0000
30	41.957	42.048	1.0000

Colour	Class
green	TP
yellow	TTP
red	FP

	TN	TTN	FN
VOC Label	7, 10, 13, 14, 20, 23, 25, 27	-	-

S4.2.6 Table: Detailed evaluation of the results of the CNN-based system with VGG-8-1D analysis (Stage 2) of the testing clinical sample Test-02-BS01.

Test_02_BS01 (Sample ID 6)

VOC Label	Start detection $s\mathcal{RT}$	End detection $e\mathcal{RT}$	Confidence $\mathcal{T}$
1	2.881	2.982	1.0000
2	3.030	3.129	1.0000
3	3.370	3.477	1.0000
4	3.479	3.575	1.0000
5	3.745	3.856	1.0000
6	3.859	4.024	0.9995
7	4.693	4.781	1.0000
8	4.927	5.006	1.0000
9	5.009	5.110	1.0000
10	5.221	5.325	1.0000
11	5.447	5.532	0.9995
12	5.558	5.654	0.9996
13	5.853	5.927	1.0000
14	6.262	6.347	1.0000
15	7.389	7.464	0.9995
17	9.567	9.708	1.0000
18	10.226	10.321	1.0000
19	10.605	10.687	1.0000
20	11.347	11.432	1.0000
21	11.732	11.790	0.9656
22	13.563	13.659	1.0000
23	16.220	16.297	1.0000
24	16.516	16.601	1.0000
26	19.695	19.801	1.0000
27	21.669	21.746	1.0000
28	22.777	22.872	1.0000
29	27.565	27.684	1.0000
30	41.940	42.015	0.9917

	**TP**
	**TTP**
	**FP**

	TN	TTN	FN
**VOC Label**	16, 25	5	-

S4.2.7 Table: Detailed evaluation of the results of the CNN-based system with VGG-8-1D analysis (Stage 2) of the testing clinical sample Test-02-BS02.

Test_02_BS02 (Sample ID 7)

VOC Label	Start detection sRT	End detection eRT	Confidence T
1	2.881	2.982	1.0000
2	3.033	3.128	1.0000
3	3.367	3.476	1.0000
4	3.479	3.580	1.0000
5	3.747	3.850	1.0000
6	3.874	4.020	0.9997
7	4.691	4.784	1.0000
8	4.922	5.007	1.0000
9	5.009	5.110	1.0000
10	5.216	5.320	1.0000
11	5.453	5.527	0.9886
12	5.553	5.652	0.9996
13	5.856	5.928	1.0000
14	6.264	6.344	1.0000
15	7.394	7.463	0.9988
17	9.568	9.709	1.0000
18	10.224	10.324	1.0000
19	10.613	10.687	0.9998
20	11.342	11.432	1.0000
21	11.732	11.790	0.9567
22	13.568	13.659	1.0000
23	16.219	16.299	1.0000
24	16.521	16.598	1.0000
26	19.710	19.797	0.9999
27	21.671	21.747	1.0000
28	22.786	22.868	1.0000
29	27.564	27.676	1.0000
30	41.931	42.029	1.0000

(green)	**TP**
(yellow)	**TTP**
(red)	**FP**

	TN	TTN	FN
**VOC Label**	25	-	16

S4.2.8 Table: Detailed evaluation of the results of the CNN-based system with VGG-8-1D analysis (Stage 2) of the testing clinical sample Test-02-BS03.

Test_02_BS03 (Sample ID 8)

VOC Label	Start detection sRT	End detection eRT	Confidence $\mathcal{T}$
1	2.885	2.981	1.0000
2	3.034	3.132	1.0000
3	3.371	3.477	1.0000
4	3.480	3.581	1.0000
5	3.748	3.851	1.0000
6	3.872	4.037	1.0000
7	4.698	4.782	1.0000
8	4.923	5.008	1.0000
9	5.011	5.111	1.0000
10	5.220	5.321	1.0000
11	5.448	5.533	0.9993
12	5.555	5.653	0.9993
13	5.860	5.926	0.9999
14	6.271	6.343	1.0000
15	7.390	7.465	0.9994
17	9.570	9.707	1.0000
18	10.225	10.323	1.0000
19	10.617	10.691	1.0000
21	11.290	11.343	0.9795
20	11.346	11.433	1.0000
22	13.568	13.658	1.0000
23	16.222	16.299	1.0000
24	16.524	16.598	0.9999
26	19.702	19.800	1.0000
27	21.673	21.747	1.0000
28	22.783	22.868	1.0000
29	27.567	27.684	1.0000

Colour	
(green)	**TP**
(yellow)	**TTP**
(red)	**FP**

	TN	TTN	FN
**VOC Label**	25, 30	-	16

S4.2.9 Table: Detailed evaluation of the results of the CNN-based system with VGG-8-1D analysis (Stage 2) of the testing clinical sample Test-02-BS04.

Test_02_BS04 (Sample ID 9)

VOC Label	Start detection $s\mathcal{RT}$	End detection $e\mathcal{RT}$	Confidence $\mathcal{T}$
1	2.886	2.979	1.0000
2	3.035	3.133	1.0000
3	3.382	3.478	1.0000
4	3.481	3.584	1.0000
5	3.748	3.852	1.0000
6	3.873	4.075	1.0000
7	4.695	4.785	1.0000
8	4.923	5.008	1.0000
9	5.011	5.114	1.0000
10	5.218	5.324	1.0000
11	5.448	5.533	0.9995
12	5.555	5.655	0.9996
13	5.857	5.923	0.9985
14	6.273	6.342	1.0000
15	7.390	7.472	1.0000
17	9.569	9.707	1.0000
18	10.224	10.325	1.0000
19	10.611	10.688	1.0000
20	11.343	11.435	1.0000
21	11.732	11.790	0.9659
22	13.569	13.662	1.0000
23	16.220	16.299	1.0000
24	16.522	16.599	0.9999
26	19.711	19.798	1.0000
27	21.668	21.748	1.0000
28	22.784	22.869	1.0000
29	27.564	27.683	1.0000
30	41.926	42.029	1.0000

(green)	**TP**
(yellow)	**TTP**
(red)	**FP**

	TN	TTN	FN
**VOC Label**	16, 25	-	-

S4.2.10 Table: Detailed evaluation of the results of the CNN-based system with VGG-8-1D analysis (Stage 2) of the testing clinical sample Test-02-ES.

Test_02_ES (Sample ID 10)

VOC Label	Start detection $s\mathcal{RT}$	End detection $e\mathcal{RT}$	Confidence $\mathcal{T}$
1	2.897	2.996	1.0000
3	3.415	3.468	0.9900
4	3.471	3.590	1.0000
5	3.741	3.858	1.0000
6	3.861	3.927	0.9802
8	4.911	5.009	1.0000
9	5.012	5.094	1.0000
10	5.216	5.303	0.9872
12	5.569	5.648	0.9921
15	7.418	7.469	0.7724
16	7.840	7.890	0.8811
17	9.572	9.694	1.0000
18	10.223	10.329	1.0000
19	10.615	10.689	0.9947
21	11.333	11.402	0.9218
22	13.600	13.669	0.9975
26	19.720	19.802	0.9996
28	22.804	22.873	0.9980
29	27.570	27.676	0.9999

Colour	Legend
green	**TP**
yellow	**TTP**
red	**FP**

	TN	TTN	FN
**VOC Label**	2, 7, 11, 13, 14, 23, 24, 25, 27, 30	-	20

S4.2.11 Table: Detailed evaluation of the results of the CNN-based system with VGG-8-1D analysis (Stage 2) of the testing clinical sample Test-03-BS01.

Test_03_BS01 (Sample ID 11)

VOC Label	Start detection $\mathscr{sRT}$	End detection $\mathscr{eRT}$	Confidence $\mathscr{T}$
1	2.869	2.954	1.0000
2	3.005	3.087	1.0000
3	3.351	3.425	1.0000
4	3.428	3.539	1.0000
5	3.680	3.792	1.0000
6	3.805	3.959	0.9992
7	4.621	4.674	0.9886
8	4.829	4.906	1.0000
9	4.908	4.996	1.0000
10	5.126	5.230	1.0000
11	5.232	5.424	1.0000
12	5.426	5.530	0.9998
13	5.724	5.801	1.0000
14	6.122	6.210	1.0000
15	7.238	7.325	1.0000
16	7.662	7.745	1.0000
17	9.393	9.499	0.9999
18	10.035	10.150	1.0000
19	10.404	10.495	1.0000
20	11.129	11.222	1.0000
21	11.514	11.569	0.9034
22	13.355	13.445	1.0000
23	15.987	16.067	1.0000
24	16.295	16.383	1.0000
26	19.493	19.594	1.0000
27	21.435	21.509	1.0000
28	22.578	22.652	0.9973
29	27.334	27.448	1.0000
30	41.686	41.763	0.9923

	**TP**
	**TTP**
	**FP**

	TN	TTN	FN
**VOC Label**	25	-	-

S4.2.12 Table: Detailed evaluation of the results of the CNN-based system with VGG-8-1D analysis (Stage 2) of the testing clinical sample Test-03-BS02.

Test_03_BS02 (Sample ID 12)

VOC Label	Start detection sRT	End detection eRT	Confidence T
1	2.865	2.947	1.0000
2	3.003	3.082	1.0000
3	3.340	3.423	1.0000
4	3.425	3.539	1.0000
5	3.678	3.781	1.0000
6	3.813	3.949	0.9992
7	4.615	4.665	0.9573
8	4.820	4.905	1.0000
9	4.907	4.992	1.0000
10	5.112	5.226	1.0000
11	5.228	5.420	1.0000
12	5.422	5.523	0.9998
13	5.712	5.794	1.0000
14	6.110	6.205	1.0000
15	7.232	7.325	1.0000
16	7.662	7.739	1.0000
17	9.386	9.495	1.0000
18	10.031	10.143	1.0000
19	10.403	10.487	1.0000
20	11.121	11.217	1.0000
21	11.501	11.559	0.8859
22	13.349	13.440	1.0000
23	15.981	16.060	1.0000
24	16.294	16.371	1.0000
26	19.474	19.591	1.0000
27	21.421	21.503	1.0000
28	22.561	22.641	0.9997
29	27.328	27.432	1.0000
30	41.679	41.775	1.0000

Colour	Category
(green)	**TP**
(yellow)	**TTP**
(red)	**FP**

	TN	TTN	FN
**VOC Label**	25	15	-

S4.2.13 Table: Detailed evaluation of the results of the CNN-based system with VGG-8-1D analysis (Stage 2) of the testing clinical sample Test-03-BS03.

Test_03_BS03 (Sample ID 13)

VOC Label	Start detection sRT	End detection eRT	Confidence $\mathcal{T}$
1	2.865	2.952	1.0000
2	3.005	3.088	1.0000
3	3.343	3.425	1.0000
4	3.428	3.542	1.0000
5	3.683	3.784	1.0000
6	3.813	3.954	0.9993
7	4.623	4.673	0.9360
8	4.819	4.907	1.0000
9	4.910	4.997	1.0000
10	5.111	5.218	1.0000
11	5.321	5.422	1.0000
12	5.425	5.528	0.9999
13	5.717	5.799	1.0000
14	6.117	6.208	1.0000
15	7.237	7.312	0.9999
16	7.540	7.612	0.9951
17	9.384	9.498	1.0000
18	10.034	10.148	1.0000
19	10.402	10.490	1.0000
20	11.126	11.219	1.0000
21	11.511	11.567	0.9277
22	13.352	13.444	1.0000
23	15.985	16.062	1.0000
24	16.296	16.378	1.0000
26	19.465	19.590	1.0000
27	21.425	21.502	1.0000
28	22.573	22.642	0.9964
29	27.327	27.436	1.0000
30	41.672	41.781	1.0000

Colour	Class
(green)	TP
(yellow)	TTP
(red)	FP

	TN	TTN	FN
**VOC Label**	25	-	-

S4.2.14 Table: Detailed evaluation of the results of the CNN-based system with VGG-8-1D analysis (Stage 2) of the testing clinical sample Test-03-BS04.

Test_03_BS04 (Sample ID 14)

VOC Label	Start detection sRT	End detection eRT	Confidence $\mathcal{T}$
1	2.861	2.949	1.0000
2	3.010	3.093	1.0000
3	3.340	3.425	1.0000
4	3.427	3.542	1.0000
5	3.680	3.781	1.0000
6	3.810	3.953	0.9994
7	4.585	4.686	1.0000
8	4.816	4.901	1.0000
9	4.903	4.996	1.0000
10	5.094	5.214	1.0000
11	5.320	5.418	1.0000
12	5.421	5.522	0.9998
13	5.710	5.795	1.0000
14	6.113	6.204	1.0000
15	7.230	7.313	1.0000
16	7.657	7.716	0.9924
17	9.381	9.493	1.0000
18	10.026	10.148	1.0000
19	10.405	10.484	1.0000
20	11.121	11.216	1.0000
21	11.500	11.561	0.9249
22	13.356	13.435	1.0000
23	15.978	16.058	1.0000
24	16.299	16.368	0.9996
26	19.462	19.587	1.0000
27	21.421	21.504	1.0000
28	22.559	22.643	1.0000
29	27.322	27.439	1.0000
30	41.671	41.772	1.0000

Colour	Legend
(green)	TP
(yellow)	TTP
(red)	FP

	TN	TTN	FN
VOC Label	25	-	-

S4.2.15 Table: Detailed evaluation of the results of the CNN-based system with VGG-8-1D analysis (Stage 2) of the testing clinical sample Test-03-ES.

Test_03_ES (Sample ID 15)

VOC Label	Start detection sRT	End detection eRT	Confidence T
1	2.856	2.976	1.0000
2	3.034	3.090	0.9810
3	3.377	3.430	0.9750
4	3.432	3.539	1.0000
5	3.685	3.796	1.0000
6	3.799	3.953	1.0000
8	4.815	4.913	1.0000
9	4.916	5.003	1.0000
10	5.102	5.192	0.9998
11	5.340	5.420	0.9986
12	5.430	5.531	1.0000
13	5.730	5.804	1.0000
15	7.245	7.319	1.0000
16	7.552	7.616	0.9934
17	9.398	9.498	1.0000
18	10.053	10.132	1.0000
19	10.405	10.493	1.0000
21	11.081	11.163	0.9997
20	11.166	11.232	0.9673
22	13.356	13.451	1.0000
23	15.991	16.073	1.0000
24	16.296	16.389	1.0000
25	16.786	16.895	1.0000
26	19.483	19.598	1.0000
27	21.427	21.512	1.0000
28	22.563	22.655	1.0000
29	27.338	27.447	1.0000
30	41.681	41.788	1.0000

Colour	Class
(green)	**TP**
(yellow)	**TTP**
(red)	**FP**

	**TN**	**TTN**	**FN**
**VOC Label**	7, 14	-	16

S4.2.16 Table: Detailed evaluation of the results of the CNN-based system with VGG-8-1D analysis (Stage 2) of the testing clinical sample Test-04-BS01.

Test_04_BS01 (Sample ID 16)

VOC Label	Start detection sRT	End detection eRT	Confidence T
1	3.146	3.306	1.0000
2	3.373	3.509	1.0000
3	4.005	4.088	1.0000
4	4.090	4.176	1.0000
5	4.576	4.683	1.0000
6	4.827	4.920	0.9980
7	5.914	6.023	1.0000
8	6.258	6.351	1.0000
9	6.354	6.453	1.0000
10	6.621	6.728	1.0000
11	6.968	7.048	0.9989
12	7.051	7.123	1.0000
13	7.521	7.601	1.0000
15	9.341	9.419	0.9990
16	9.743	9.844	1.0000
17	11.633	11.734	1.0000
19	12.815	12.900	1.0000
21	13.609	13.691	0.9686
20	13.694	13.776	1.0000
22	15.903	15.985	1.0000
23	18.679	18.753	1.0000
24	18.918	18.995	1.0000
26	22.177	22.241	0.9718
27	24.161	24.243	1.0000
28	25.393	25.456	0.9490
29	30.186	30.303	1.0000
30	44.662	44.793	1.0000

Colour	
(green)	**TP**
(yellow)	**TTP**
(red)	**FP**

	TN	TTN	FN
**VOC Label**	25	10, 16, 21	14, 18

S4.2.17 Table: Detailed evaluation of the results of the CNN-based system with VGG-8-1D analysis (Stage 2) of the testing clinical sample Test-04-BS02.

Test_04_BS02 (Sample ID 17)

VOC Label	Start detection sRT	End detection eRT	Confidence T
1	3.138	3.303	1.0000
2	3.370	3.519	1.0000
3	3.994	4.085	1.0000
4	4.087	4.181	1.0000
5	4.573	4.792	1.0000
6	4.824	4.888	0.9978
7	5.915	6.030	1.0000
8	6.259	6.357	1.0000
9	6.360	6.461	1.0000
10	6.629	6.738	1.0000
11	6.974	7.059	0.9980
12	7.062	7.128	1.0000
13	7.535	7.612	1.0000
14	8.053	8.146	1.0000
15	9.323	9.427	1.0000
16	9.748	9.846	1.0000
17	11.635	11.730	1.0000
19	12.819	12.901	1.0000
21	13.634	13.700	0.9703
20	13.703	13.785	1.0000
22	15.903	15.988	1.0000
23	18.682	18.759	1.0000
24	18.921	18.998	1.0000
26	22.177	22.244	0.9728
27	24.164	24.247	1.0000
29	30.184	30.311	1.0000
30	44.669	44.790	1.0000

Colour	Class
(green)	TP
(yellow)	TTP
(red)	FP

	TN	TTN	FN
VOC Label	25, 28	13, 21	18

S4.2.18 Table: Detailed evaluation of the results of the CNN-based system with VGG-8-1D analysis (Stage 2) of the testing clinical sample Test-04-BS03.

Test_04_BS03 (Sample ID 18)

VOC Label	Start detection sRT	End detection eRT	Confidence T
1	3.153	3.300	1.0000
2	3.372	3.521	1.0000
3	3.993	4.087	1.0000
4	4.089	4.183	1.0000
5	4.575	4.794	1.0000
6	4.826	4.893	0.9984
7	5.914	6.015	0.9996
8	6.273	6.364	1.0000
9	6.366	6.467	1.0000
10	6.632	6.720	1.0000
11	6.980	7.065	0.9950
12	7.068	7.142	1.0000
13	7.541	7.615	1.0000
14	8.058	8.151	1.0000
15	9.326	9.432	1.0000
16	9.753	9.849	1.0000
17	11.638	11.736	1.0000
19	12.822	12.906	1.0000
21	13.618	13.705	0.9994
20	13.708	13.798	1.0000
22	15.906	15.991	1.0000
23	18.685	18.762	1.0000
24	18.930	19.001	0.9994
26	22.184	22.248	0.9743
27	24.172	24.251	1.0000
28	25.356	25.441	0.9996
29	30.190	30.320	1.0000
30	44.679	44.789	1.0000

Colour	Legend
(green)	**TP**
(yellow)	**TTP**
(red)	**FP**

	TN	TTN	FN
**VOC Label**	25	21	18

S4.2.19 Table: Detailed evaluation of the results of the CNN-based system with VGG-8-1D analysis (Stage 2) of the testing clinical sample Test-04-BS04.

Test_04_BS04 (Sample ID 19)

VOC Label	Start detection sRT	End detection eRT	Confidence T
1	3.139	3.301	1.0000
2	3.368	3.517	1.0000
3	3.995	4.085	1.0000
4	4.088	4.179	1.0000
5	4.571	4.784	1.0000
6	4.825	4.881	0.9816
7	5.927	6.002	0.9567
8	6.258	6.357	1.0000
9	6.359	6.458	1.0000
10	6.626	6.733	1.0000
11	6.773	6.837	0.9674
12	7.056	7.130	1.0000
13	7.532	7.609	1.0000
14	8.053	8.143	1.0000
15	9.332	9.428	1.0000
16	9.755	9.848	1.0000
17	11.640	11.740	1.0000
19	12.821	12.909	1.0000
21	13.602	13.702	0.9953
20	13.705	13.790	1.0000
22	15.908	15.993	1.0000
23	18.687	18.764	1.0000
24	18.924	19.003	1.0000
26	22.183	22.247	0.9715
27	24.171	24.253	1.0000
29	30.191	30.321	1.0000
30	44.678	44.795	1.0000

Colour	Class
(green)	TP
(yellow)	TTP
(red)	FP

	TN	TTN	FN
VOC Label	25, 28	11	18

S4.2.20 Table: Detailed evaluation of the results of the CNN-based system with VGG-8-1D analysis (Stage 2) of the testing clinical sample Test-04-ES.

Test_04_ES (Sample ID 20)

VOC Label	Start detection $s\mathcal{RT}$	End detection $e\mathcal{RT}$	Confidence $\mathcal{T}$
1	3.129	3.336	1.0000
2	3.416	3.506	1.0000
3	4.037	4.088	0.9765
4	4.090	4.196	1.0000
5	4.592	4.709	1.0000
6	4.786	4.910	1.0000
8	6.277	6.378	1.0000
9	6.381	6.476	1.0000
10	6.686	6.760	0.9926
12	7.039	7.140	1.0000
13	7.567	7.628	0.9364
14	8.079	8.156	0.9999
15	9.323	9.429	1.0000
16	9.758	9.862	1.0000
17	11.636	11.748	1.0000
18	12.369	12.464	1.0000
19	12.814	12.918	1.0000
21	13.581	13.682	1.0000
20	13.706	13.799	1.0000
22	15.906	16.005	1.0000
23	18.692	18.772	1.0000
24	18.923	19.016	1.0000
25	19.447	19.561	1.0000
26	22.271	22.332	0.9467
27	24.179	24.261	1.0000
28	25.348	25.446	1.0000
29	30.200	30.325	1.0000

Legend	
(green)	**TP**
(yellow)	**TTP**
(red)	**FP**

	TN	TTN	FN
**VOC Label**	7, 11, 30	-	-

S4.2.21 Table: Detailed evaluation of the results of the CNN-based system with VGG-8-1D analysis (Stage 2) of the testing clinical sample Test-05-BS01.

Test_05_BS01 (Sample ID 21)

VOC Label	Start detection sRT	End detection eRT	Confidence T
1	3.114	3.256	0.9961
2	3.382	3.513	1.0000
3	3.934	4.024	1.0000
4	4.027	4.107	1.0000
5	4.480	4.589	1.0000
6	4.731	4.811	0.9867
7	5.757	5.848	0.9994
8	6.093	6.181	1.0000
9	6.184	6.280	1.0000
10	6.437	6.557	1.0000
11	6.589	6.744	0.9999
13	7.329	7.406	1.0000
14	7.828	7.924	1.0000
15	9.071	9.162	1.0000
16	9.468	9.572	0.9982
17	11.300	11.433	1.0000
18	12.033	12.129	1.0000
19	12.509	12.593	1.0000
21	13.299	13.376	0.9279
20	13.389	13.487	1.0000
22	15.590	15.664	0.9999
23	18.360	18.440	1.0000
24	18.588	18.663	0.9999
26	21.881	21.945	0.9610
27	23.838	23.918	1.0000
29	29.791	29.903	1.0000
30	44.251	44.347	1.0000

(green)	**TP**
(yellow)	**TTP**
(red)	**FP**

	TN	TTN	FN
**VOC Label**	12, 25, 28	-	-

S4.2.22 Table: Detailed evaluation of the results of the CNN-based system with VGG-8-1D analysis (Stage 2) of the testing clinical sample Test-05-BS02.

Test_05_BS02 (Sample ID 22)

VOC Label	Start detection $s\mathcal{RT}$	End detection $e\mathcal{RT}$	Confidence $\mathcal{T}$
1	3.117	3.271	1.0000
2	3.338	3.492	1.0000
3	3.930	4.025	1.0000
4	4.028	4.110	1.0000
5	4.473	4.699	1.0000
6	4.723	4.784	0.9861
7	5.749	5.866	1.0000
8	6.091	6.184	1.0000
9	6.187	6.280	1.0000
10	6.428	6.558	1.0000
11	6.561	6.688	0.9988
12	6.855	6.924	0.9733
13	7.340	7.409	1.0000
14	7.839	7.929	1.0000
15	9.056	9.154	1.0000
16	9.475	9.555	0.9992
17	11.313	11.422	1.0000
18	12.037	12.146	1.0000
19	12.504	12.594	1.0000
21	13.275	13.382	1.0000
20	13.384	13.485	1.0000
22	15.578	15.668	1.0000
23	18.362	18.444	1.0000
24	18.582	18.667	1.0000
25	19.095	19.180	1.0000
26	21.885	21.951	0.9832
27	23.839	23.921	1.0000
28	24.949	25.050	1.0000
29	29.787	29.920	1.0000
30	44.246	44.371	1.0000

Legend	
(green)	TP
(yellow)	TTP
(red)	FP

	TN	TTN	FN
VOC Label	-	-	-

S4.2.23 Table: Detailed evaluation of the results of the CNN-based system with VGG-8-1D analysis (Stage 2) of the testing clinical sample Test-05-BS03.

Test_05_BS03 (Sample ID 23)

VOC Label	Start detection sRT	End detection eRT	Confidence T
1	3.072	3.267	1.0000
2	3.331	3.485	1.0000
3	3.912	4.011	1.0000
4	4.013	4.096	1.0000
5	4.461	4.680	1.0000
6	4.712	4.773	0.9886
7	5.750	5.843	0.9889
8	6.080	6.174	1.0000
9	6.176	6.273	1.0000
10	6.425	6.545	1.0000
11	6.587	6.750	0.9999
12	6.854	6.929	1.0000
13	7.319	7.396	1.0000
14	7.824	7.920	1.0000
15	9.065	9.161	1.0000
16	9.464	9.562	0.9989
17	11.319	11.428	1.0000
18	12.038	12.147	1.0000
19	12.502	12.598	1.0000
21	13.282	13.383	1.0000
20	13.385	13.481	1.0000
22	15.581	15.669	1.0000
23	18.362	18.441	1.0000
24	18.579	18.669	1.0000
26	21.884	21.948	0.9723
27	23.837	23.920	1.0000
29	29.787	29.917	1.0000
30	44.244	44.364	1.0000

(green)	TP
(yellow)	TTP
(red)	FP

	TN	TTN	FN
VOC Label	25, 28	11	-

S4.2.24 Table: Detailed evaluation of the results of the CNN-based system with VGG-8-1D analysis (Stage 2) of the testing clinical sample Test-05-BS04.

Test_05_BS04 (Sample ID 24)

VOC Label	Start detection $s\mathcal{RT}$	End detection $e\mathcal{RT}$	Confidence $\mathcal{T}$
1	3.153	3.269	0.9892
2	3.416	3.539	1.0000
3	3.984	4.069	1.0000
4	4.072	4.157	1.0000
5	4.533	4.637	1.0000
6	4.778	4.842	0.8617
7	5.810	5.921	1.0000
8	6.151	6.236	1.0000
9	6.239	6.329	1.0000
10	6.495	6.612	1.0000
11	6.660	6.809	0.9987
13	7.379	7.464	0.9995
14	7.892	7.977	1.0000
15	9.144	9.211	0.9907
16	9.522	9.621	0.9548
17	11.353	11.491	1.0000
18	12.105	12.203	0.9999
19	12.578	12.658	0.9999
21	13.351	13.449	1.0000
20	13.452	13.547	1.0000
22	15.648	15.736	1.0000
23	18.425	18.502	1.0000
24	18.651	18.736	1.0000
26	21.942	22.003	0.9537
27	23.902	23.984	1.0000
29	29.880	29.981	0.9998
30	44.327	44.458	1.0000

Colour	Meaning
(green)	TP
(yellow)	TTP
(red)	FP

	TN	TTN	FN
VOC Label	12, 25, 28	-	-

S4.2.25 Table: Detailed evaluation of the results of the CNN-based system with VGG-8-1D analysis (Stage 2) of the testing clinical sample Test-05-ES.

Test_05_ES (Sample ID 25)

VOC Label	Start detection sRT	End detection eRT	Confidence T
1	3.121	3.283	1.0000
2	3.357	3.474	1.0000
3	3.941	4.005	0.9999
4	4.007	4.119	1.0000
5	4.480	4.596	1.0000
6	4.599	4.779	1.0000
8	6.087	6.187	1.0000
9	6.190	6.285	1.0000
12	6.816	6.925	1.0000
13	7.341	7.410	0.9197
14	7.845	7.919	0.9998
15	9.046	9.155	1.0000
16	9.473	9.576	1.0000
17	11.313	11.421	1.0000
18	12.039	12.143	1.0000
19	12.493	12.593	1.0000
21	13.264	13.365	1.0000
20	13.381	13.468	1.0000
22	15.571	15.667	1.0000
23	18.362	18.445	1.0000
24	18.575	18.668	1.0000
25	19.076	19.190	1.0000
26	21.858	21.966	1.0000
27	23.835	23.920	1.0000
28	24.947	25.048	1.0000
29	29.788	29.910	1.0000
30	44.239	44.364	1.0000

Colour	Class
(green)	**TP**
(yellow)	**TTP**
(red)	**FP**

	**TN**	**TTN**	**FN**
**VOC Label**	7, 10, 11	-	-

S4.2.26 Table: Detailed evaluation of the results of the CNN-based system with VGG-8-1D analysis (Stage 2) of the testing clinical sample Test-06-BS01.

Test_06_BS01 (Sample ID 26)

VOC Label	Start detection sRT	End detection eRT	Confidence T
1	3.140	3.242	0.9999
2	3.386	3.468	0.9995
3	3.925	3.991	0.9999
4	3.994	4.071	0.9999
5	4.437	4.530	1.0000
6	4.679	4.791	0.9949
7	5.656	5.778	1.0000
8	5.997	6.072	1.0000
9	6.074	6.154	0.9999
10	6.328	6.424	1.0000
11	6.496	6.741	0.9988
12	7.062	7.120	0.9814
13	7.190	7.275	1.0000
14	7.679	7.775	1.0000
17	11.097	11.212	0.9996
18	11.837	11.904	0.9946
21	13.089	13.171	0.9997
20	13.174	13.249	1.0000
22	15.364	15.443	1.0000
23	18.133	18.210	1.0000
24	18.363	18.425	0.9946
25	19.030	19.104	0.9975
26	21.665	21.729	0.9595
27	23.600	23.677	1.0000
29	29.546	29.618	0.9659
30	43.939	44.056	1.0000

Colour	Category
green	TP
yellow	TTP
red	FP

	TN	TTN	FN
VOC Label	15, 16, 19, 28	-	-

S4.2.27 Table: Detailed evaluation of the results of the CNN-based system with VGG-8-1D analysis (Stage 2) of the testing clinical sample Test-06-BS02.

Test_06_BS02 (Sample ID 27)

VOC Label	Start detection sRT	End detection eRT	Confidence T
1	3.084	3.238	1.0000
2	3.305	3.432	1.0000
3	3.867	3.944	1.0000
4	3.947	4.037	1.0000
5	4.386	4.602	1.0000
6	4.634	4.704	0.9990
7	5.624	5.741	1.0000
8	5.962	6.047	1.0000
9	6.050	6.140	1.0000
10	6.298	6.407	1.0000
11	6.648	6.719	0.9998
12	7.034	7.108	0.9991
13	7.180	7.257	1.0000
14	7.669	7.759	1.0000
15	8.887	8.977	1.0000
16	9.306	9.372	0.9960
17	11.109	11.218	1.0000
18	11.831	11.923	1.0000
19	12.299	12.368	1.0000
21	13.082	13.174	1.0000
20	13.177	13.254	1.0000
22	15.366	15.443	1.0000
23	18.142	18.222	1.0000
24	18.367	18.431	0.9993
25	18.930	19.026	0.9999
26	21.675	21.741	0.9767
27	23.611	23.687	1.0000
29	29.551	29.625	0.9970
30	43.945	44.075	1.0000

(green)	TP
(yellow)	TTP
(red)	FP

	TN	TTN	FN
VOC Label	28	12	-

S4.2.28 Table: Detailed evaluation of the results of the CNN-based system with VGG-8-1D analysis (Stage 2) of the testing clinical sample Test-06-BS03.

Test_06_BS03 (Sample ID 28)

VOC Label	Start detection $s\mathcal{RT}$	End detection $e\mathcal{RT}$	Confidence $\mathcal{T}$
1	3.085	3.237	1.0000
2	3.298	3.424	1.0000
3	3.866	3.941	1.0000
4	3.943	4.028	1.0000
5	4.383	4.599	1.0000
6	4.631	4.703	0.9978
7	5.621	5.733	1.0000
8	5.952	6.040	1.0000
9	6.043	6.136	1.0000
10	6.285	6.400	1.0000
11	6.627	6.702	0.9997
12	6.705	6.782	1.0000
13	7.164	7.244	1.0000
14	7.655	7.743	1.0000
15	8.882	8.978	1.0000
16	9.305	9.371	0.9859
17	11.112	11.221	1.0000
18	11.834	11.919	1.0000
19	12.292	12.372	1.0000
21	13.080	13.165	1.0000
20	13.167	13.252	1.0000
22	15.364	15.441	1.0000
23	18.140	18.214	1.0000
24	18.357	18.423	1.0000
26	21.672	21.736	0.9743
27	23.615	23.676	0.9971
29	29.550	29.624	0.9974
30	43.944	44.069	1.0000

Colour	Label
(green)	**TP**
(yellow)	**TTP**
(red)	**FP**

	TN	TTN	FN
**VOC Label**	25, 28	-	-

S4.2.29 Table: Detailed evaluation of the results of the CNN-based system with VGG-8-1D analysis (Stage 2) of the testing clinical sample Test-06-BS04.

Test_06_BS04 (Sample ID 29)

VOC Label	Start detection sRT	End detection eRT	Confidence T
1	3.084	3.236	1.0000
2	3.300	3.433	1.0000
3	3.865	3.945	1.0000
4	3.948	4.030	1.0000
5	4.382	4.601	1.0000
6	4.633	4.697	0.9976
7	5.625	5.743	1.0000
8	5.953	6.047	1.0000
9	6.049	6.143	1.0000
10	6.292	6.402	1.0000
11	6.479	6.534	0.9930
12	7.035	7.109	0.9991
13	7.178	7.255	1.0000
14	7.670	7.758	1.0000
15	8.886	8.976	1.0000
17	11.117	11.220	1.0000
18	11.833	11.931	1.0000
19	12.302	12.373	0.9999
21	13.082	13.172	1.0000
20	13.174	13.259	1.0000
22	15.366	15.448	1.0000
23	18.142	18.222	1.0000
24	18.370	18.423	0.9662
26	21.678	21.741	0.9768
27	23.616	23.688	1.0000
29	29.546	29.625	0.9747
30	43.952	44.076	1.0000

Colour	Class
(green)	**TP**
(yellow)	**TTP**
(red)	**FP**

	TN	TTN	FN
**VOC Label**	16, 25, 28	11	-

S4.2.30 Table: Detailed evaluation of the results of the CNN-based system with VGG-8-1D analysis (Stage 2) of the testing clinical sample Test-06-ES.

Test_06_ES (Sample ID 30)

VOC Label	Start detection $s\mathcal{RT}$	End detection $e\mathcal{RT}$	Confidence $\mathcal{T}$
1	3.163	3.242	0.9999
3	3.882	3.938	0.9968
4	3.940	4.041	1.0000
5	4.398	4.509	1.0000
8	5.971	6.055	1.0000
9	6.058	6.140	1.0000
11	6.486	6.545	0.9931
12	7.044	7.110	0.9981
13	7.187	7.256	0.9894
14	7.689	7.758	1.0000
15	8.879	8.963	1.0000
16	9.304	9.365	0.9837
17	11.109	11.209	1.0000
18	11.828	11.923	1.0000
19	12.291	12.376	1.0000
21	13.068	13.174	1.0000
20	13.179	13.240	0.9968
22	15.350	15.446	1.0000
23	18.142	18.219	1.0000
24	18.351	18.433	1.0000
25	18.862	18.955	1.0000
26	21.680	21.747	0.9903
27	23.613	23.682	0.9999
28	24.704	24.778	0.9999
29	29.529	29.630	1.0000
30	43.953	44.065	1.0000

Legend	
(green)	**TP**
(yellow)	**TTP**
(red)	**FP**

	TN	TTN	FN
**VOC Label**	2, 6, 7, 10	-	-

S4.2.31 Table: Detailed evaluation of the results of the CNN-based system with VGG-8-1D analysis (Stage 2) of the testing clinical sample Test-07-BS01.

Test_07_BS01 (Sample ID 31)

VOC Label	Start detection $s\mathcal{RT}$	End detection $e\mathcal{RT}$	Confidence $\mathcal{T}$
1	3.075	3.211	1.0000
2	3.283	3.445	1.0000
3	3.803	3.901	1.0000
4	3.904	3.989	1.0000
5	4.312	4.416	1.0000
6	4.558	4.624	0.9989
7	5.513	5.609	0.9943
8	5.825	5.912	1.0000
9	5.915	6.005	1.0000
10	6.173	6.258	1.0000
11	6.260	6.430	1.0000
12	6.571	6.627	0.9778
13	7.012	7.091	1.0000
14	7.489	7.585	1.0000
15	8.675	8.769	1.0000
16	9.094	9.158	0.9159
17	10.861	10.970	1.0000
18	11.574	11.683	1.0000
19	12.041	12.131	1.0000
21	12.821	12.919	1.0000
20	12.921	12.990	1.0000
22	15.087	15.178	1.0000
23	17.875	17.949	1.0000
24	18.074	18.159	1.0000
26	21.421	21.485	0.9711
27	23.328	23.408	1.0000
29	29.190	29.312	1.0000
30	43.589	43.709	1.0000

	**TP**
	**TTP**
	**FP**

	TN	TTN	FN
**VOC Label**	25, 28	-	-

S4.2.32 Table: Detailed evaluation of the results of the CNN-based system with VGG-8-1D analysis (Stage 2) of the testing clinical sample Test-07-BS02.

Test_07_BS02 (Sample ID 32)

VOC Label	Start detection sRT	End detection eRT	Confidence T
1	3.083	3.227	1.0000
2	3.291	3.448	1.0000
3	3.811	3.907	1.0000
4	3.909	3.997	1.0000
5	4.320	4.534	1.0000
6	4.566	4.622	0.9804
7	5.535	5.623	0.7864
8	5.834	5.922	1.0000
9	5.924	6.013	1.0000
10	6.173	6.282	1.0000
11	6.340	6.401	0.9814
12	6.590	6.646	0.9430
13	7.023	7.105	1.0000
14	7.504	7.597	1.0000
15	8.690	8.789	1.0000
16	9.103	9.191	0.9943
17	10.882	10.991	1.0000
18	11.595	11.707	1.0000
19	12.057	12.150	1.0000
21	12.837	12.938	1.0000
20	12.940	13.012	1.0000
22	15.107	15.200	1.0000
23	17.893	17.972	1.0000
24	18.089	18.179	1.0000
25	18.697	18.747	0.8669
26	21.440	21.504	0.9678
27	23.345	23.428	1.0000
29	29.215	29.330	1.0000
30	43.615	43.724	1.0000

TP
TTP
FP

	TN	TTN	FN
**VOC Label**	28	-	-

S4.2.33 Table: Detailed evaluation of the results of the CNN-based system with VGG-8-1D analysis (Stage 2) of the testing clinical sample Test-07-BS03.

Test_07_BS03 (Sample ID 33)

VOC Label	Start detection sRT	End detection eRT	Confidence T
1	3.096	3.234	1.0000
2	3.301	3.441	1.0000
3	3.834	3.927	1.0000
4	3.930	4.015	1.0000
5	4.333	4.550	1.0000
6	4.553	4.633	0.9933
7	5.521	5.635	1.0000
8	5.845	5.935	1.0000
9	5.938	6.028	1.0000
10	6.171	6.283	1.0000
11	6.330	6.423	0.9995
12	6.540	6.635	0.9233
13	7.041	7.116	1.0000
14	7.516	7.606	1.0000
15	8.670	8.779	1.0000
16	9.091	9.187	1.0000
17	10.873	10.982	1.0000
18	11.581	11.706	1.0000
19	12.061	12.141	1.0000
21	12.830	12.931	1.0000
20	12.934	13.019	1.0000
22	15.111	15.188	1.0000
23	17.890	17.969	1.0000
24	18.108	18.169	0.9987
25	18.588	18.670	0.9997
26	21.276	21.345	0.9883
27	23.345	23.424	1.0000
29	29.214	29.322	1.0000
30	43.599	43.732	1.0000

(green)	TP
(yellow)	TTP
(red)	FP

	TN	TTN	FN
VOC Label	28	11	-

S4.2.34 Table: Detailed evaluation of the results of the CNN-based system with VGG-8-1D analysis (Stage 2) of the testing clinical sample Test-07-BS04.

Test_07_BS04 (Sample ID 34)

VOC Label	Start detection sRT	End detection eRT	Confidence T
1	3.111	3.233	1.0000
2	3.300	3.443	1.0000
3	3.833	3.921	1.0000
4	3.924	4.003	1.0000
5	4.332	4.550	1.0000
6	4.571	4.632	0.9834
7	5.521	5.632	1.0000
8	5.844	5.935	1.0000
9	5.937	6.025	1.0000
10	6.171	6.282	1.0000
11	6.325	6.417	0.9962
12	6.550	6.635	0.9711
13	7.041	7.115	1.0000
14	7.516	7.603	1.0000
15	8.675	8.781	1.0000
16	9.091	9.192	1.0000
17	10.876	10.984	1.0000
18	11.583	11.708	1.0000
19	12.061	12.143	1.0000
21	12.833	12.933	1.0000
20	12.936	13.018	1.0000
22	15.111	15.190	1.0000
23	17.892	17.974	1.0000
24	18.096	18.176	1.0000
25	18.584	18.675	1.0000
26	21.438	21.504	0.9657
27	23.347	23.432	1.0000
28	24.411	24.486	0.9964
29	29.211	29.336	1.0000
30	43.634	43.744	1.0000

Colour	Class
(green)	TP
(yellow)	TTP
(red)	FP

	TN	TTN	FN
VOC Label	-	26	-

S4.2.35 Table: Detailed evaluation of the results of the CNN-based system with VGG-8-1D analysis (Stage 2) of the testing clinical sample Test-07-ES.

Test_07_ES (Sample ID 35)

VOC Label	Start detection sRT	End detection eRT	Confidence T
1	3.095	3.241	1.0000
2	3.315	3.422	1.0000
3	3.838	3.899	1.0000
4	3.902	4.005	1.0000
5	4.334	4.451	1.0000
6	4.517	4.639	1.0000
8	5.841	5.934	1.0000
9	5.936	6.026	1.0000
10	6.201	6.265	0.9956
12	6.528	6.636	1.0000
13	7.037	7.111	0.9996
14	7.517	7.604	1.0000
15	8.675	8.779	1.0000
16	9.089	9.190	1.0000
17	10.870	10.979	1.0000
18	11.586	11.692	1.0000
19	12.053	12.143	1.0000
21	12.827	12.925	1.0000
20	12.928	13.018	1.0000
22	15.099	15.192	1.0000
23	17.890	17.970	1.0000
24	18.084	18.177	1.0000
25	18.567	18.679	1.0000
26	21.282	21.388	1.0000
27	23.345	23.427	1.0000
29	29.218	29.332	1.0000
30	43.613	43.722	1.0000

(green)	TP
(yellow)	TTP
(red)	FP

	TN	TTN	FN
VOC Label	7, 11, 28	-	-

S4.2.36 Table: Detailed evaluation of the results of the CNN-based system with VGG-8-1D analysis (Stage 2) of the testing clinical sample Test-08-BS01.

Test_08_BS01 (Sample ID 36)

VOC Label	Start detection sRT	End detection eRT	Confidence T
1	3.071	3.202	1.0000
2	3.266	3.399	1.0000
3	3.754	3.839	1.0000
4	3.842	3.927	1.0000
5	4.226	4.439	1.0000
6	4.471	4.543	0.9946
7	5.356	5.478	1.0000
8	5.677	5.760	1.0000
9	5.762	5.858	1.0000
10	5.983	6.100	1.0000
11	6.158	6.291	1.0000
13	6.828	6.910	1.0000
14	7.292	7.388	1.0000
15	8.434	8.538	1.0000
16	8.841	8.923	0.9987
17	10.580	10.689	1.0000
18	11.283	11.406	1.0000
19	11.761	11.844	1.0000
21	12.531	12.629	1.0000
20	12.632	12.709	1.0000
22	14.787	14.877	1.0000
23	17.568	17.651	1.0000
24	17.754	17.850	1.0000
27	23.010	23.095	1.0000
29	28.828	28.943	1.0000
30	43.197	43.314	1.0000

Colour	Class
(green)	TP
(yellow)	TTP
(red)	FP

	TN	TTN	FN
**VOC Label**	12, 25, 26, 28	11	-

S4.2.37 Table: Detailed evaluation of the results of the CNN-based system with VGG-8-1D analysis (Stage 2) of the testing clinical sample Test-08-BS02.

Test_08_BS02 (Sample ID 37)

VOC Label	Start detection sRT	End detection eRT	Confidence $\mathcal{T}$
1	3.063	3.210	1.0000
2	3.266	3.413	1.0000
3	3.751	3.842	1.0000
4	3.845	3.930	1.0000
5	4.234	4.447	1.0000
6	4.474	4.535	0.9914
7	5.370	5.468	1.0000
8	5.681	5.766	1.0000
9	5.768	5.861	1.0000
10	5.986	6.108	1.0000
11	6.148	6.267	1.0000
13	6.833	6.915	1.0000
14	7.295	7.393	1.0000
15	8.434	8.540	1.0000
16	8.843	8.939	1.0000
17	10.581	10.690	1.0000
18	11.287	11.409	1.0000
19	11.763	11.848	1.0000
21	12.535	12.633	1.0000
20	12.636	12.715	1.0000
22	14.788	14.881	1.0000
23	17.576	17.655	1.0000
24	17.759	17.852	1.0000
26	21.142	21.192	0.7765
27	23.014	23.099	1.0000
28	24.053	24.135	0.9972
29	28.823	28.951	1.0000
30	43.206	43.312	1.0000

Colour	Class
(green)	**TP**
(yellow)	**TTP**
(red)	**FP**

	TN	TTN	FN
**VOC Label**	12, 25	11	-

S4.2.38 Table: Detailed evaluation of the results of the CNN-based system with VGG-8-1D analysis (Stage 2) of the testing clinical sample Test-08-ES.

Test_08_ES (Sample ID 38)

VOC Label	Start detection sRT	End detection eRT	Confidence T
1	3.086	3.210	1.0000
2	3.287	3.386	0.9998
3	3.770	3.826	0.9951
4	3.829	3.946	1.0000
5	4.237	4.354	1.0000
6	4.357	4.521	1.0000
8	5.673	5.768	1.0000
9	5.771	5.861	1.0000
12	6.341	6.450	1.0000
13	6.837	6.912	0.9994
14	7.302	7.387	1.0000
15	8.429	8.533	1.0000
16	8.838	8.936	1.0000
17	10.577	10.689	1.0000
18	11.288	11.397	1.0000
19	11.758	11.845	1.0000
21	12.530	12.620	1.0000
20	12.622	12.705	0.9998
22	14.782	14.877	1.0000
23	17.573	17.653	1.0000
24	17.757	17.850	1.0000
25	18.226	18.338	1.0000
26	20.921	21.038	1.0000
27	23.013	23.098	1.0000
29	28.823	28.945	1.0000
30	43.214	43.301	0.9963

(green)	**TP**
(yellow)	**TTP**
(red)	**FP**

	TN	TTN	FN
**VOC Label**	7, 10, 11, 28	-	-

5 DenseNet-40-1D model – Results

S5.1 Table: Results of the testing clinical sample analysis with the CNN-based system with DenseNet-40-1D model. Results evaluated per each target VOC.

Label - the class label of VOC. *TP* - number of true positives detected per VOC. *TTP* - number of tentative true positives detected per VOC. *FP* - number of false positives detected per VOC. *TTN* - number of tentative true negatives detected per VOC. *FN* - number of false negatives detected per VOC. *TN* - number of true negatives detected per VOC (evaluation excluding *RT* position). *FP** - number of false positives detected per VOC (evaluation excluding *RT* position). *TTP** - number of tentative true positives detected per VOC (evaluation excluding *RT* position). *Sensitivity (expert)* - Sensitivity per VOC in relation to expert-derived ground truth benchmark; tentative true positives (TTP) are considered FP, tentative true negatives (TTN) are considered FN; sensitivity = TP/(TP+FN+TTN). *Sensitivity (system)* - Sensitivity per VOC in relation to system-derived correction benchmark; tentative true positives (TTP) are considered TP, tentative true negatives (TTN) are considered TN; sensitivity=(TP+TTP)/(TP+TTP+FN). *Specificity (expert)* - Specificity per VOC in relation to expert-derived ground truth benchmark; tentative true positives (TTP*) are considered FP*; specificity=TN/TN+FP*+TTP*. *Specificity (system)* - Specificity per VOC in relation to system-derived correction benchmark; tentative true positives (TTP*) are considered TP; specificity=TN/TN+FP*. *AP (expert)* - Average precision per VOC in relation to expert-derived ground truth benchmark. *AP (system)* - Average precision per VOC in relation to system-derived correction benchmark.

Label	TP	TTP	FP	TTN	FN	TN	FP*	TTP*	Sensitivity (expert)	Sensitivity (system)	Specificity (expert)	Specificity (system)	AP (expert)	AP (system)
1	20	18	0	0	0	0	0	18	1	1	0	-	0.6969	1
2	29	6	0	0	0	3	0	6	1	1	0.3333	1	0.9185	1
3	30	6	1	0	0	1	1	6	1	1	0.125	0.5	0.9925	0.9985
4	36	2	0	2	0	0	0	0	0.9474	1	-	-	0.9955	1
5	35	3	0	1	0	0	0	2	0.9722	1	0	-	0.9323	1
6	10	27	0	1	0	1	0	26	0.9091	1	0.037	1	0.7453	1
7	23	7	0	0	0	8	0	7	1	1	0.5333	1	0.9852	1
8	37	1	0	0	0	0	0	1	1	1	0	-	0.9744	1
9	38	0	0	0	0	0	0	0	1	1	-	-	1	1
10	30	7	0	0	0	1	0	7	1	1	0.125	1	0.9870	1
11	18	15	0	1	0	5	0	14	0.9474	1	0.2632	1	0.6717	1
12	22	11	0	1	2	3	0	10	0.88	0.9429	0.2308	1	0.9140	1
13	32	5	0	1	0	1	0	4	0.9697	1	0.2	1	0.9644	1
14	29	1	0	0	4	4	0	1	0.8788	0.8824	0.8	1	0.9989	1
15	17	10	0	1	5	7	0	9	0.7391	0.8438	0.4375	1	0.8225	1
16	19	3	4	1	3	10	3	2	0.8261	0.88	0.6667	0.7692	0.9839	0.9838
17	38	0	0	0	0	0	0	0	1	1	-	-	1	1
18	35	2	0	0	1	0	0	2	0.9722	0.9737	0	-	0.9490	1
19	26	5	0	0	0	7	0	5	1	1	0.5833	1	0.9143	1
20	35	0	0	0	2	1	0	0	0.9459	0.9459	1	1	1	1
21	20	17	0	6	1	0	0	11	0.7407	0.9737	0	-	0.9696	1
22	32	5	0	0	0	1	0	5	1	1	0.1667	1	0.9044	1
23	36	0	0	0	0	2	0	0	1	1	1	1	1	1
24	23	7	1	0	1	7	0	7	0.9583	0.9677	0.5	1	0.8926	1
25	7	4	0	0	0	26	0	4	1	1	0.8667	1	0.9107	1
26	21	8	0	0	1	8	0	8	0.9545	0.9667	0.5	1	0.9762	1
27	32	2	0	0	0	4	0	2	1	1	0.6667	1	0.9982	1
28	16	5	0	0	1	16	0	5	0.9412	0.9545	0.7619	1	0.8520	1
29	31	2	0	0	0	5	0	2	1	1	0.7143	1	0.9529	1
30	31	2	0	0	1	4	0	2	0.9688	0.9706	0.6667	1	0.9960	1
**TOTAL**	**808**	**181**	**6**	**15**	**22**	**125**	**4**	**166**	**0.9562**	**0.9782**	**0.4237**	**0.969**	**mAP**	
													**0.93**	**0.9994**

S5.1-S5.8 Figures: Precision against recall graphs for VOCs 1-8.

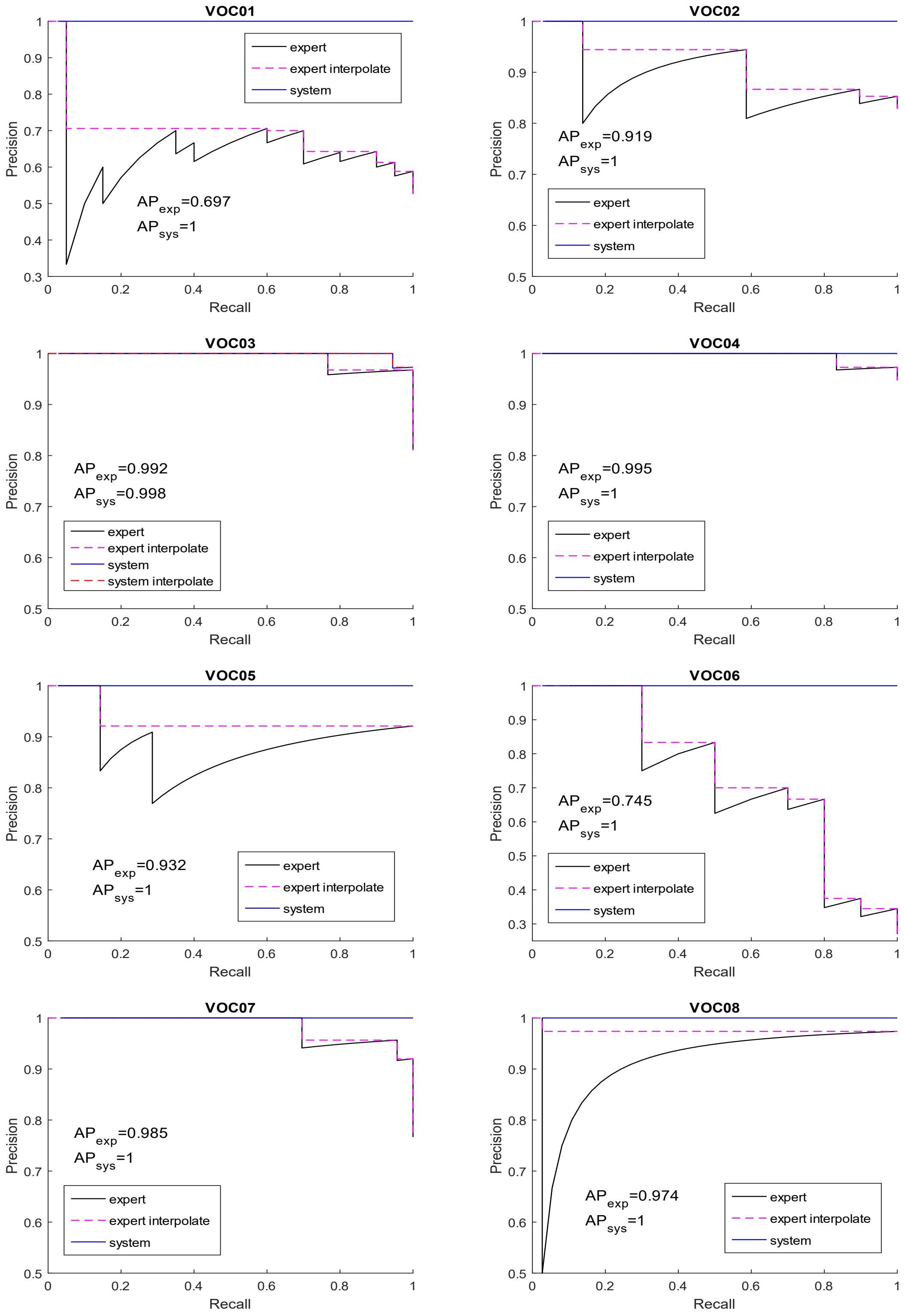

S5.9-S5.16 Figures: Precision against recall graphs for VOCs 9-16.

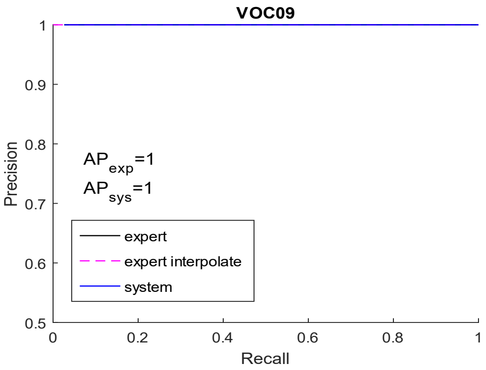

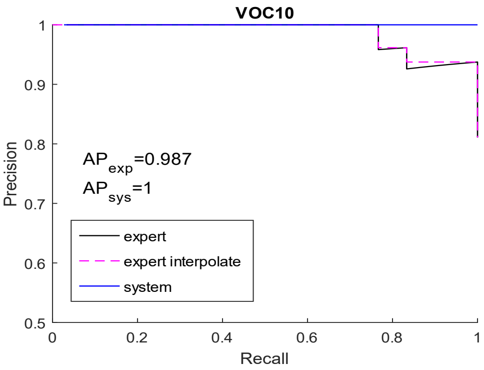

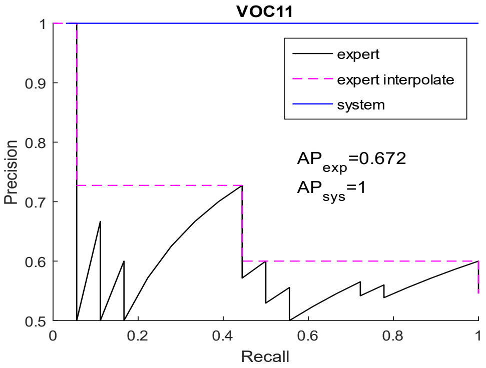

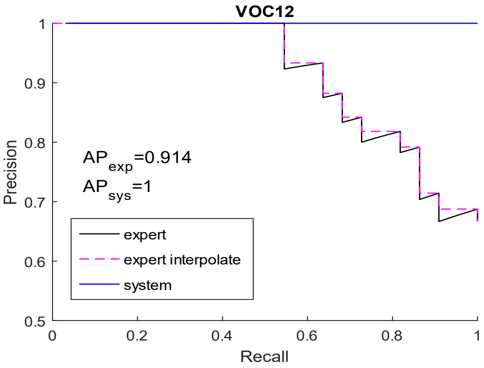

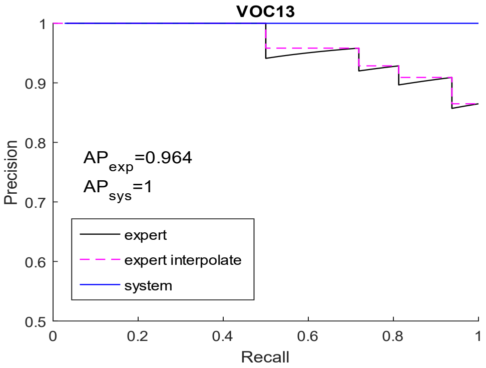

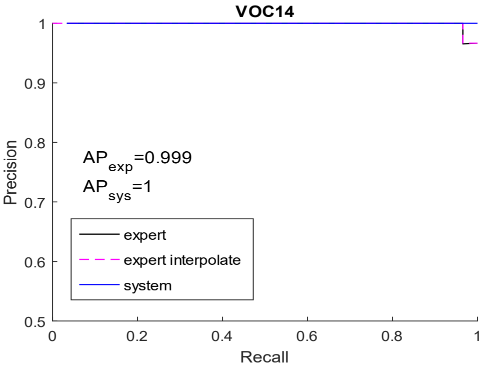

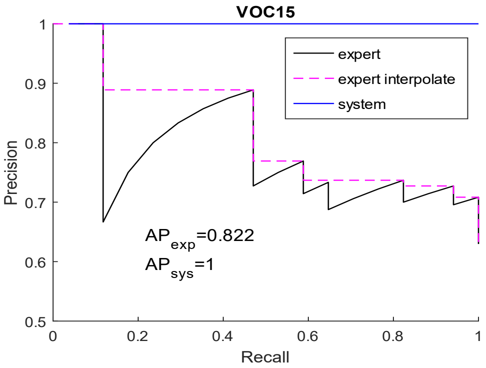

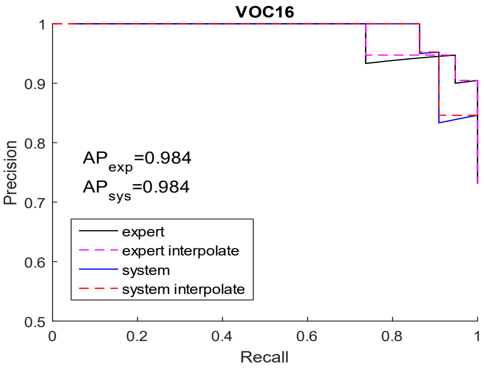

S5.17-S5.24 Figures: Precision against recall graphs for VOCs 17-24.

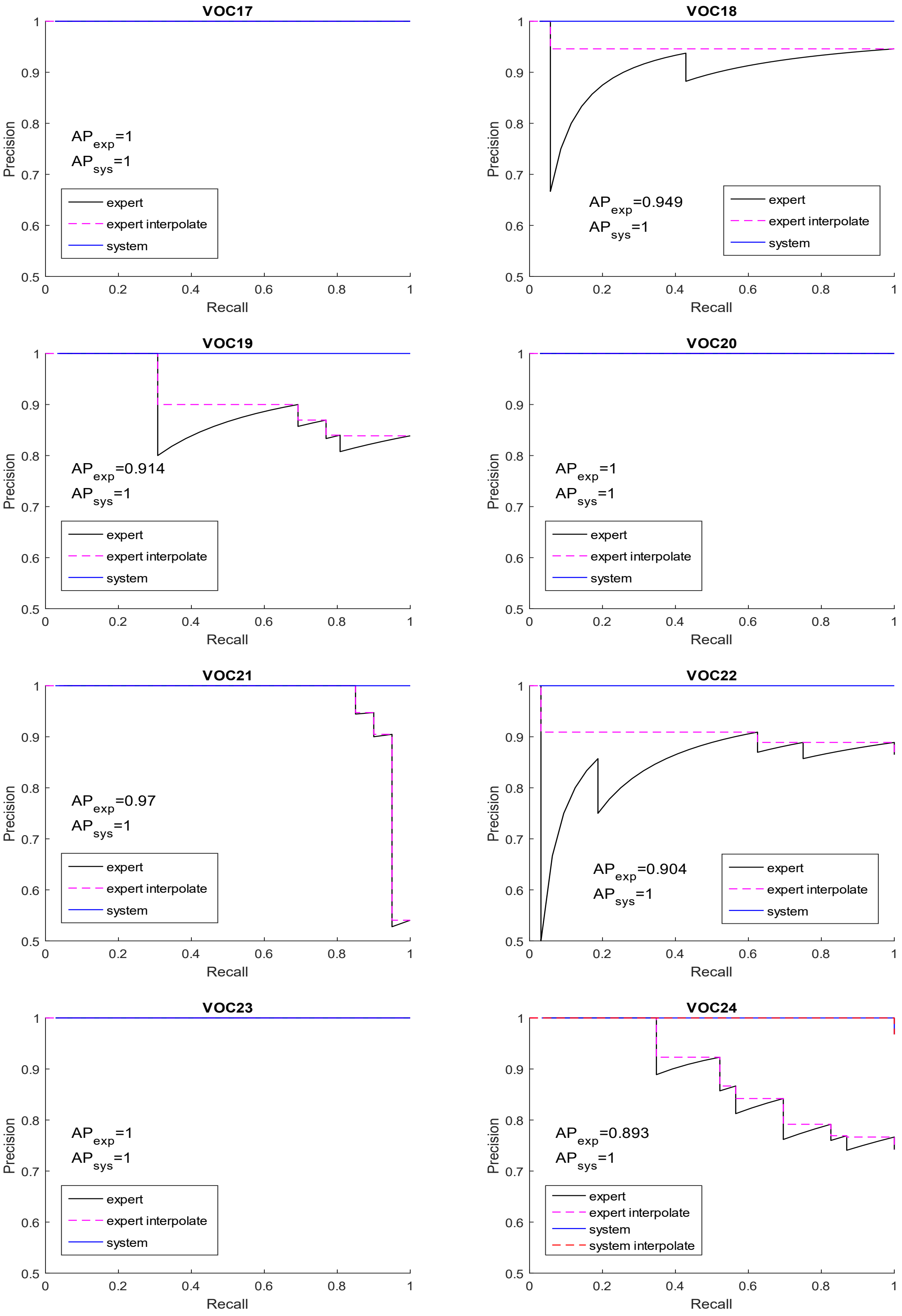

S5.25-S5.30 Figures: Precision against recall graphs for VOCs 25-30.

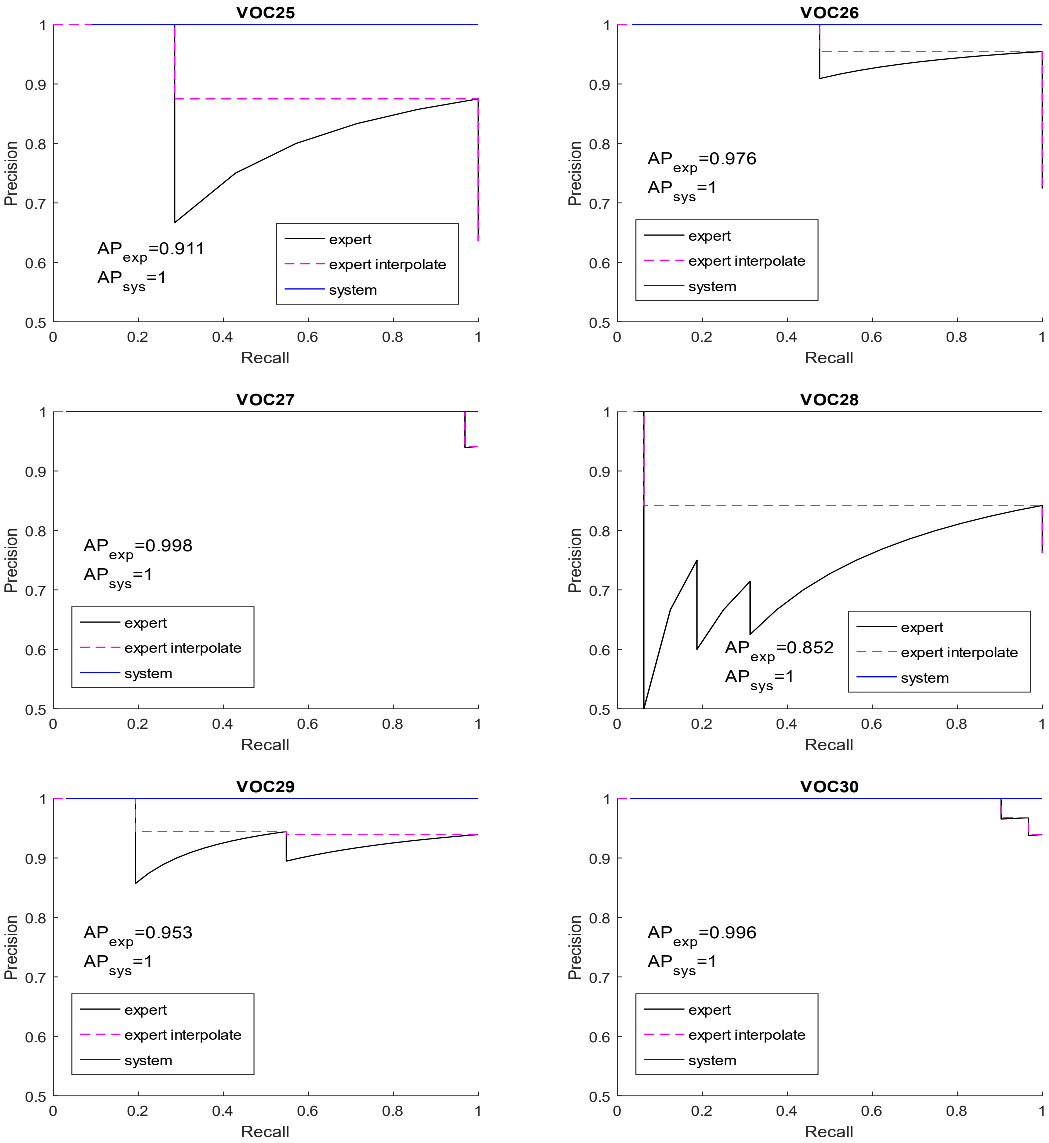

S5.2 Table: Results of the testing clinical sample analysis with the CNN-based system with DenseNet-40-1D model. Results evaluated per each testing sample.

Sample ID - number of a sample. *TP* - number of true positives detected per sample. *TTP* - number of tentative true positives detected per sample. *FP* - number of false positives detected per sample. *TTN* - number of tentative true negatives detected per sample. *FN* - number of false negatives detected per sample. *TN* - number of true negatives detected per sample (evaluation excluding RT position). *FP** - number of false positives detected per sample (evaluation excluding RT position). *TTP** - number of tentative true positives detected per sample (evaluation excluding RT position). *Sensitivity (expert)* - Sensitivity per sample in relation to expert-derived ground truth benchmark; tentative true positives (TTP) are considered FP, tentative true negatives (TTN) are considered FN; sensitivity = TP/(TP+FN+TTN). *Sensitivity (system)* - Sensitivity per sample in relation to system-derived correction benchmark; tentative true positives (TTP) are considered TP, tentative true negatives (TTN) are considered TN; sensitivity=(TP+TTP)/(TP+TTP+FN). *Specificity (expert)* - Specificity per sample in relation to expert-derived ground truth benchmark; tentative true positives (TTP*) are considered FP*; specificity=TN/TN+FP*+TTP*. *Specificity (system)* - Specificity per sample in relation to system-derived correction benchmark; tentative true positives (TTP*) are considered TP; specificity=TN/TN+FP*.

Sample ID	TP	TTP	FP	TTN	FN	TN	FP*	TTP*	Sensitivity (expert)	Sensitivity (system)	Specificity (expert)	Specificity (system)
1	26	3	0	1	0	1	0	2	0.963	1	0.3333	1
2	24	5	0	1	0	1	0	4	0.96	1	0.2	1
3	26	3	0	2	0	1	0	1	0.9286	1	0.5	1
4	27	1	0	0	0	2	0	1	1	1	0.6667	1
5	16	6	0	0	0	8	0	6	1	1	0.5714	1
6	23	3	0	1	1	3	0	2	0.92	0.963	0.6	1
7	25	2	0	0	2	1	0	2	0.9259	0.931	0.3333	1
8	23	3	0	0	2	2	0	3	0.92	0.9286	0.4	1
9	24	2	0	0	2	2	0	2	0.9231	0.9286	0.5	1
10	9	5	0	0	2	14	0	5	0.8182	0.875	0.7368	1
11	23	5	0	0	1	1	0	5	0.9583	0.9655	0.1667	1
12	24	5	0	1	0	1	0	4	0.96	1	0.2	1
13	22	7	0	0	0	1	0	7	1	1	0.125	1
14	25	3	0	0	0	2	0	3	1	1	0.4	1
15	24	4	1	0	1	1	0	4	0.96	0.9655	0.2	1
16	22	3	0	2	1	4	0	1	0.88	0.9615	0.8	1
17	22	5	0	2	0	3	0	3	0.9167	1	0.5	1
18	23	2	1	1	2	3	0	1	0.8846	0.9259	0.75	1
19	24	3	0	0	0	3	0	3	1	1	0.5	1
20	22	2	0	0	2	4	0	2	0.9167	0.9231	0.6667	1
21	17	7	0	0	1	5	0	7	0.9444	0.96	0.4167	1
22	24	6	0	1	0	0	0	5	0.96	1	0	-
23	22	6	0	0	0	2	0	6	1	1	0.25	1
24	17	6	0	0	0	7	0	6	1	1	0.5385	1
25	22	6	0	0	0	2	0	6	1	1	0.25	1
26	15	8	0	0	1	6	0	8	0.9375	0.9583	0.4286	1
27	19	5	0	1	0	6	0	4	0.95	1	0.6	1
28	16	7	0	0	0	7	0	7	1	1	0.5	1
29	16	8	0	1	0	6	0	7	0.9412	1	0.4615	1
30	9	10	0	0	2	9	0	10	0.8182	0.9048	0.4737	1
31	19	8	1	0	0	2	1	8	1	1	0.1818	0.6667
32	20	5	1	0	1	3	1	5	0.9524	0.9615	0.3333	0.75
33	24	3	0	1	1	2	0	2	0.9231	0.9643	0.5	1
34	25	5	0	0	0	0	0	5	1	1	0	-
35	24	4	0	0	0	2	0	4	1	1	0.3333	1
36	21	6	1	0	0	2	1	6	1	1	0.2222	0.6667
37	23	4	0	0	0	3	0	4	1	1	0.4286	1
38	21	5	1	0	0	3	1	5	1	1	0.3333	0.75
**TOTAL**	**808**	**181**	**6**	**15**	**22**	**125**	**4**	**166**	**0.9562**	**0.9782**	**0.4237**	**0.969**

6 ResNet-34-1D model – Results

S6.1 Table: Results of the testing clinical sample analysis with the CNN-based system with ResNet-34-1D model. Results evaluated per each target VOC.

Label - the class label of VOC. *TP* - number of true positives detected per VOC. *TTP* - number of tentative true positives detected per VOC. *FP* - number of false positives detected per VOC. *TTN* - number of tentative true negatives detected per VOC. *FN* - number of false negatives detected per VOC. *TN* - number of true negatives detected per VOC (evaluation excluding *RT* position). *FP** - number of false positives detected per VOC (evaluation excluding *RT* position). *TTP** - number of tentative true positives detected per VOC (evaluation excluding *RT* position). *Sensitivity (expert)* - Sensitivity per VOC in relation to expert-derived ground truth benchmark; tentative true positives (TTP) are considered FP, tentative true negatives (TTN) are considered FN; sensitivity = TP/(TP+FN+TTN). *Sensitivity (system)* - Sensitivity per VOC in relation to system-derived correction benchmark; tentative true positives (TTP) are considered TP, tentative true negatives (TTN) are considered TN; sensitivity=(TP+TTP)/(TP+TTP+FN). *Specificity (expert)* - Specificity per VOC in relation to expert-derived ground truth benchmark; tentative true positives (TTP*) are considered FP*; specificity=TN/TN+FP*+TTP*. *Specificity (system)* - Specificity per VOC in relation to system-derived correction benchmark; tentative true positives (TTP*) are considered TP; specificity=TN/TN+FP*. *AP (expert)* - Average precision per VOC in relation to expert-derived ground truth benchmark. *AP (system)* - Average precision per VOC in relation to system-derived correction benchmark.

Label	TP	TTP	FP	TTN	FN	TN	FP*	TTP*	Sensitivity (expert)	Sensitivity (system)	Specificity (expert)	Specificity (system)	AP (expert)	AP (system)
1	20	18	0	0	0	0	0	18	1	1	0	-	0.7242	1
2	29	9	0	0	0	0	0	9	1	1	0	-	0.92	1
3	30	7	0	0	0	1	0	7	1	1	0.125	1	0.976	1
4	38	0	0	0	0	0	0	0	1	1	-	-	1	1
5	35	3	0	1	0	0	0	2	0.9722	1	0	-	0.9211	1
6	10	27	0	1	0	1	0	26	0.9091	1	0.037	1	0.7421	1
7	22	5	1	0	1	9	1	5	0.9565	0.9643	0.6	0.9	0.9941	1
8	37	1	0	0	0	0	0	1	1	1	0	-	0.9737	1
9	38	0	0	0	0	0	0	0	1	1	-	-	1	1
10	29	8	0	1	0	1	0	7	0.9667	1	0.125	1	0.9695	1
11	15	16	1	2	2	5	0	14	0.7895	0.9394	0.2632	1	0.6941	0.994
12	21	10	0	1	3	4	0	9	0.84	0.9118	0.3077	1	0.9216	1
13	30	6	0	1	2	0	0	5	0.9091	0.9474	0	-	0.9448	1
14	29	1	0	0	4	4	0	1	0.8788	0.8824	0.8	1	0.9977	1
15	18	10	0	1	4	6	0	9	0.7826	0.875	0.4	1	0.7106	1
16	18	4	6	1	4	8	4	3	0.7826	0.8462	0.5333	0.6667	0.9699	0.9512
17	38	0	0	0	0	0	0	0	1	1	-	-	1	1
18	36	2	0	0	0	0	0	2	1	1	0	-	0.9591	1
19	26	6	0	0	0	6	0	6	1	1	0.5	1	0.9035	1
20	35	0	0	0	2	1	0	0	0.9459	0.9459	1	1	1	1
21	21	16	0	5	1	0	0	11	0.7778	0.9737	0	-	0.9118	1
22	32	5	0	0	0	1	0	5	1	1	0.1667	1	0.9584	1
23	36	0	0	0	0	2	0	0	1	1	1	1	1	1
24	23	7	0	0	1	7	0	7	0.9583	0.9677	0.5	1	0.8597	1
25	7	5	1	0	0	25	1	5	1	1	0.8065	0.9615	0.9571	0.9936
26	21	5	0	0	1	11	0	5	0.9545	0.963	0.6875	1	0.9868	1
27	32	4	0	0	0	2	0	4	1	1	0.3333	1	0.9962	1
28	16	6	0	0	1	15	0	6	0.9412	0.9565	0.7143	1	0.8889	1
29	31	3	0	0	0	4	0	3	1	1	0.5714	1	0.9692	1
30	31	3	0	0	1	3	0	3	0.9688	0.9714	0.5	1	0.997	1
**TOTAL**	**804**	**187**	**9**	**14**	**27**	**116**	**6**	**173**	**0.9515**	**0.9735**	**0.3932**	**0.9508**	**mAP**	
													**0.9282**	**0.998**

S6.1-S6.8 Figures: Precision against recall graphs for VOCs 1-8.

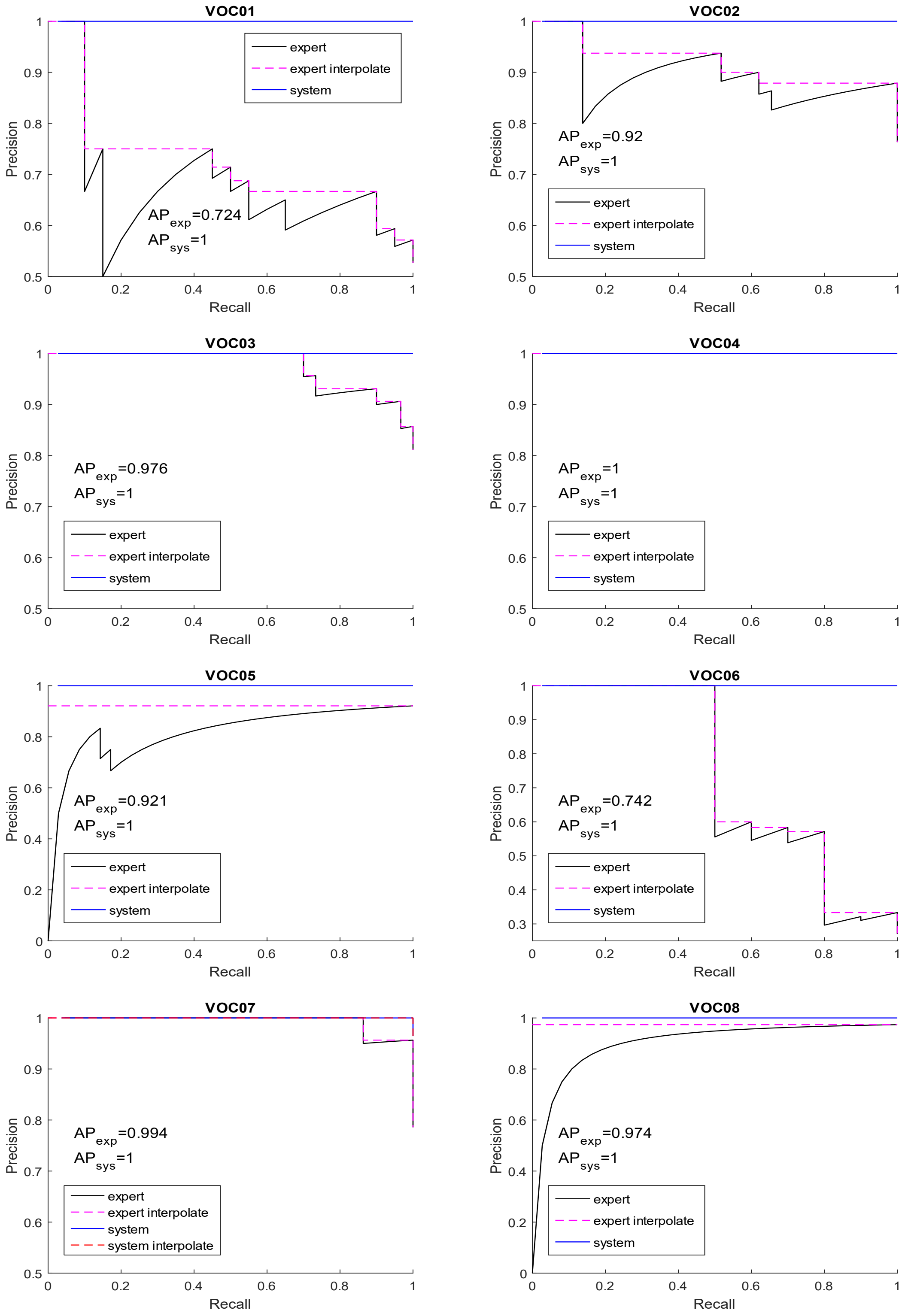

S6.9-S6.16 Figures: Precision against recall graphs for VOCs 9-16.

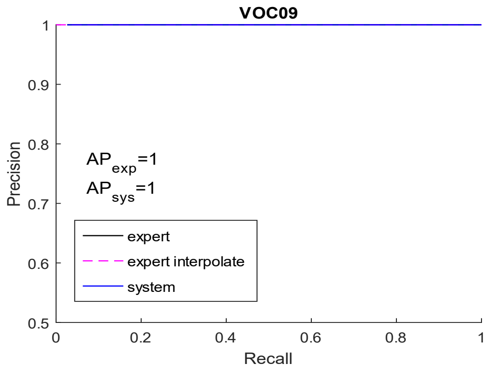

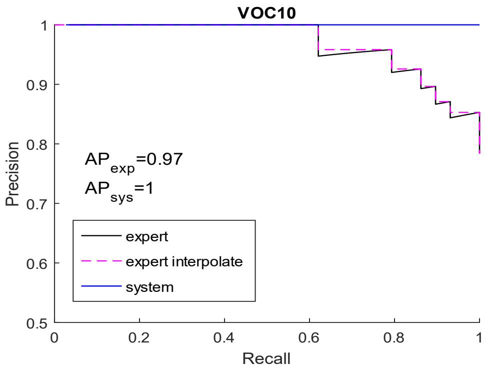

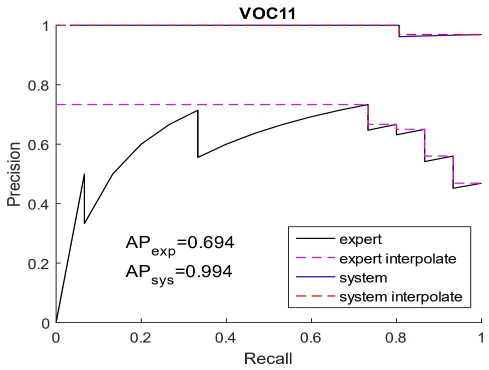

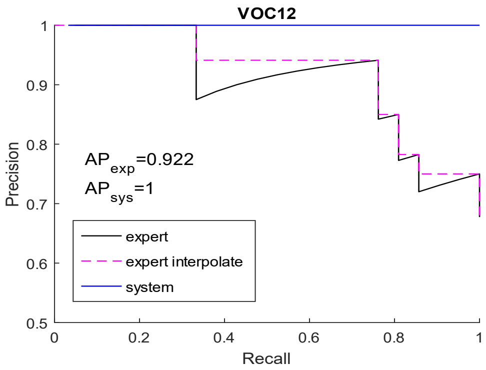

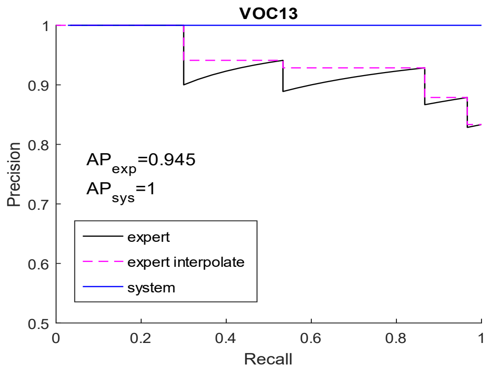

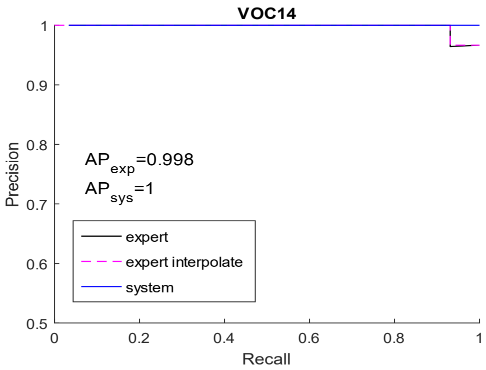

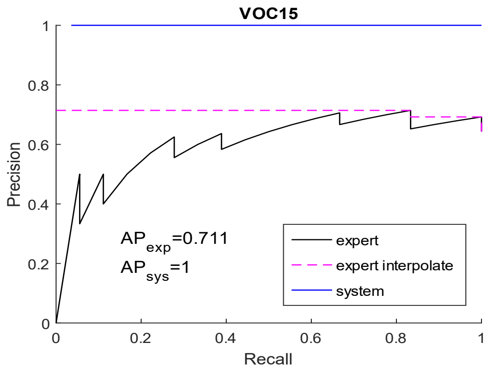

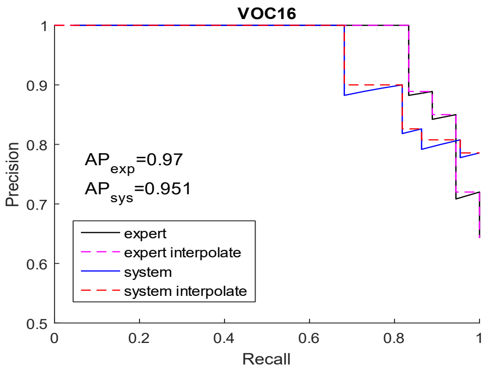

S6.17-S6.24 Figures: Precision against recall graphs for VOCs 17-24.

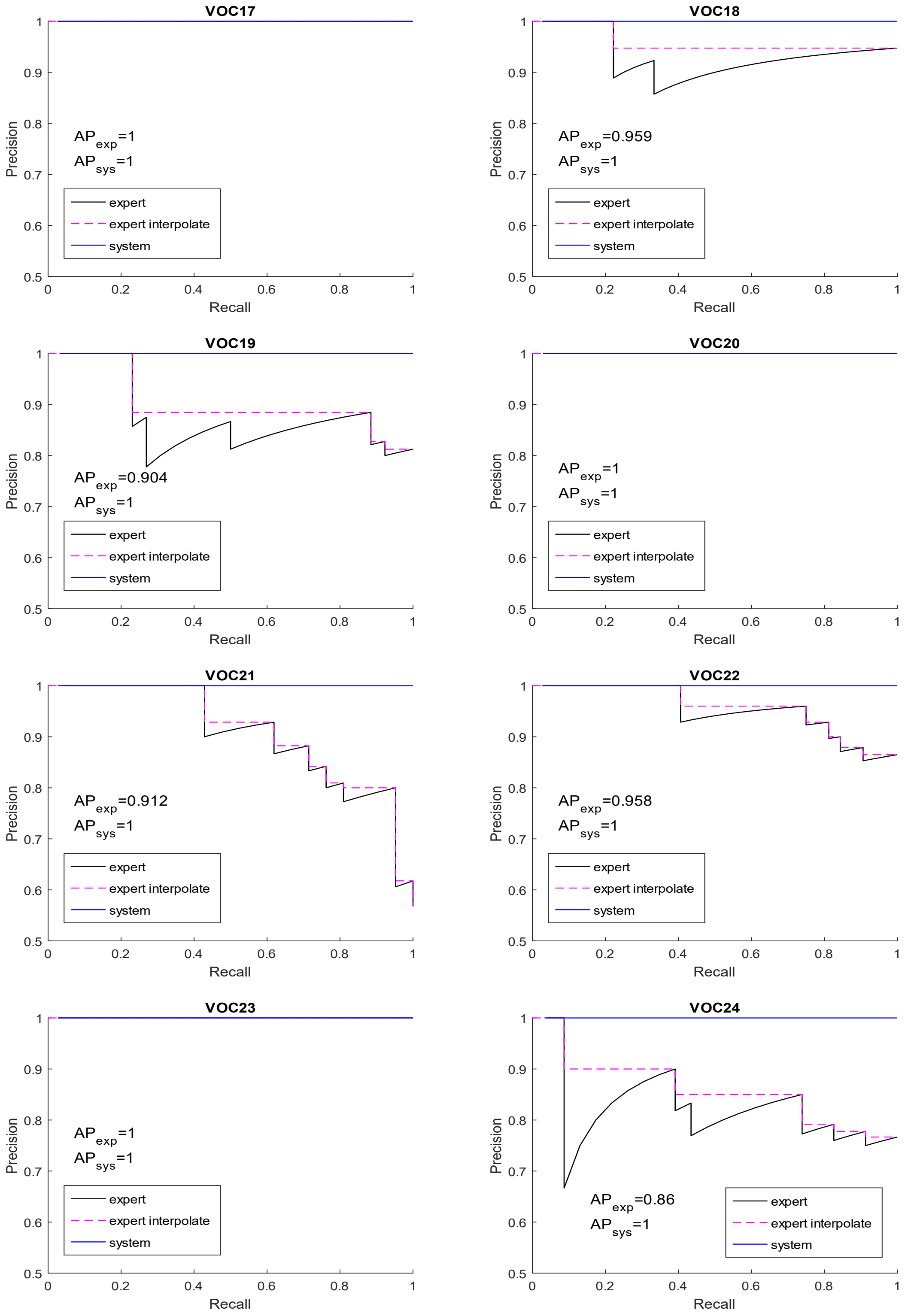

S6.25-S6.30 Figures: Precision against recall graphs for VOCs 25-30.

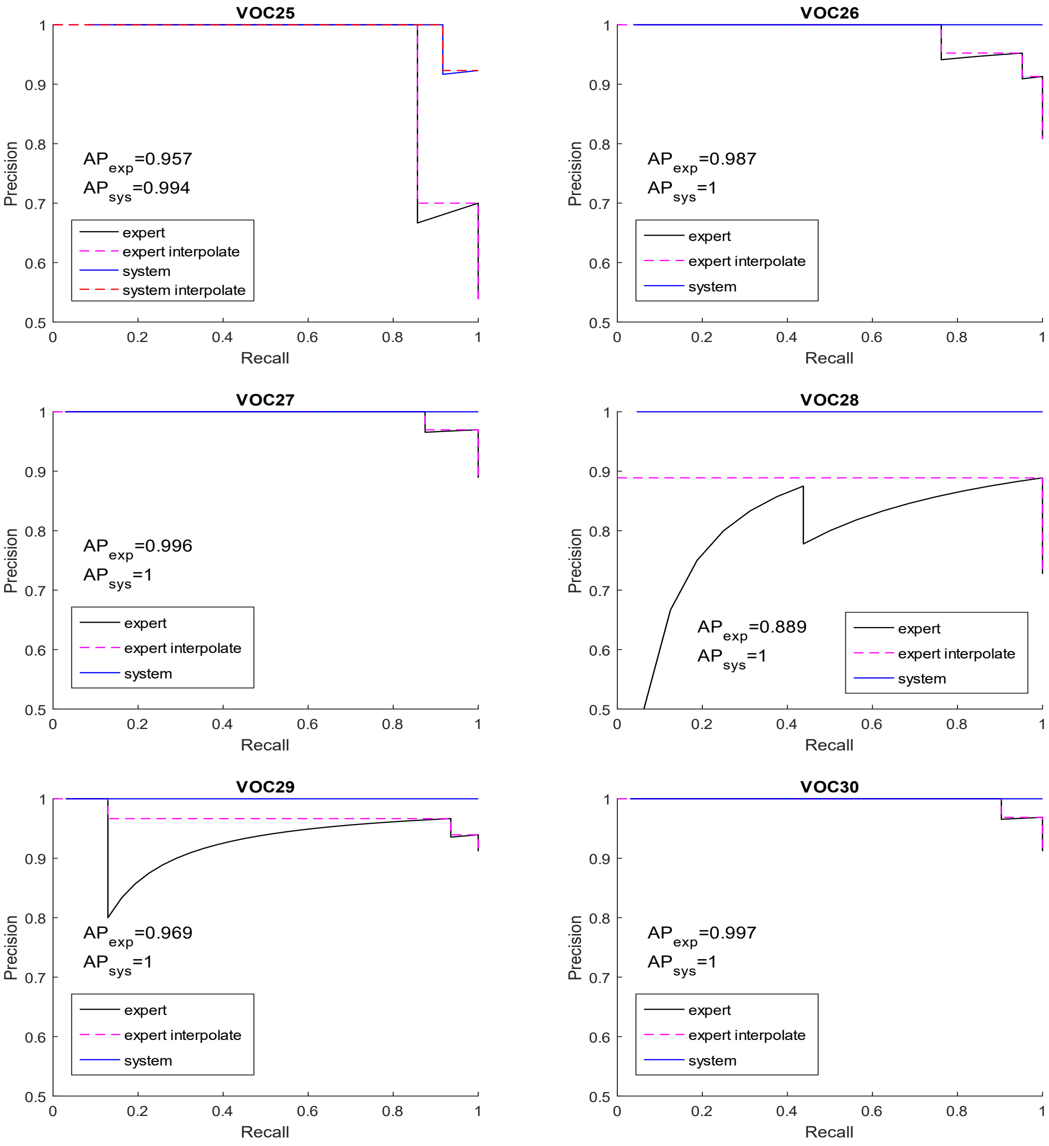

S6.2 Table: Results of the testing clinical sample analysis with the CNN-based system with ResNet-34-1D model. Results evaluated per each testing sample.

Sample ID - number of a sample. *TP* - number of true positives detected per sample. *TTP* - number of tentative true positives detected per sample. *FP* - number of false positives detected per sample. *TTN* - number of tentative true negatives detected per sample. *FN* - number of false negatives detected per sample. *TN* - number of true negatives detected per sample (evaluation excluding *RT* position). *FP** - number of false positives detected per sample (evaluation excluding *RT* position). *TTP** - number of tentative true positives detected per sample (evaluation excluding *RT* position). *Sensitivity (expert)* - Sensitivity per sample in relation to expert-derived ground truth benchmark; tentative true positives (TTP) are considered FP, tentative true negatives (TTN) are considered FN; sensitivity = TP/(TP+FN+TTN). *Sensitivity (system)* - Sensitivity per sample in relation to system-derived correction benchmark; tentative true positives (TTP) are considered TP, tentative true negatives (TTN) are considered TN; sensitivity=(TP+TTP)/(TP+TTP+FN). *Specificity (expert)* - Specificity per sample in relation to expert-derived ground truth benchmark; tentative true positives (TTP*) are considered FP*; specificity=TN/TN+FP*+TTP*. *Specificity (system)* - Specificity per sample in relation to system-derived correction benchmark; tentative true positives (TTP*) are considered TP; specificity=TN/TN+FP*.

Sample ID	TP	TTP	FP	TTN	FN	TN	FP*	TTP*	Sensitivity (expert)	Sensitivity (system)	Specificity (expert)	Specificity (system)
1	27	2	0	0	0	1	0	2	1	1	0.3333	1
2	24	5	0	1	0	1	0	4	0.96	1	0.2	1
3	26	3	0	2	0	1	0	1	0.9286	1	0.5	1
4	27	1	0	0	0	2	0	1	1	1	0.6667	1
5	15	7	0	0	1	7	0	7	0.9375	0.9565	0.5	1
6	23	4	0	1	1	2	0	3	0.92	0.9643	0.4	1
7	24	2	0	0	3	1	0	2	0.8889	0.8966	0.3333	1
8	23	3	0	0	2	2	0	3	0.92	0.9286	0.4	1
9	24	2	0	0	2	2	0	2	0.9231	0.9286	0.5	1
10	9	7	0	0	2	12	0	7	0.8182	0.8889	0.6316	1
11	24	5	0	0	0	1	0	5	1	1	0.1667	1
12	24	5	0	1	0	1	0	4	0.96	1	0.2	1
13	22	6	1	0	0	1	1	6	1	1	0.125	0.5
14	24	3	1	0	1	2	0	3	0.96	0.9643	0.4	1
15	24	4	1	0	1	1	0	4	0.96	0.9655	0.2	1
16	19	5	1	3	3	3	0	2	0.76	0.8889	0.6	1
17	22	5	1	2	0	2	1	3	0.9167	1	0.3333	0.6667
18	23	2	0	1	2	3	0	1	0.8846	0.9259	0.75	1
19	23	3	0	1	0	4	0	2	0.9583	1	0.6667	1
20	22	2	0	0	2	4	0	2	0.9167	0.9231	0.6667	1
21	17	6	0	0	1	6	0	6	0.9444	0.9583	0.5	1
22	24	5	0	0	1	0	0	5	0.96	0.9667	0	-
23	22	5	1	0	0	2	1	5	1	1	0.25	0.6667
24	17	5	0	0	0	8	0	5	1	1	0.6154	1
25	22	6	0	0	0	2	0	6	1	1	0.25	1
26	16	8	0	0	0	6	0	8	1	1	0.4286	1
27	19	7	0	1	0	4	0	6	0.95	1	0.4	1
28	15	9	0	0	1	5	0	9	0.9375	0.96	0.3571	1
29	16	7	0	0	1	6	0	7	0.9412	0.9583	0.4615	1
30	10	13	0	0	1	6	0	13	0.9091	0.9583	0.3158	1
31	19	6	1	0	0	4	1	6	1	1	0.3636	0.8
32	20	5	1	0	1	3	1	5	0.9524	0.9615	0.3333	0.75
33	25	4	0	1	0	1	0	3	0.9615	1	0.25	1
34	24	5	0	0	1	0	0	5	0.96	0.9667	0	-
35	24	4	0	0	0	2	0	4	1	1	0.3333	1
36	21	5	1	0	0	3	1	5	1	1	0.3333	0.75
37	23	4	0	0	0	3	0	4	1	1	0.4286	1
38	21	7	0	0	0	2	0	7	1	1	0.2222	1
**TOTAL**	**804**	**187**	**9**	**14**	**27**	**116**	**6**	**173**	**0.9515**	**0.9735**	**0.3932**	**0.9508**

7 Intersection of the models – Results

S7.1 Table: Intersection of the results of the testing clinical sample analysis with the CNN-based system with all tested models (i.e. detections consistent in terms of label and RT location among all the models). Results evaluated per each target VOC.

Label - the class label of VOC. *TP* - number of true positives detected per VOC. *TTP* - number of tentative true positives detected per VOC. *FP* - number of false positives detected per VOC. *TTN* - number of tentative true negatives detected per VOC. *FN* - number of false negatives detected per VOC. *TN* - number of true negatives detected per VOC (evaluation excluding RT position). *FP** - number of false positives detected per VOC (evaluation excluding RT position). *TTP** - number of tentative true positives detected per VOC (evaluation excluding RT position). *Sensitivity (expert)* - Sensitivity per VOC in relation to expert-derived ground truth benchmark; tentative true positives (TTP) are considered FP, tentative true negatives (TTN) are considered FN; sensitivity = TP/(TP+FN+TTN). *Sensitivity (system)* - Sensitivity per VOC in relation to system-derived correction benchmark; tentative true positives (TTP) are considered TP, tentative true negatives (TTN) are considered TN; sensitivity=(TP+TTP)/(TP+TTP+FN). *Specificity (expert)* - Specificity per VOC in relation to expert-derived ground truth benchmark; tentative true positives (TTP*) are considered FP*; specificity=TN/TN+FP*+TTP*. *Specificity (system)* - Specificity per VOC in relation to system-derived correction benchmark; tentative true positives (TTP*) are considered TP; specificity=TN/TN+FP*. *AP (expert)* - Average precision per VOC in relation to expert-derived ground truth benchmark. *AP (system)* - Average precision per VOC in relation to system-derived correction benchmark.

Label	TP	TTP	FP	TTN	FN	TN	FP*	TTP*	Sensitivity (expert)	Sensitivity (system)	Specificity (expert)	Specificity (system)	AP (expert)	AP (system)
1	20	18	0	0	0	0	0	18	1	1	-	-	0.7436	1
2	29	6	0	0	0	3	0	6	1	1	0.3333	-	0.9173	1
3	30	6	0	0	0	2	0	6	1	1	0.25	1	0.9925	1
4	36	0	0	0	2	0	0	0	0.9474	0.9474	-	-	1	1
5	35	3	0	1	0	0	0	2	0.9722	1	-	-	0.9233	1
6	10	27	0	1	0	1	0	26	0.9091	1	0.037	1	0.8035	1
7	22	3	0	0	1	12	0	3	0.9565	0.9615	0.8	1	0.998	1
8	37	1	0	0	0	0	0	1	1	1	-	-	0.9737	1
9	38	0	0	0	0	0	0	0	1	1	-	-	1	1
10	29	4	0	0	1	4	0	4	0.9667	0.9706	0.5	1	0.992	1
11	12	7	0	0	7	12	0	7	0.6316	0.7308	0.6316	1	0.67	1
12	20	7	0	1	4	7	0	6	0.8	0.871	0.5385	1	0.9357	1
13	30	4	0	1	2	2	0	3	0.9091	0.9444	0.4	-	0.9518	1
14	28	1	0	0	5	4	0	1	0.8485	0.8529	0.8	1	0.9988	1
15	17	10	0	1	5	6	0	9	0.7391	0.8438	0.4	1	0.7873	1
16	18	1	1	0	3	15	0	1	0.8571	0.8636	0.9375	1	0.9971	0.9947
17	38	0	0	0	0	0	0	0	1	1	-	-	1	1
18	31	2	0	0	5	0	0	2	0.8611	0.8684	-	-	0.9511	1
19	26	4	0	0	0	9	0	4	1	1	0.6923	1	0.9108	1
20	35	0	0	0	2	1	0	0	0.9459	0.9459	1	1	1	1
21	20	5	0	3	4	9	0	2	0.7407	0.8621	0.8182	-	0.9808	1
22	32	5	0	0	0	1	0	5	1	1	0.1667	1	0.9416	1
23	36	0	0	0	0	2	0	0	1	1	1	1	1	1
24	23	6	0	0	2	7	0	6	0.92	0.9355	0.5385	1	0.8964	1
25	7	3	0	0	0	28	0	3	1	1	0.9032	1	0.9821	1
26	19	5	0	0	3	11	0	5	0.8636	0.8889	0.6875	1	0.9947	1
27	32	2	0	0	0	4	0	2	1	1	0.6667	1	0.9991	1
28	16	4	0	0	1	17	0	4	0.9412	0.9524	0.8095	1	0.8889	1
29	31	2	0	0	0	5	0	2	1	1	0.7143	1	0.9674	1
30	30	2	0	0	2	3	0	2	0.9375	0.9412	0.6	1	0.9978	1
**TOTAL**	**787**	**138**	**1**	**8**	**49**	**165**	**0**	**130**	**0.9325**	**0.9497**	**0.5593**	**1**	**mAP**	
													**0.9398**	**1**

S7.1-S7.8 Figures: Precision against recall graphs for VOCs 1-8.

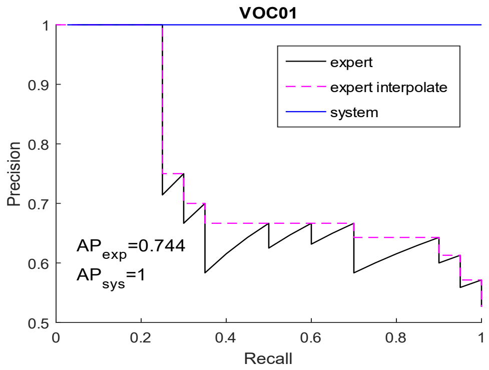

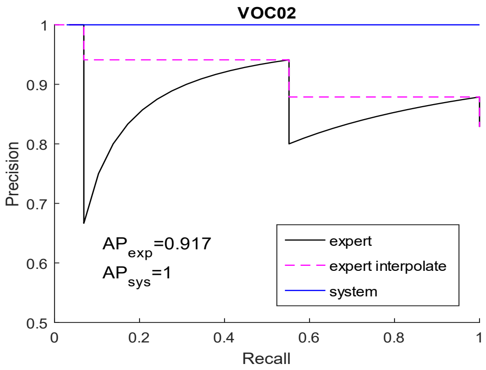

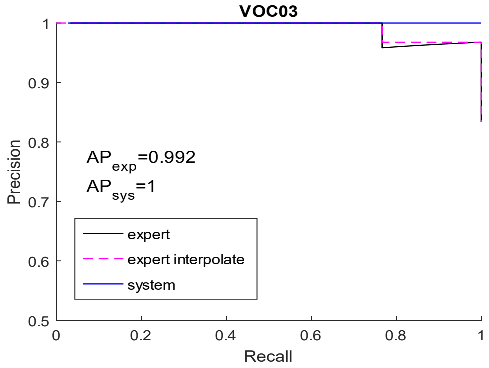

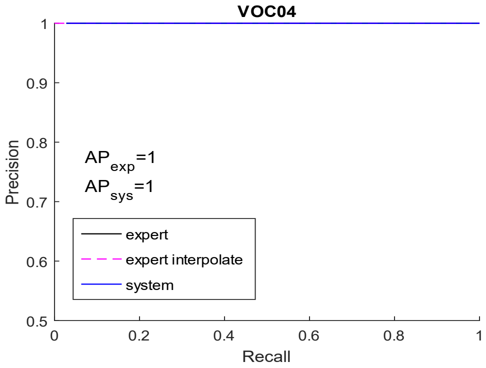

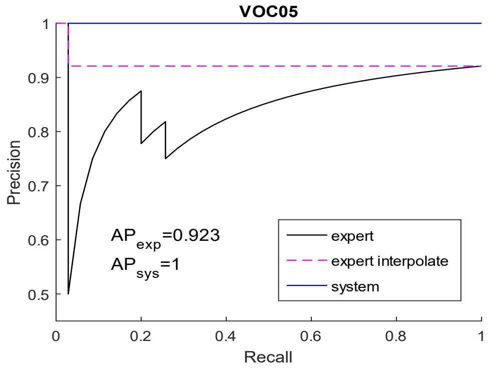

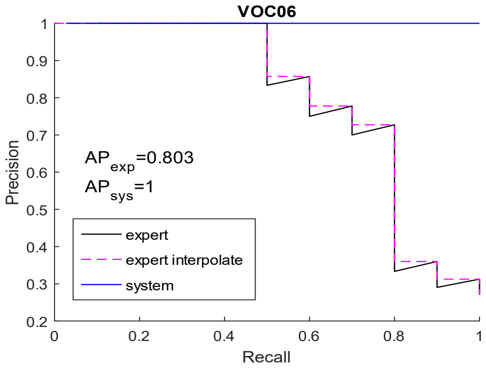

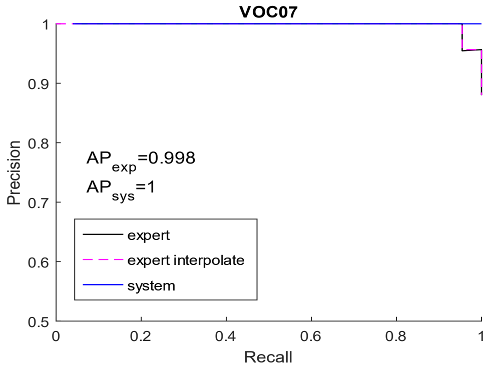

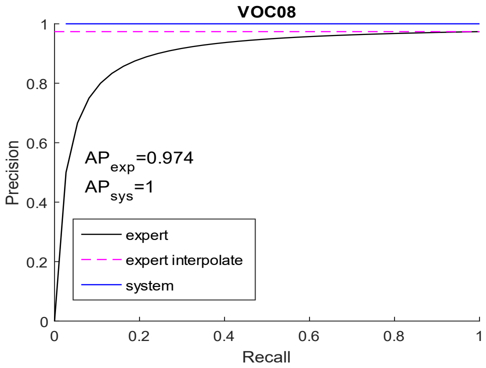

S7.9-S7.16 Figures: Precision against recall graphs for VOCs 9-16.

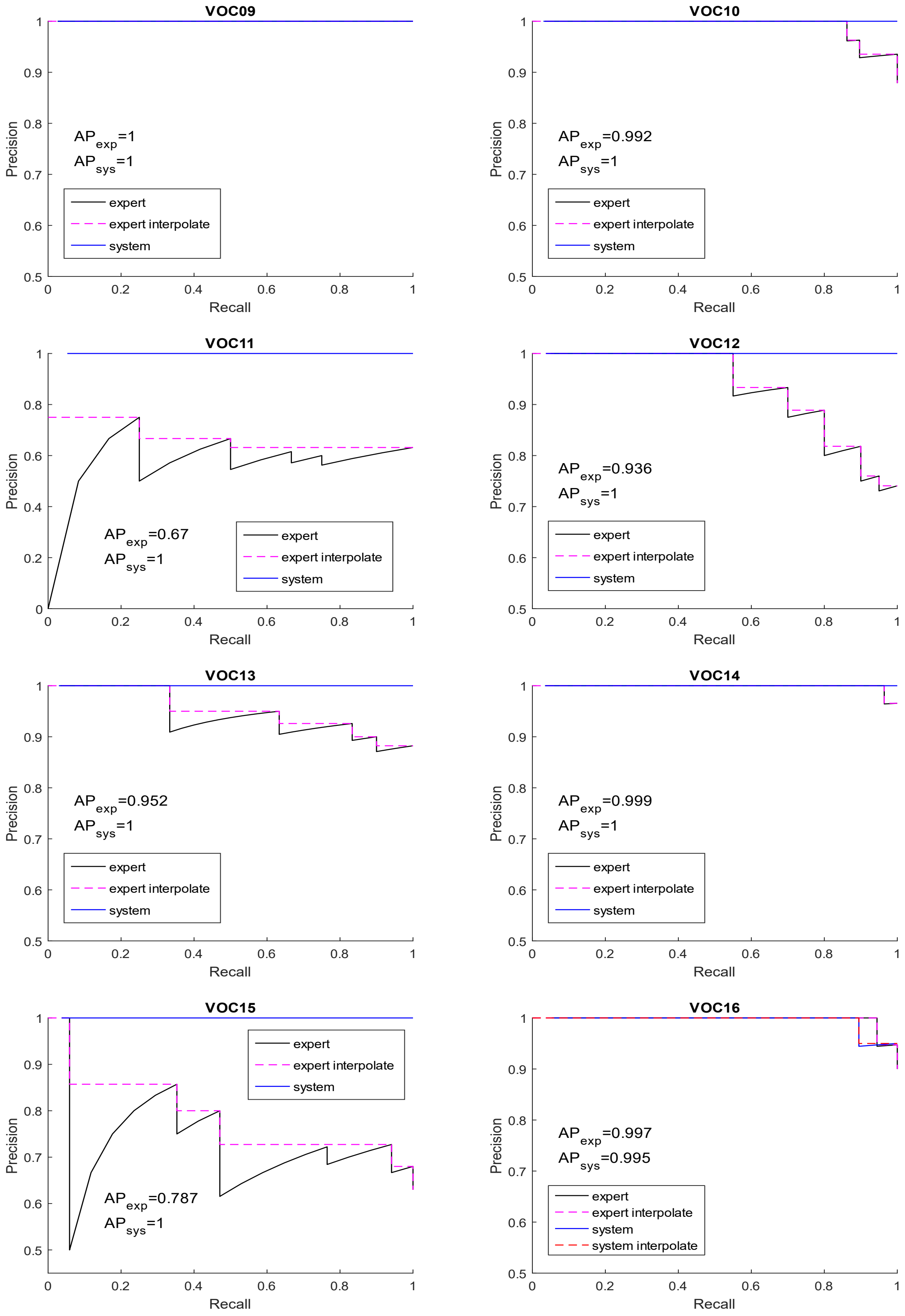

S7.17-S7.24 Figures: Precision against recall graphs for VOCs 17-24.

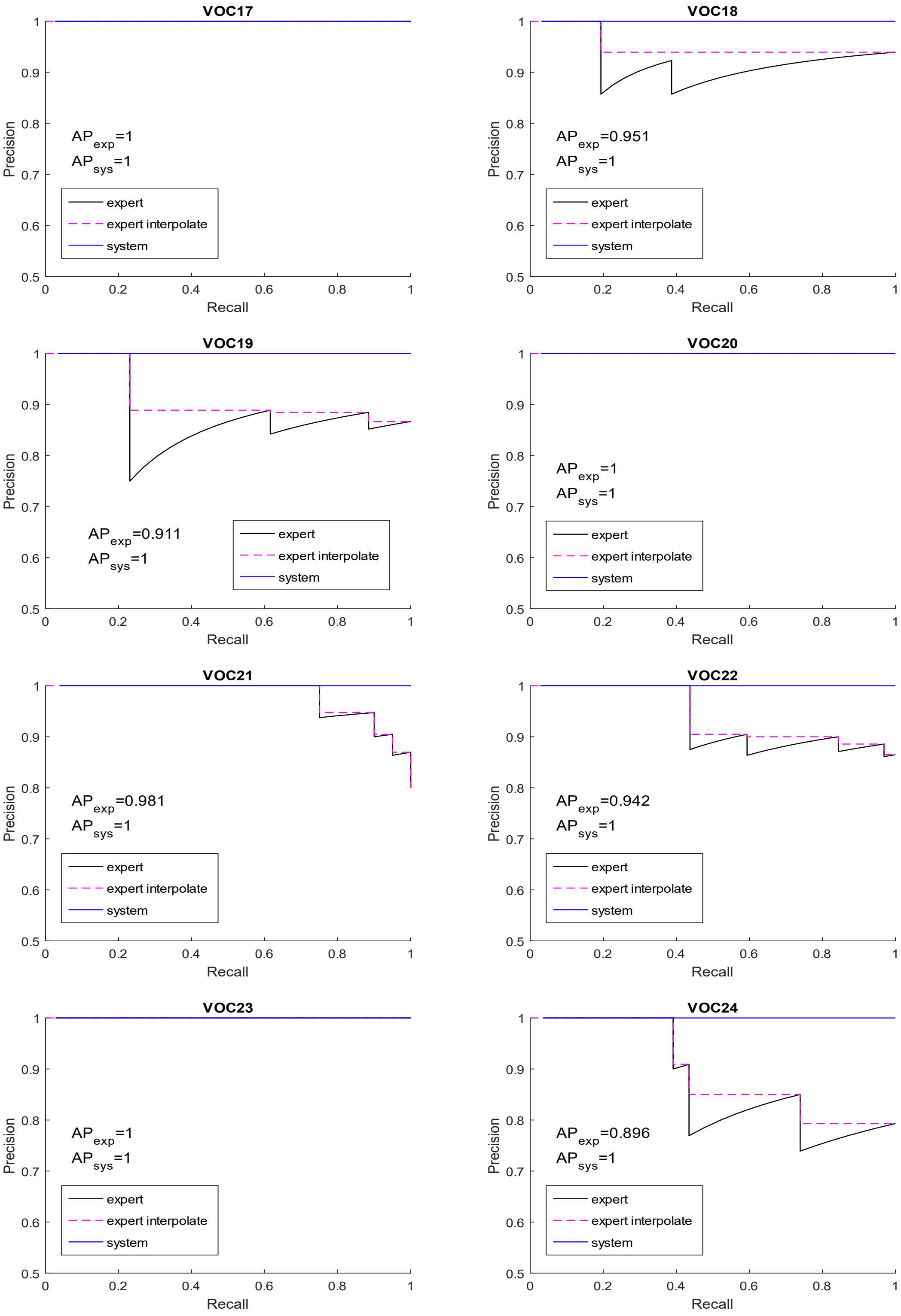

S7.25-S7.30 Figures: Precision against recall graphs for VOCs 25-30.

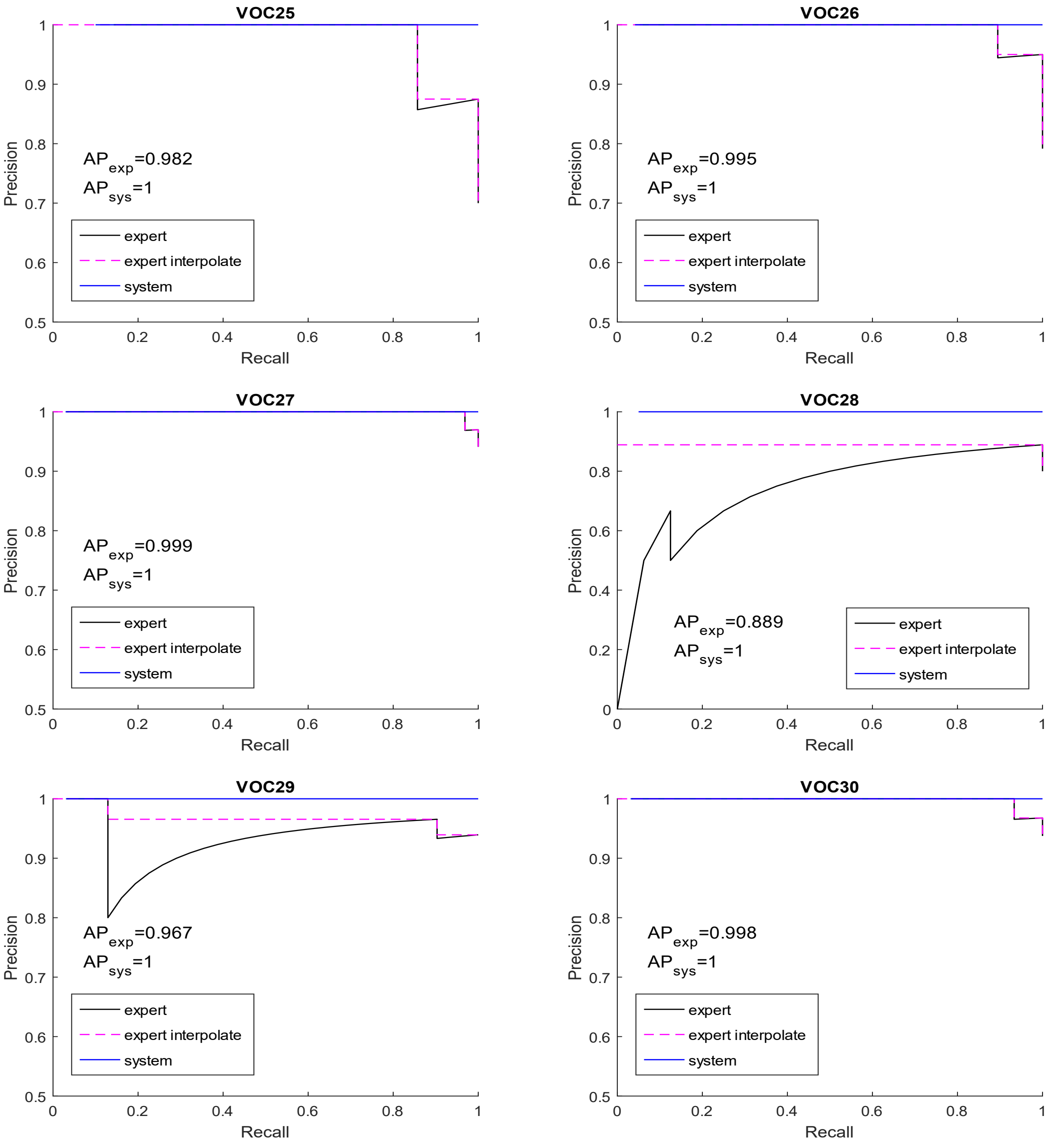

S7.2 Table: Intersection of the results of the testing clinical sample analysis with the CNN-based system with all tested models (i.e. detections consistent in terms of label and *RT* location among all the models). Results evaluated per each testing sample.

Label - the class label of VOC. *TP* - number of true positives detected per VOC. *TTP* - number of tentative true positives detected per VOC. *FP* - number of false positives detected per VOC. *TTN* - number of tentative true negatives detected per VOC. *FN* - number of false negatives detected per VOC. *TN* - number of true negatives detected per VOC (evaluation excluding *RT* position). *FP** - number of false positives detected per VOC (evaluation excluding *RT* position). *TTP** - number of tentative true positives detected per VOC (evaluation excluding *RT* position). *Sensitivity (expert)* - Sensitivity per VOC in relation to expert-derived ground truth benchmark; tentative true positives (TTP) are considered FP, tentative true negatives (TTN) are considered FN; sensitivity = TP/(TP+FN+TTN). *Sensitivity (system)* - Sensitivity per VOC in relation to system-derived correction benchmark; tentative true positives (TTP) are considered TP, tentative true negatives (TTN) are considered TN; sensitivity=(TP+TTP)/(TP+TTP+FN). *Specificity (expert)* - Specificity per VOC in relation to expert-derived ground truth benchmark; tentative true positives (TTP*) are considered FP*; specificity=TN/TN+FP*+TTP*. *Specificity (system)* - Specificity per VOC in relation to system-derived correction benchmark; tentative true positives (TTP*) are considered TP; specificity=TN/TN+FP*.

Sample ID	TP	TTP	FP	TTN	FN	TN	FP*	TTP*	Sensitivity (expert)	Sensitivity (system)	Specificity (expert)	Specificity (system)
1	26	2	0	0	1	1	0	2	1	1	0.3333	1
2	24	4	0	0	1	1	0	4	1	1	0.2	1
3	26	2	0	1	1	1	0	1	0.963	0.9655	0.5	1
4	27	0	0	0	0	3	0	0	1	1	-	-
5	15	4	0	0	1	10	0	4	1	1	0.2	1
6	23	2	0	1	1	4	0	1	0.9583	0.9615	0.5	1
7	24	1	0	0	3	2	0	1	1	1	0.75	1
8	23	2	0	0	2	3	0	2	1	1	0.5	1
9	24	1	0	0	2	3	0	1	1	1	0.6667	1
10	9	5	0	0	2	14	0	5	1	1	0.2857	1
11	23	4	0	0	1	2	0	4	1	1	0.2	1
12	24	4	0	1	0	2	0	3	0.96	0.9655	-	-
13	22	5	0	0	0	3	0	5	1	1	-	-
14	24	2	0	0	1	3	0	2	1	1	0.3333	1
15	24	3	1	0	0	2	0	3	0.96	1	-	-
16	18	2	0	1	6	4	0	1	0.9474	0.9524	0.8571	1
17	21	5	0	2	1	3	0	3	0.913	0.9286	0.25	1
18	22	2	0	1	3	3	0	1	0.9565	0.96	0.75	1
19	22	2	0	0	2	4	0	2	1	1	0.5	1
20	21	2	0	0	3	4	0	2	1	1	0.6	1
21	16	4	0	0	2	8	0	4	1	1	0.3333	1
22	23	4	0	0	2	1	0	4	1	1	0.3333	-
23	21	4	0	0	1	4	0	4	1	1	0.2	1
24	17	5	0	0	0	8	0	5	1	1	-	-
25	22	4	0	0	0	4	0	4	1	1	-	-
26	15	6	0	0	1	8	0	6	1	1	0.1429	1
27	19	5	0	1	0	6	0	4	0.95	0.96	-	-
28	15	7	0	0	1	7	0	7	1	1	0.125	1
29	16	7	0	0	1	6	0	7	1	1	0.125	1
30	9	10	0	0	2	9	0	10	1	1	0.1667	1
31	19	4	0	0	0	7	0	4	1	1	-	-
32	20	4	0	0	1	5	0	4	1	1	0.2	1
33	23	2	0	0	3	2	0	2	1	1	0.6	1
34	23	4	0	0	2	1	0	4	1	1	0.3333	-
35	24	3	0	0	0	3	0	3	1	1	-	-
36	20	4	0	0	1	5	0	4	1	1	0.2	1
37	22	3	0	0	1	4	0	3	1	1	0.25	1
38	21	4	0	0	0	5	0	4	1	1	-	-
**TOTAL**	**787**	**138**	**1**	**8**	**49**	**165**	**0**	**130**	**0.9325**	**0.9497**	**0.5593**	**1**

8 Learning curve (VGG-8-1D model)

S8 Figure: Learning curve plot of the training error and test error in dependence on the sample size of the training data for VGG-8-1D model. The VOC dataset has been extracted and fully-augmented from the GC-MS samples derived from 1 participant (1, 2 and all samples), 3, 6, 8, 11, 14 and all 17 participants from the training dataset.

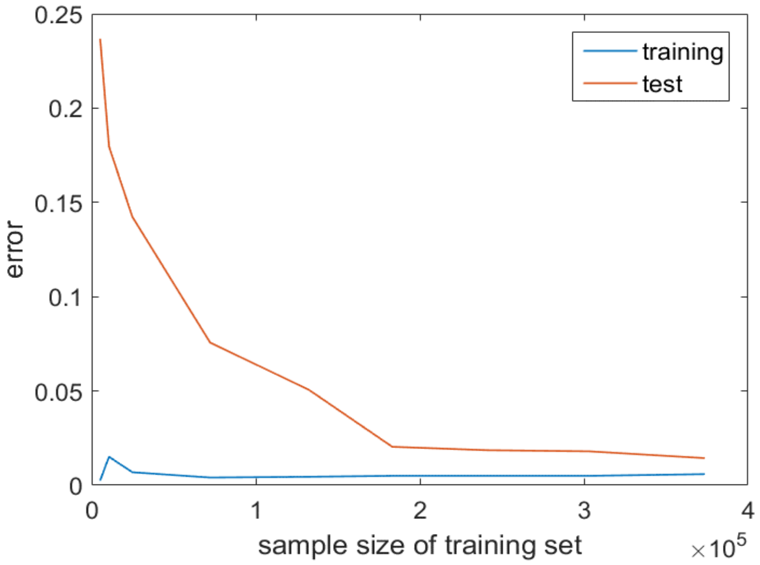

9 GC-MS instrumentation

Supplementary Table 9: GC-MS instrumentation parameters.

Thermal desorption		Gas chromatography		Mass spectrometer	
Parameters	Setting	Parameters	Setting	Parameters	Setting
t - Primary desorption	1 min	***F*** - He carrier gas	2 cm^3 min^{-1}	Scan type	Full scan (+ve)
F - Primary desorption	40 cm^3 min^{-1}	***T*** - Initial	40°C	Mass range	40 to 550 m/z
T - Primary desorption	300°C	***t*** - Initial hold	0 min	Ionisation type	EI
t - Secondary desorption	5 min	***T*** - program	5°C min^{-1} to 300°C	***v*** - scan	3 Hz
F - Secondary desorption	50 cm^3 min^{-1}	***T*** - End	300 °C	***T*** - line temperature	300°C
T - Secondary desorption	300°C	***t*** - End hold	0 min	***T*** - Quadrupole	150°C
F - Cold trap	20 cm^3 min^{-1}	***t*** - Total run	60 min	***T*** - Manifold	230°C
T - Cold trap	-10°C	***T*** - Post run	45°C	***t*** - Solvent delay	5 min
$(\delta T/\delta t)_{trap}$	Max °C min^{-1}	***t*** - Post run	0 min		
T - Trap high	300°C				
t - Trap hold	5 min				
T - Flow path	200°C				
Mode	Spitless				

Note: ***t***, time; ***F***, flow; ***T***, temperature; and ***v***, frequency.
